# The Ants (Hymenoptera, Formicidae) of Sri Lanka: a taxonomic research summary and updated checklist

**DOI:** 10.3897/zookeys.967.54432

**Published:** 2020-09-14

**Authors:** Ratnayake Kaluarachchige Sriyani Dias, Benoit Guénard, Shahid Ali Akbar, Evan P. Economo, Warnakulasuriyage Sudesh Udayakantha, Aijaz Ahmad Wachkoo

**Affiliations:** 1 Department of Zoology and Environmental Management, University of Kelaniya, Sri Lanka University of Kelaniya Kelaniya Sri Lanka; 2 School of Biological Sciences, The University of Hong Kong, Hong Kong SAR, China he University of Hong Kong Hong Kong China; 3 Central Institute of Temperate Horticulture, Srinagar, Jammu and Kashmir, 191132, India Central Institute of Temperate Horticulture Srinagar India; 4 Biodiversity and Biocomplexity Unit, Okinawa Institute of Science and Technology Graduate University, Onna, Okinawa, Japan Okinawa Institute of Science and Technology Graduate University Kunigamigun Japan; 5 Department of Zoology, Government Degree College, Shopian, Jammu and Kashmir, 190006, India Government Degree College Jammu and Kashmir India

**Keywords:** Ants, checklist, endemism, Formicidae, Sri Lanka

## Abstract

An updated checklist of the ants (Hymenoptera: Formicidae) of Sri Lanka is presented. These include representatives of eleven of the 17 known extant subfamilies with 341 valid ant species in 79 genera. *Lioponera longitarsus* Mayr, 1879 is reported as a new species country record for Sri Lanka. Notes about type localities, depositories, and relevant references to each species record are given. Accounts of the dubious and some undetermined species from Sri Lanka are also provided. 82 species (24%) are endemic whereas 18 species that are non-native to Sri Lanka are recorded. The list provides a synthesis of the regional taxonomical work carried out to date and will serve as a baseline for future studies on the ant fauna of this biodiversity hotspot.

## Introduction

Sri Lanka is an island country located in the Indian Ocean, with latitudes ranging from 5°55'N to 9°51'N and longitudes from 79°41'E to 81°53'E. The country has a length of 432 km (268 miles) and a maximum width of 224 km (139 miles), with an area of 65,610 km^2^. Three distinct tropical climatic zones are prevalent in the region, the ‘wet’, ‘dry’, and ‘intermediate zones’ based on seasonal precipitation distribution. These regions receive more than 2,500 mm; less than 1,750 mm, and between 1,750 to 2,500 mm of rain respectively with average annual temperature ranging from 28 °C to 31 °C (Karunaweera et al. 2014).

The island is part of the same shallow continental shelf as India, and is only separated by an inlet of the Bay of Bengal known as the Palk Strait (Pathirana 1980; Somasekaram 1997). This 40 to 85 mile-wide and approximately 85 miles-long strait separates southeastern India and northern Sri Lanka (Vaz 2000). Historically, with high faunal affinities observed across the Palk Strait, the concept of Ceylonese or Lankan biogeographic region was conceived (Wallace 1876; Chandran 1997). More recently, both areas were considered together as the Western Ghats – Sri Lanka biodiversity hotspot, representing a single seasonal wet region in the whole of South Asia (Myers 1988; Mittermeier et al. 2004). This region on the basis of three factors: high numbers of endemics and endemic species/area ratios for both plants and vertebrates, and habitat loss is considered as one of the main hotspots of the world (Myers et al. 2000). Despite several extended periods of land connection during the past 500,000 years, Sri Lanka has maintained a fauna that is largely distinct from that of the Indian mainland (Bossuyt et al. 2004). Unfortunately, this pattern has been tested for only a limited number of taxa in plants, vertebrates, or invertebrates, due to the limitation of data available. The proportion of endemic species in plants is ~ 25%, vertebrates ~ 30%, and invertebrates ~ 43% in the groups studied in depth (Bossuyt et al. 2004; Gunawardene et al. 2007; Gunatilleke et al. 2008). Such variation in endemism and its understanding at finest possible scales will help to develop conservation management programs for the entire region (Gunawardene et al. 2007; Dad et al. 2019).

Sri Lanka is known for its remarkable biodiversity and considered to be one of the richest countries in the Asian region in terms of species concentration with regard to mammals, reptiles, amphibians, fish, and flowering plants (NARESA 1991). The highest species diversity is recorded among the flowering plants (3771 species), followed in decreasing order by the fungi (~ 2260), bryophytes (788), freshwater algae (~ 560) and ferns (314) (Gunatilleke et al. 2008). Among animals, the diversity of vertebrates is well known in comparison to that of invertebrates, where only a few groups have been studied in depth (Gunatilleke et al. 2008). With rapidly decreasing forest cover (Mattsson et al. 2012) more and more species are increasingly threatened; with to this date 27% of birds, 66% of amphibians, 56% of mammals, 49% of freshwater fish, 59% of reptiles, and 44% of flowering plants classified as threatened under the IUCN Red List categories (MOE 2012; CBD 2020). The limited number of trained taxonomists, lack of initiative to explore the biodiversity and the loss of primary forest cover are currently the biggest drawbacks in the conservation of biodiversity in Sri Lanka (Bawa et al. 2007). In particular, knowledge on the entomofauna of Sri Lanka is particularly limited, with comprehensive species checklists only available for a handful of taxonomic groups: such as bees, butterflies and Odonata (Karunaratne et al. 2012; van der Poorten 2012; van der Poorten and Conniff 2012).

Over the past 170 years, the exploration of the ant fauna of Sri Lanka has received sporadic attention and has, since its origin and for long, been dominated by European and later American scientists. Studies of Sri Lankan ants, or including specimens from the country, include in approximate chronological order, were conducted by F. Smith (1853, 1858), Roger (1860–63), Mayr (1862, 1865, 1866, 1868, 1879, 1897), Motschoulsky (1863), Emery (1887a, d, 1893a, b, c, d, 1895a, 1896, 1897b, 1901, 1911, 1912, 1922, 1925), Forel (1892a, b, c, 1893a, b, 1894, 1895a, b, 1900a, b, 1901a, 1902a, b, c, 1903a, b, c, 1904a, 1907a, b, 1908, 1909, 1911a, b, c, e, 1912a, c, d, 1913a, b), Wheeler (1919b, 1942), Karavaiev (1925a, b, c, 1926, 1929, 1933, 1935), Santschi (1928), Donisthorpe (1931, 1941, 1942a, b, c), Menozzi (1935), Chapman and Capco (1951), Brown (1954, 1958–59, 1975, 1978), Wilson et al. (1956), Gregg (1957), Wilson (1958a, b, 1964), Walker (1859–60), Bolton (1974a, b, 1975–77, 1980, 1982, 1987, 1992, 1995, 2000, 2007), Baroni Urbani (1975, 1977a, b), Moffett (1985–86), Baroni Urbani and De Andrade (1994, 2006–07), Rigato (1994), Dorow and Kohout (1995), Way and Bolton (1997), Rickson and Rickson (1998), Schödl (1998), Ward (2001), Seifert (2003), Alpert (2013), Lattke and Delsinne (2016), Seifert et al. (2017) and others. Bingham (1903) was the first author to summarize the ants of Sri Lanka (Ceylon) recording 135 species in 52 genera under five subfamilies within his checklist of ants also including India and Burma.

In recent decades, Sri Lankan authors have contributed to the taxonomic and ecological study of ants including: Dias (2002, 2006a, 2014), Gunawardene et al. (2008, 2010, 2012), Dias and Kosgamage (2012), Dias and Rajapaksa (2016), Dias et al. (2012, 2018) and Yamane and Dias (2016). The information available on ants in Sri Lanka is, however, mainly restricted to a few districts and largely confined to the ‘wet zone’. It is thus highly likely that other climatic zones, which have received less attention, may contribute significantly to the overall regional ant fauna, with possibilities of many new discoveries once properly surveyed.

It should also be noted that several exotic ant species with rampant effect on native mesofauna but mostly undocumented and unappreciated are established in Sri Lanka (Dias et al. 2018). To date, there is no updated and annotated checklist of Sri Lankan ants available and therefore, the objective of the present study is to provide a comprehensive checklist of Sri Lankan ant species, and to highlight gaps where additional faunistic surveys and research are needed to fully understand the diversity of this group in the region.

## Materials and methods

### Data sources

The checklist is primarily based on available literature and few museum records. Most of the names of described species presented are in accordance with the most recent classification following Bolton (2020). Important references to species records are provided.

Species records are presented in function of their mention of examined material within the published publication (primary literature records; e.g., specimen of *Technomyrmex albipes* examined by Bolton and published in Bolton 2007), repetition of known records from other publication (secondary literature records; only the record of a previous publication is being referred to within addition of new material examined), or on the basis of specimens examined here (material examined), inclusive of specimens available on AntWeb (AntWeb records).

Images of type specimens and other AntWeb records are available online on AntWeb and are accessible using the unique ANIC, ANTWEB, CASENT, FMNHINS, FOCOL, MCZ or SAM-HYM-C identifying specimen code.

References to a particular record are arranged according to their presence in primary and secondary literature records.

### Arrangement

Genera and species names are arranged in alphabetical order after being arranged by subfamily. Original descriptions plus local references are listed for all species. The acronyms used for collections are listed below:

**ANIC** Australian National Insect Collection, Canberra, Australia

**CAS** California Academy of Sciences, California, U.S.A.

**DEIC** Deutsches Entomologisches Institut, Müncheberg, Germany

**FMNH** Field Museum of Natural History, Chicago

**HNHM** Hungarian Natural History Museum, Budapest, Hungary

**MCZ** Museum of Comparative Zoology, Cambridge, Massachusetts, U.S.A.

**MHNG** Muséum d’Histoire Naturelle, Geneva, Switzerland

**MNHAH** Museum of Nature and Human Activities Hyogo (Hyogo, Japan)

**MNHN** Muséum national d’Histoire naturelle, Paris, France

**MSNG** Museo Civico di Storia Naturale “Giacomo Doria”, Genova, Italy

**MVMA** Museum Victoria Melbourne, Australia

**MZH** Finnish Museum of Natural History, Helsinki, Finland

**MZLS** Museo Zoologico La Specola, Florence, Italy

**NHMB** Naturhistorisches Museum, Basel, Switzerland

**NHMUK** Natural History Museum, London, United Kingdom

**NHMW** Naturhistorisches Museum, Wien, Austria

**OUMNH** University Museum of Natural History, Oxford, U.K.

**PUAC** Punjabi University Patiala Ant Collection, Punjab, India

**SIZK** Schmalhausen Institute of Zoology, Kyiv, Ukraine

**SMNG** Senckenberg Museum für Naturkunde Görlitz, Görlitz

**SKYC** Seiki Yamane Collection

**UNK** Unknown depository of type material

**ZEMK** Department of Zoology and Environmental Management, University of Kelaniya, Sri Lanka

**ZMHB** Museum für Naturkunde der Humboldt-Universität, Berlin, Germany

**ZMUC** Zoological Museum, University of Copenhagen, Copenhagen, Denmark

**ZMUK** Zoologisches Museum, Universität Kiel, Germany

**ZSM** Zoologische Staatssammlung München, Munich, Germany

## Results and discussion

The checklist includes 341 valid species/subspecies belonging to 79 genera under eleven subfamilies, representing ca. 2.2% of the global ant diversity (Janicki et al. 2016; Guénard et al. 2017; Bolton 2020). The eleven subfamilies recorded include; Amblyoponinae; Aneuretinae; Dolichoderinae; Dorylinae; Ectatomminae; Formicinae; Leptanillinae; Myrmicinae; Ponerinae; Proceratiinae; Pseudomyrmecinae with details on species and genus level richness presented in Table 1. The most diverse genera are *Camponotus* (41 species and subspecies), *Polyrhachis* (34), *Pheidole* (27), *Crematogaster* (21), *Tetramorium* (16), *Carebara* (12) and *Leptogenys* (11). Three subfamilies; Formicinae; Myrmicinae, and Ponerinae, together represent more than 86% to the regional ant diversity with more than 40% of species richness found within the Myrmicinae subfamily alone.

**Table 1.** Diversity of subfamilies, genera, and species known from Sri Lanka.

Subfamily	Genus	Species (subspecies)
Amblyoponinae (3 genera, 3 species)	* Myopopone *	1
	* Prionopelta *	1
	* Stigmatomma *	1
Aneuretinae (1 genus, 1 species)	* Aneuretus *	1
Dolichoderinae (6 genera, 13 species & 1 subspecies)	* Chronoxenus *	1
	* Dolichoderus *	2 (1)
	* Iridomyrmex *	1
	* Ochetellus *	1
	* Tapinoma *	3
	* Technomyrmex *	5
Dorylinae (6 genera, 19 species)	* Aenictus *	7
	* Dorylus *	2
	* Lioponera *	2
	* Ooceraea *	4
	* Parasyscia *	3
	* Syscia *	1
Ectatomminae (1 genus, 2 species)	* Gnamptogenys *	2
Formicinae (13 genera, 81 species & 21 subspecies)	* Acropyga *	2
	* Anoplolepis *	1
	* Camponotus *	28 (13)
	* Colobopsis *	2
	* Lepisiota *	6 (1)
	* Myrmoteras *	1
	* Nylanderia *	6 (1)
	* Oecophylla *	1
	* Paratrechina *	1
	* Plagiolepis *	3
	* Polyrhachis *	28 (6)
	* Prenolepis *	1
	* Pseudolasius *	1
Leptanillinae (3 genera, 3 species)	* Leptanilla *	1
	* Protanilla *	1
	* Yavnella *	1
Myrmicinae (29 genera, 126 species & 12 subspecies)	* Acanthomyrmex *	1
	* Anillomyrma *	1
	* Aphaenogaster *	1
	* Calyptomyrmex *	3
	* Cardiocondyla *	5
	* Carebara *	11 (1)
	* Cataulacus *	4
	* Crematogaster *	16 (5)
	* Dilobocondyla *	1
	* Erromyrma *	1
	* Lophomyrmex *	3
	* Meranoplus *	5
	* Metapone *	1
	* Monomorium *	6
Myrmicinae (29 genera, 126 species & 12 subspecies)	* Myrmecina *	1
	* Myrmicaria *	2
	* Paratopula *	1
	* Pheidole *	22 (5)
	* Pristomyrmex *	2
	* Recurvidris *	2
	* Rhopalomastix *	2
	* Solenopsis *	2
	* Stereomyrmex *	1
	* Strumigenys *	6
	* Syllophopsis *	1
	* Tetramorium *	16
	* Trichomyrmex *	7 (1)
	* Tyrannomyrmex *	1
	* Vollenhovia *	1
Ponerinae (15 genera, 47 species & 7 subspecies)	* Anochetus *	8
	* Bothroponera *	3
	* Brachyponera *	3
	* Centromyrmex *	1 (1)
	* Cryptopone *	1
	* Diacamma *	4 (2)
	* Harpegnathos *	1 (2)
	* Hypoponera *	8
	* Leptogenys *	10 (1)
	* Mesoponera *	1
	* Myopias *	1
	* Odontomachus *	1
	* Parvaponera *	1
	* Platythyrea *	2
	* Pseudoneoponera *	2 (1)
Proceratiinae (1 genus, 1 species)	* Discothyrea *	1
Pseudomyrmecinae (1 genus, 4 species)	* Tetraponera *	4

**Figure 1.**
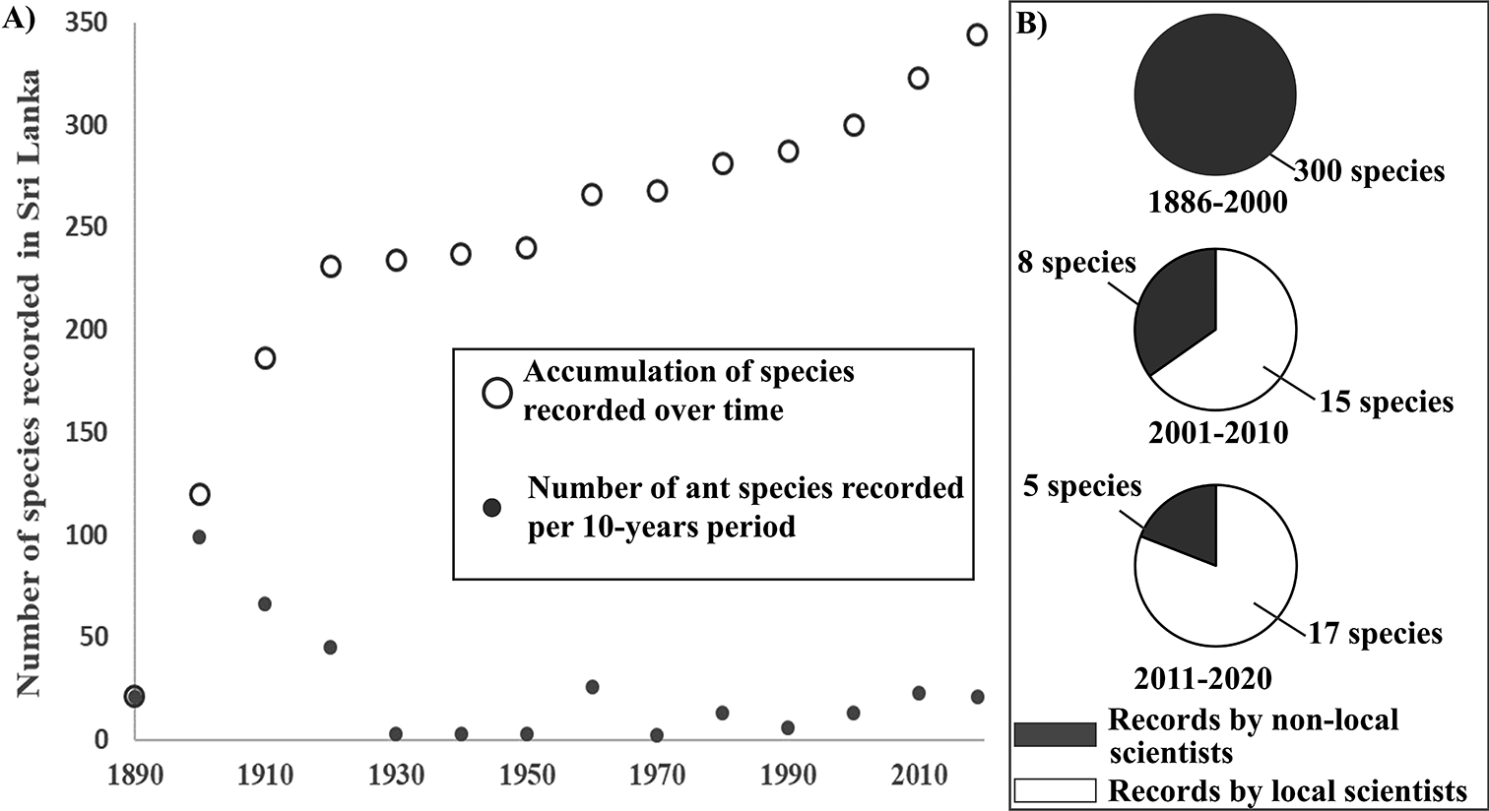
**A** Rate of species recording in Sri Lanka per decade from 1886 to 2020, and **B** number of species recorded in Sri Lanka per period in function of the origins of the authors contributing to these discoveries. The last two decades are shown separately as they present a change in species discovery (two species are not included as time of discovery is unknown).

The exploration of the Sri Lankan ant fauna and descriptions of species has, however, been relatively limited during the past few decades. For instance, more than a century ago, by 1920, 66.8% of the diversity currently known had already been recorded (Fig. 1). This number increased slowly for 80 years with the addition of 69 newly recorded species (19.9% of the total fauna); and slightly faster in the most recent 20 years with an addition of 46 species (12.7%) mainly through the work of local scientists (Fig. 1A, B). The taxonomic work still required on Sri Lankan ants is likely to be important due to the high number of species and subspecies recorded more than a century ago, at a time when species descriptions were sometimes incomplete or species boundaries poorly defined, later leading to potential misidentifications (see Table 4). As a result, work on regional exploration and the use of new sampling are both needed, as shown by the paucity of certain diverse genera (e.g., *Colobopsis*, *Myrmecina*, *Stigmatomma*, *Strumigenys*), while taxonomic revisions of specimens for the region based on newly collected material to confirm past identifications or to help in the descriptions of new species should help in providing a more complete overview of the diversity of the Sri Lankan myrmecofauna.

**Figure 2.**
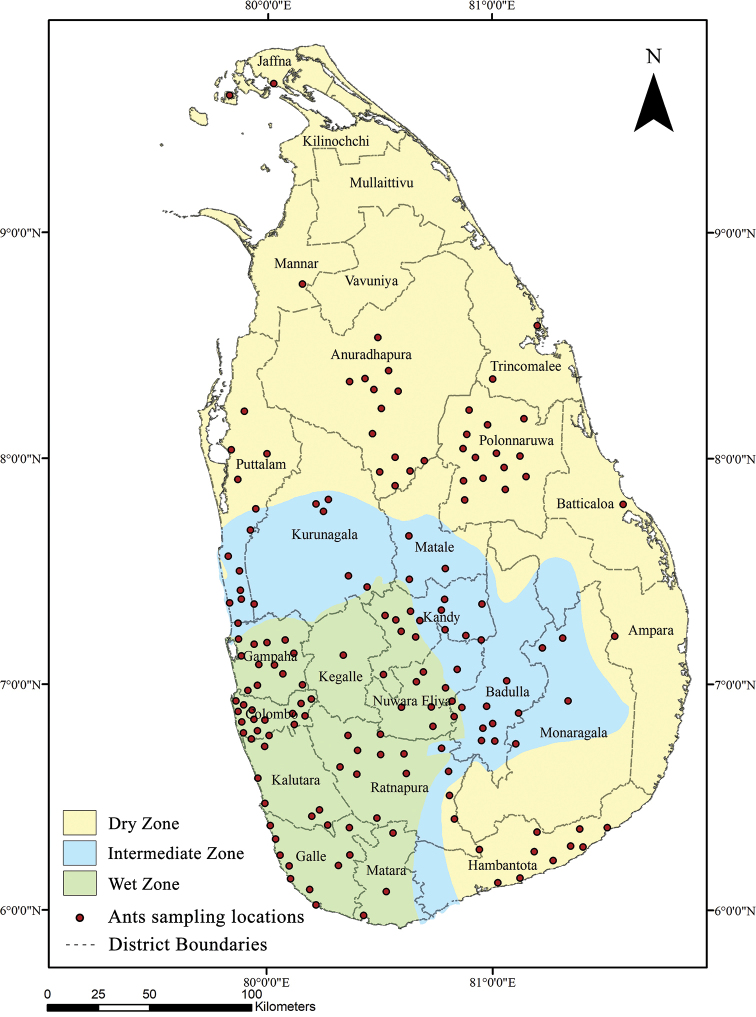
Map of Sri Lanka with ant sampling localities in different zones.

Of the three traditionally distinct climatic zones in Sri Lanka: ‘dry’ (~ 40669 km^2^), ‘intermediate’ (~ 9670 km^2^) and the ‘wet zone’ (~ 15267 km^2^), most of the ant surveys and species occurrence have been reported from the ‘wet zone’ areas, which include some of the well protected and intact forest regions of the country (Fig. 2; Suppl. material 2). The ‘wet zone’ supports the greatest diversity (231 species), followed by the ‘dry’ (113 species), and the ‘intermediate zone’ (71 species). It should be noted that most of the northern and eastern regions of the country, located in the dry zone, have received limited sampling coverage, and that future surveys should focus on these regions. Sixty-four species recorded from Sri Lanka could not be assigned to any zone as they lack precise location information within the country underlying, here again, the paucity of surveys since the time of their original recording in Sri Lanka and the need for future and further research in the various regions of the country.

### Endemic species

Sri Lanka is known to have diverse vegetation types and a distinctive fauna characterized by a high degree of endemicity (Gunawardene et al. 2007; Gunatilleke et al. 2008). Of the 341 ant species/subspecies present in Sri Lanka, only 82 species (24%) are considered as endemic (Table 2). Thanks to recent global compilation and regional work on ant distribution, the number of species identified as endemic species to Sri Lanka has greatly increased in comparison of previous work in which only 33 species (17% of 194 species) had been identified (Dias et al. 2012). It should be noted, nonetheless, that the current level of endemism retrieved in ants is relatively low in comparison of flowering plants (28%), odonates (48%), reptiles (59%), land snails (83%), and amphibians (85%) (Gunawardene et al. 2007; Gunatilleke et al. 2008; MOE 2012). Potentially, a similar pattern of high species richness and endemism might be likely for ants, but further comprehensive surveys in addition to thorough taxonomic work are first needed to fully depict this pattern. However, the current legal framework for biodiversity conservation allowing the export of specimens outside Sri Lanka is very restrictive and might represent a serious limitation to the completion of the taxonomic work that could be undertaken within the country. Moreover, with ongoing landscape modification in the region, many species have been driven to critical status (Somaweera et al. 2015; Perera et al. 2017; Karawita et al. 2018), therefore, urgent, large-scale, and sustained eﬀorts to monitor, characterize, and conserve the ant fauna of Sri Lanka is critical.

**Table 2.** List of the 82 endemic ants in Sri Lanka.

** Aneuretinae **	*Carebara sinhala* Fischer, Azorsa & Fisher, 2014
*Aneuretus simoni* Emery, 1893	*Crematogaster apicalis* Motschoulsky, 1863
** Dorylinae **	*Crematogaster brunnescens* Motschoulsky, 1863
*Aenictus biroi* Forel, 1907	*Crematogaster consternens* (Walker, 1859)
*Ooceraea coeca* Mayr, 1897	*Crematogaster desecta* Forel, 1911
*Ooceraea fragosa* Roger, 1862	*Crematogaster dohrni gigas* Forel, 1913
*Parasyscia luteoviger* (Brown, 1975)	*Crematogaster pellens* Walker, 1859
** Ectatomminae **	*Crematogaster rogeri* Emery, 1922
*Gnamptogenys sinhala* Lattke, 2016	*Crematogaster rothneyi haputalensis* Forel, 1913
** Formicinae **	*Dilobocondyla didita* (Walker, 1859)
*Camponotus albipes* Emery, 1893	*Meranoplus boltoni* Schödl, 1998
*Camponotus auriculatus* Mayr, 1897	*Meranoplus loebli* Schödl, 1998
*Camponotus fletcheri* Donisthorpe, 1942	*Metapone greeni* Forel, 1911
*Camponotus greeni* Forel, 1911	*Monomorium taprobanae* Forel, 1913
*Camponotus latebrosus* (Walker, 1859)	*Pheidole barreleti* Forel, 1903
*Camponotus mendax integer* Forel, 1895	*Pheidole ceylonica* (Motschoulsky, 1863)
*Camponotus ominosus* Forel, 1911	*Pheidole diffidens* (Walker, 1859)
*Camponotus reticulatus yerburyi* Forel, 1893	*Pheidole gracilipes* (Motschoulsky, 1863)
*Camponotus sesquipedalis* Roger, 1863	*Pheidole horni* Emery, 1901
*Camponotus simoni* Emery, 1893	*Pheidole latinoda peradeniyae* Forel, 1911
*Camponotus variegatus intrans* Forel, 1911	*Pheidole rugosa* Smith F, 1858
*Camponotus wedda* Forel, 1908	*Pheidole sulcaticeps vellicans* Forel, 1911
*Myrmoteras ceylonicum* Gregg, 1957	*Pheidole templaria euscrobata* Forel, 1913
*Nylanderia taylori levis* (Forel, 1913)	*Pristomyrmex sinharaja* Dias & Yamane, 2016
*Nylanderia vagabunda* (Motschoulsky, 1863)	*Rhopalomastix escherichi* Forel, 1911
*Plagiolepis pissina* Roger, 1863	*Stereomyrmex horni* Emery, 1901
*Polyrhachis bugnioni* Forel, 1908	*Strumigenys inopinata* (De Andrade, 1994)
*Polyrhachis convexa isabellae* Forel, 1908	*Strumigenys veddha* De Andrade, 2007
*Polyrhachis gibbosa* Forel, 1908	*Tetramorium curvispinosum* Mayr, 1897
*Polyrhachis nigra* Mayr, 1862	*Tetramorium transversarium* Roger, 1863
*Polyrhachis sophocles* Forel, 1908	*Trichomyrmex emeryi laevior* (Mayr, 1897)
*Polyrhachis tibialis pectita* Santschi, 1928	*Trichomyrmex rogeri* Mayr, 1865
*Polyrhachis xanthippe* Forel, 1911	*Tyrannomyrmex legatus* Alpert, 2013
*Polyrhachis yerburyi* Forel, 1893	*Vollenhovia escherichi* Forel, 1911
** Leptanillinae **	** Ponerinae **
*Leptanilla besucheti* Baroni Urbani, 1977	*Anochetus consultans* (Walker, 1859)
*Protanilla schoedli* Baroni Urbani and De Andrade, 2006	*Anochetus longifossatus* Mayr, 1897
** Myrmicinae **	*Anochetus nietneri* (Roger, 1861)
*Calyptomyrmex singalensis* Baroni Urbani, 1975	*Anochetus pangens* (Walker, 1859)
*Calyptomyrmex tamil* Baroni Urbani, 1975	*Harpegnathos saltator taprobanae* Forel, 1909
*Carebara butteli* (Forel, 1913)	*Hypoponera taprobanae* (Forel, 1913)
*Carebara ceylonensis* (Forel, 1911)	*Leptogenys exundans* (Walker, 1859)
*Carebara deponens* (Walker, 1859)	*Leptogenys meritans* (Walker, 1859)
*Carebara diversa taprobanae* (Smith F, 1858)	*Pseudoneoponera rufipes ceylonensis* (Forel, 1911)
*Carebara escherichi* (Forel, 1911)	

### Non-native species

The current list of introduced and established species in Sri Lanka includes 18 species largely dominated by the Myrmicinae (13) and completed by the Ponerinae (3) and Formicinae (2) subfamilies (Table 3). The ecological impacts of non-native and invasive ant species with rampant effect on native mesofauna have not been studied in Sri Lanka, but the well-known effects of some of these species in other regions of the world (Wittman 2014), including within Asia (Wong et al. 2020), may hint towards similar outcomes. Therefore, more eﬀorts should be directed to evaluate the distribution, ecology, and various impacts of non-native species. Potential threat of non-native species on native species, particularly endemics should be evaluated to safeguard the native ant fauna. Finally, the limited efforts in the study of Sri Lankan ants suggests that more non-native species could be discovered in future studies, or through biogeographic studies aiming at determining more precisely the native from the introduced ranges of species widespread within Asia and beyond.

**Table 3.** List of non-native ants in Sri Lanka. Species with an asterisk * are considered as invasive in other regions of the world.

Formicinae (2 species)	Myrmicinae (13 species)	Ponerinae (3 species)
*Nylanderia vividula*	*Cardiocondyla emeryi*	*Hypoponera punctatissima*
*Paratrechina longicornis*	*Monomorium monomorium*	*Hypoponera ragusai*
	*Monomorium pharaonis*	*Leptogenys falcigera*
	*Monomorium subopacum*	
	Pheidole megacephala*	
	*Solenopsis geminata*	
	*Strumigenys emmae*	
	*Strumigenys membranifera*	
	*Syllophopsis australica*	
	*Tetramorium bicarinatum*	
	*Tetramorium pacificum*	
	*Tetramorium simillimum*	
	*Tetramorium tonganum*	

### Misidentifications and dubious/erroneous records

To correct the errors cited in earlier literature so as to reduce the taxonomic confusion by eliminating misinformation associated with Sri Lankan ants, 58 ant taxa previously reported from the country are here marked as dubious based on either erroneous data in terms of misidentifications, misspellings, erroneous locality, or potential occurrence. A brief explanation is provided about their dubious status (Table 4).

**Table 4.** Dubious/unverified records of ants in Sri Lanka.

Name	References	Explanation
** Amblyoponinae **		
*Prionopelta nominata* (Smith F, 1871)	Chapman and Capco 1951: 27 (Ceylon)	Australian: Possible misidentification of *Prionopelta kraepelini* Forel, 1905
*Stigmatomma testaceum* (Motschoulsky, 1863)	Motschoulsky 1863: 15 (Ceylon)	This species is a *nomen nudum* and thus its recollection is difficult due to a lack of sufficient description to identify the species
** Dolichoderinae **		
*Chronoxenus myops* (Forel, 1895)	Li-Zhong 2006: 263 (Sri Lanka)	No specimen base to confirm distribution in Sri Lanka
*Chronoxenus walshi* (Forel, 1895)	Li-Zhong 2006: 263 (Sri Lanka)	No specimen base to confirm distribution in Sri Lanka
*Iridomyrmex chasei* Forel, 1902	Forel 1908: 3 (Pattipola, Ceylon), Chapman and Capco 1951: 189 (Ceylon), Dias 2002: 19 (Sri Lanka)	Australian: disjunctive distribution, needs confirmation in Sri Lanka
*Technomyrmex modiglianii* Emery, 1900	Dias 2002: 19 (Sri Lanka)	Wrongly included for *Technomyrmex elatior* Forel, 1902
** Dorylinae **		
*Aenictus aratus* Wheeler & Chapman, 1930	Dias 2002: 17 (Sri Lanka), Rajan et al. 2006: 166 (Sri Lanka)	Australian: extra Australian specimens being referable to *Aenictus aitkenii* Forel, 1901 (Shattuck, 2008)
*Aenictus binghami* Forel, 1900	Gunawardene et al. 2008: 79 (Sinharaja Forest Reserve), Gunawardene et al. 2012: 84 (Sinharaja Forest Reserve)	Unlikely in Sri Lanka
*Cerapachys sulcinodis* Emery, 1889	Li-Zhong 2006: 263 (Sri Lanka), Dias and Rajapaksa 2016: 34 (Kurunegala)	No specimen base to confirm distribution in Sri Lanka
*Dorylus laevigatus* (Smith F, 1857)	Dias et al. 2012: 17 (Sri Lanka), Dias 2014: 95 (Sri Lanka), Dias and Rajapaksa 2016: 34 (Anuradhapura, Kurunegala, Polonnaruwa)	No specimen base to confirm distribution in Sri Lanka
** Formicinae **		
*Camponotus albosparsus* Bingham, 1903	Chapman and Capco 1951: 243 (Ceylon)	Unlikely in Sri Lanka
*Camponotus angusticollis sanguinolentus* Forel, 1895	Sheela 2008a: 11 (Sri Lanka)	No specimen base to confirm distribution in Sri Lanka
*Camponotus horni* Clark, 1930	Forel 1903a: 712 (Nalanda), Dias 2002: 18 (Sri Lanka)	Australian: disjunctive distribution, needs confirmation in Sri Lanka
*Camponotus maculatus* (Fabricius, 1782)	Dias 2006a: 50 (Sri Lanka)	No specimen base to confirm distribution in Sri Lanka
*Camponotus mayri* Forel, 1879	Li-Zhong 2006: 264 (Sri Lanka)	Afrotropical: no specimen base to confirm distribution in Sri Lanka
*Camponotus nirvanae* Forel, 1893	AntWeb 2020 (Ceylon: CASENT 0910542)	Forel (1893) did not list Ceylon as the type locality for any of the syntypes. Locality label seems ambiguous.
*Camponotus sericeus peguensis* Emery, 1895	Ceylon (Emery 1925: 126), Chapman and Capco 1951: 242 (Ceylon)	Unlikely in Sri Lanka
*Camponotus sericeus sanguiniceps* Donisthorpe, 1942	Chapman and Capco 1951: 242 (Ceylon)	Unlikely in Sri Lanka
*Echinopla striata aciculata* (Smith F, 1858)	Chapman and Capco 1951: 284 (Ceylon)	Unlikely in Sri Lanka
*Formica fuscicauda* Motschoulsky, 1863	Motschoulsky 1863: 12 (Ceylon), Emery 1925: 270 (Ceylon)	*Incertae sedis* in genus, unidentifiable
*Formica subpicea* Motschoulsky, 1863	Motschoulsky 1863: 12 (Ceylon), Emery 1925: 270 (Ceylon)	*Incertae sedis* in genus, unidentifiable
*Lepisiota modesta* (Forel, 1894)	Dias and Kosgamage 2012: 61 (Anuradhapura, Polonnaruwa)	No specimen base to confirm distribution in Sri Lanka
*Myrmoteras binghamii* Forel, 1893	Dias 2006a: 50 (Sri Lanka), Gunawardene et al. 2008: 80 (Sinharaja Forest Reserve), Gunawardene et al. 2012: 84 (Sinharaja Forest Reserve)	Needs confirmation in Sri Lanka (Bui et al. 2013)
*Paraparatrechina minutula* (Forel, 1901)	Gunawardene et al. 2012: 83 (Sinharaja Forest Reserve)	Australian: disjunctive distribution, needs confirmation in Sri Lanka
*Polyrhachis bellicosa* Smith F, 1859	Dias and Rajapaksa 2016: 35 (Anuradhapura, Colombo, Galle, Gampaha, Polonnaruwa, Ratnapura)	No specimen base to confirm distribution in Sri Lanka
*Polyrhachis cingula* Donisthorpe, 1947	AntWeb 2020 (Ceylon, Peradeniya: CASENT 0912101)	This is a misidentification. Clearly the two specimens on Antweb do not show the same species and the individual from New Guinea is a type.
*Polyrhachis hippomanes* Smith F, 1861	Dias 2002: 19 (Sri Lanka)	Wrongly included for *Polyrhachis hippomanes ceylonensis* Emery, 1893
*Polyrhachis moesta* Emery, 1887	Li-Zhong 2006: 271 (Sri Lanka)	No specimen base to confirm distribution in Sri Lanka
*Polyrhachis spinigera* Mayr, 1879	Emery 1893a: 254 (Kandy)	Probably a misidentification of *Polyrhachis lacteipennis* Smith F, 1858. Emery (1901) listed *P. lacteipennis* as a denizen of Sri Lanka, without any reference to *P. spinigera* Mayr, 1879
*Polyrhachis ypsilon* Emery, 1887	Forel 1893a: 31 (Ceylon), Emery 1925: 182 (Ceylon)	Needs confirmation in Sri Lanka (record absent in Kohout 2014)
*Pseudolasius familiaris* (Smith F, 1860)	Gunawardene et al. 2008: 80 (Sinharaja Forest Reserve), Gunawardene et al. 2012: 84 (Sinharaja Forest Reserve)	Distribution in Sri Lanka seems far remote from the known distribution of this species in Asia
** Myrmicinae **		
*Carebara affinis spinosior* (Forel, 1911)	Forel 1913b: 662 (Nalanda)	Probably a misidentification. Needs further confirmation in Sri Lanka
*Cardiocondyla nuda* Mayr, 1866	Kugler 1984: 11 (Ceylon), Mathew and Tiwari 2000: 306 (Sri Lanka), Dias 2002: 18 (Sri Lanka), Tiwari et al. 2003: 492 (Sri Lanka); Dias 2006a: 51 (Sri Lanka), Ghosh et al. 2006: 386 (Sri Lanka), Li-Zhong 2006: 265 (Sri Lanka), Rajan et al. 2006: 174 (Sri Lanka), Gunawardene et al. 2008: 81 (Sinharaja Forest Reserve), Amarasinghe 2010: 12 (Nawalapitiya), Dias et al. 2012: 15 (Sri Lanka), Dias and Kosgamage 2012: 62 (Hathamuna, Somawathiya Sanctuary), Dias 2014: 164 (Sri Lanka), Dias and Ruchirani 2014: 88 (Kuluna Kanda Proposed Forest Reserve), Dias and Rajapaksa 2016: 35 (Anuradhapura, Polonnaruwa), Dias and Udayakantha 2016b: 5 (Indikada Mukalana Forest Reserve), Udayakantha and Dias 2018: 72 (Indikada Mukalana Forest Reserve)	Misidentification of *Cardiocondyla kagutsuchi* Terayama, 1999 (Seifert, 2003)
*Crematogaster politula* Forel, 1902	Dias et al. 2012: 15 (Sri Lanka), Dias and Rajapaksa 2016: 35 (Puttalam)	No specimen base to confirm distribution in Sri Lanka
*Crematogaster treubi* Emery, 1896	Dias 2002: 18 (Sri Lanka), Hosoishi and Ogata 2009: 62 (Sri Lanka)	No specimen base to confirm distribution in Sri Lanka
*Lophomyrmex ambiguus* Rigato, 1994	Sheela and Ghosh 2008 (Sri Lanka)	No specimen base to confirm distribution in Sri Lanka
*Monomorium chinense* Santschi, 1925	Li-Zhong 2006: 268 (Sri Lanka)	No specimen base to confirm distribution in Sri Lanka
*Monomorium salomonis* (Linnaeus, 1758)	Magretti 1884: 540 (Ceylon)	Needs confirmation of distribution in Oriental region
*Myrmecina curtisi* Donisthorpe, 1949	Gunawardene et al. 2008: 81 (Sinharaja Forest Reserve)	Australian: possible misidentification of *Myrmecina striata* Emery, 1889
*Myrmica obscurata* Motschoulsky, 1863	Motschoulsky 1863: 16 (Ceylon)	*Incertae sedis* in genus, unidentifiable
*Myrmica pilinodis* Motschoulsky, 1863	Motschoulsky 1863: 16 (Ceylon)	*Incertae sedis* in genus, unidentifiable
*Pheidole templaria* Forel, 1902	Dias 2002: 17 (Sri Lanka), Dias 2006a: 52 (Sri Lanka), Dias et al. 2012: 16 (Sri Lanka)	Wrongly included for *Pheidole templaria euscrobata* Forel, 1913
*Rogeria* sp.	Gunawardene et al. 2008: 75 (Sinharaja Forest Reserve), Gunawardene et al. 2010: 558 (Sinharaja Forest Reserve)	Probably a misidentification. The genus is known only from the Nearctic, Neotropical, and Oceanian realms
*Strumigenys lewisi* Cameron, 1886	Forel 1903a: 707 (Ceylon), Emery 1897a: 574 (Ceylon)	Unlikely in Sri Lanka
*Tetramorium flavipes* Emery, 1893	Chapman and Capco 1951: 180 (Ceylon)	Unlikely in Sri Lanka
*Tetramorium guineense* (Bernard, 1953)	Forel 1911a: 225 (Seenigoda), Dias, 2002: 18 (Sri Lanka).	Should be *Tetramorium bicarinatum* (Nylander, 1846) (Bolton, 1977)
*Tetramorium nodiferum* (Emery, 1901)	Emery 1912: 104 (Ceylon).	Afrotropical: disjunctive distribution, needs confirmation in Sri Lanka
*Trichomyrmex emeryi* (Mayr, 1895)	Dias 2002: 18 (Sri Lanka).	Wrongly included for *Trichomyrmex emeryi laevior* (Mayr, 1897)
** Ponerinae **		
*Diacamma geometricum* (Smith F, 1857)	Li-Zhong 2006: 266 (Sri Lanka)	No specimen base to confirm distribution in Sri Lanka. Reported so far from SE Asia only (Laciny et al. 2015)
*Diacamma rugosum celebense* Emery, 1887	Emery 1897b: 156 (Ceylon), Forel 1900b: 319 (Ceylon)	Probably a misidentification. Needs confirmation of distribution in Sri Lanka
*Diacamma vagans* (Smith F, 1860)	Emery 1887d: 440 (Pointe de Galle), Mukherji and Ribeiro 1925: 205 (Ceylon), Chapman and Capco 1951: 59 (Ceylon), Ali 1991: 3 (Sri Lanka), Tiwari 1999: 25 (Sri Lanka)	Could be a different species (Laciny et al. 2015)
*Hypoponera truncata* (Smith F, 1860)	Gunawardene et al. 2008: 83 (Sinharaja Forest Reserve)	Personal communication Barry Bolton 2 July 2012
*Odontomachus haematodus* (Linnaeus, 1758)	Emery 1893a: 243 (Kandy, Colombo, Nuwara Eliya), Emery 1901: 113 (Ceylon), Emery 1911: 114 (Ceylon), Forel 1900a: 58 (Ceylon), Forel 1908: 2 (Puwakpitiya, Galle), Forel 1911a: 215 (Ceylon), Viehmeyer 1912: 18 (Ceylon), Karavaiev 1926: 417 (Kandy), Chapman and Capco 1951: 43 (Ceylon), Ali 1991: 4 (Sri Lanka), Tiwari 1999: 21 (Sri Lanka), Mathew and Tiwari 2000: 289 (Sri Lanka), Dias 2002: 19 (Sri Lanka), Tiwari et al. 2003: 474 (Sri Lanka), Amarasinghe 2010: 12 (Nawalapitiya).	Should be *Odontomachus simillimus* Smith F, 1858
*Odontomachus monticola* Emery, 1892	Li-Zhong 2006: 269 (Sri Lanka).	No specimen base to confirm distribution in Sri Lanka
*Odontoponera transversa* (Smith F, 1857)	Li-Zhong 2006: 269 (Sri Lanka).	No specimen base to confirm distribution in Sri Lanka
** Pseudomyrmecinae **		
*Tetraponera aitkenii* (Forel, 1902)	Ali 1992: 1 (Sri Lanka), Tiwari 1999: 34 (Sri Lanka), Dias 2002: 17 (Sri Lanka)	Personal communication Phil Ward 18 August 2015
*Tetraponera attenuata* Smith F, 1877	Gunawardene et al. 2008: 83 (Sinharaja Forest Reserve), Gunawardene et al. 2012: 83 (Sinharaja Forest Reserve).	Personal communication Phil Ward 18 August 2015
*Tetraponera difficilis* (Emery, 1900)	Gunawardene et al. 2008: 83 (Sinharaja Forest Reserve).	Personal communication Phil Ward, 18 August 2015

## Checklist

### AMBLYOPONINAE

#### *Myopopone*: 1 species

***Myopopone castanea* (Smith F, 1860)**

*Amblyopone castaneus* Smith F, 1860a: 105. Type locality (TL): [Bac.] Bacan, Maluku Utara: Indonesia [Syntype: OUMNH]. [Images of CASENT 0901371 syntype worker examined].

**Distribution.** Wet and Dry Zones; *Primary literature records*: Kottawa (Emery 1893a: 240), Ceylon (Forel 1900a: 54), Peradeniya (Forel 1913a: 5), Trincomalee (Donisthorpe 1942a: 30), Sinharaja Forest Reserve (Gunawardene et al. 2008: 79); *Secondary literature records*: Ceylon (Bingham 1903: 34), Ceylon (Emery 1911: 26), Ceylon (Wheeler 1919a: 50), Ceylon (Wheeler and Chapman 1925: 57), Ceylon (Chapman and Capco 1951: 23), Ceylon (Brown 1960: 173), Sri Lanka (Dias 2002: 19), Sri Lanka (Dias 2006a: 52), Sri Lanka (Xu and He 2011: 234), Sri Lanka (Dias et al. 2012: 18); *AntWeb records*: Hantana, Kandy: ANIC 32-026167, CASENT 0102523, CASENT 0104580, CASENT 0104581, CASENT 0752184 (AntWeb 2020).

#### *Prionopelta*: 1 species

***Prionopelta kraepelini* Forel, 1905**

*Prionopelta kraepelini* Forel, 1905: 3. TL: Tjompea, near Bogor, Java: Indonesia [Syntype: MHNG].

**Distribution.** Wet Zone; *Primary literature records*: Pompekelle, Ratnapura (Dias et al. 2018: 452).

#### *Stigmatomma*: 1 species

***Stigmatomma bellii* (Forel, 1900)**

*Amblyopone bellii* Forel, 1900a: 55. TL: [Kanara], Karnataka: India [Holotype: MHNG]. [Images of CASENT 0102510 holotype worker examined].

**Distribution.** Dry Zone; *Material examined*: 1 worker [ZEMK], Puttalam District, Panirendawa Forest, 7°33'N, 79°53'E, 23.iii.2009 (leg. H.A.W.S. Peiris); *AntWeb records*: Southern Palatupana, near entrance Yala National Park: CASENT 0172186 (Antweb 2020).

### ANEURETINAE

#### *Aneuretus*: 1 species

***Aneuretus simoni* Emery, 1893**

*Aneuretus simoni* Emery, 1893b: cclxxvi. TL: Kandy: Sri Lanka [Syntype: MSNG]. [Images of CASENT 0905041 syntype worker examined].

**Distribution.** Wet and Intermediate Zones; *Primary literature records*: Kandy (Emery 1893a: 242), Ceylon (Emery 1893b: cclxxvi), Ceylon (Forel 1895a: 462), Ceylon (Forel 1912a: 771), Ceylon (Forel 1913a: 88), Kandy, Peradeniya, Adam’s Peak Forest Reserves, Gilimale, Ratnapura (Wilson et al. 1956: 95), Gilimale (Jayasuriya and Traniello 1985: 366), Sri Lanka (Traniello and Jayasuriya 1985: 376), Sri Lanka (Shattuck 1994: 1), Ratnapura (Dias 2006a: 45), Sinharaja Forest Reserve (Gunawardene et al. 2008: 79), Gilimale Forest Reserve (Dias and Perera 2011: 73), Kirikanda (Dias et al. 2011: 99), Sinharaja Forest Reserve (Gunawardene et al. 2012: 84), Kirikanda Forest (Dias et al. 2013: 64), Moraella, Rambukoluwa (Karunarathna and Karunaratne 2013: 4606), Sri Lanka (Dias 2014: 58), Kalugala Proposed Forest Reserve, Kuluna Kanda Proposed Forest Reserve, Wilpita “Aranya Kele” (Dias and Ruchirani 2014: 88), Meethirigala Forest Reserve (Udayakantha and Dias 2015: 31), Colombo, Galle, Gampaha, Ratnapura, Kalutara (Dias and Rajapaksa 2016: 33, 38), Meethirigala Forest Reserve (Dias and Udayakantha 2016a: 53), Indikada Mukalana Forest Reserve (Dias and Udayakantha 2016b: 5), Indikada Mukalana Forest Reserve (Udayakantha and Dias 2018: 68); *Secondary literature records*: Ceylon (Emery 1913: 7), Ceylon (Chapman and Capco 1951: 181), Sri Lanka (Dias 2002: 19), Sri Lanka (Dias 2006b: 8), Sri Lanka (Dias et al. 2012: 15), Sri Lanka (Boudinot 2015: 17); *AntWeb records*: Kandy, Sinharaja Forest Reserve, Sabaragamuwa, Gilimale: ANTWEB 1008503, CASENT 0007014, CASENT 0010853, CASENT 0102369, CASENT 0102370, CASENT 0172258–2259, CASENT 0637363, CASENT 0905041 (AntWeb 2020).

### DOLICHODERINAE

#### *Chronoxenus*: 1 species

***Chronoxenus wroughtonii* (Forel, 1895)**

*Bothriomyrmex wroughtonii* Forel, 1895a: 470. TL: [Kanara], Karnataka: India [Lectotype: NHMB]. [Images of CASENT 0911493 lectotype worker examined].

**Distribution.** Wet Zone; *Primary literature records*: Ceylon (Forel 1895a: 470), Sri Lanka (Shattuck 1994: 37), South Sri Lanka (Way and Bolton 1997: 443), Sinharaja Forest Reserve (Gunawardene et al. 2008: 79); *Secondary literature records*: Ceylon (Emery 1913: 29), Ceylon (Chapman and Capco 1951: 187), Sri Lanka (Dias 2002: 19), Sri Lanka (Li-Zhong 2006: 263), Sri Lanka (Rajan et al. 2006: 168).

#### *Dolichoderus*: 3 species/subspecies

***Dolichoderus taprobanae* (Smith F, 1858)**

*Formica taprobane* Smith F, 1858: 13. TL: [Ceylon] Sri Lanka [Holotype: NHMUK]. [Images of CASENT 0902971 holotype queen examined].

**Distribution.** Wet Zone; *Primary literature records*: Ceylon (Smith, F. 1858: 13), Kandy (Emery 1893a: 249), Ceylon (Forel 1895a; 466), Sri Lanka (Shattuck 1994: 68), Colombo (Dill 2002: 63), Sri Lanka (Dias 2014: 82), Colombo, Galle, Gampaha, Ratnapura (Dias and Rajapaksa 2016: 33); *Secondary literature records*: Ceylon (Emery 1913: 14), Ceylon (Wheeler 1919a: 99), Emery 1925: 271 (Ceylon), Ceylon (Menozzi 1932: 11), Ceylon (Chapman and Capco 1951: 200), Sri Lanka (Dias 2002: 19), Sri Lanka (Dias 2006a: 50), Sri Lanka (Dias et al. 2012: 17); *AntWeb records*: Sri Lanka: CASENT 0902971 (AntWeb 2020).

***Dolichoderus taprobanae gracilipes* (Mayr, 1879)**

*Hypoclinea gracilipes* Mayr, 1879: 658. TL: [Calcutta] Kolkata, West Bengal: India [Syntype: NHMW]. [Images of CASENT 0915558 syntype worker examined].

**Distribution.***Primary literature records*: Ceylon (Forel 1895a: 466); *Secondary literature records*: Ceylon (Bingham 1903: 296), Sri Lanka (Dill 2002: 64).

***Dolichoderus thoracicus* (Smith F, 1860)**

*Tapinoma thoracica* Smith F, 1860b: 69. TL: [Mak.] Makassar, Sulawesi Selatan: Indonesia [Syntype: OUMNH]. [Images of CASENT 0901926 syntype worker examined].

**Distribution.** Wet Zone; *Primary literature records*: Ceylon (Wheeler 1942: 212), Colombo, Galle, Gampaha, Ratnapura (Dias and Rajapaksa 2016: 33); *Secondary literature records*: Sri Lanka (Dill 2002: 65).

#### *Iridomyrmex*: 1 species

***Iridomyrmex anceps* (Roger, 1863)**

*Formica anceps* Roger, 1863: 164. TL: [Malakka], Selangor: Malaysia [Syntypes: ZMHB]. [Images of FOCOL 2796–2797 syntype workers examined].

**Distribution.***Primary literature records*: Ceylon (Forel 1895a: 469); *Secondary literature records*: Ceylon (Bingham 1903: 299), Sri Lanka (Tiwari et al. 1999: 246), Sri Lanka (Dias 2002: 19), Sri Lanka (Li-Zhong 2006: 267).

#### *Ochetellus*: 1 species

***Ochetellus glaber* (Mayr, 1862)**

*Hypoclinea glabra* Mayr, 1862: 705. TL: [Sidney] Sydney, New South Wales: Australia [Syntype: NHMW]. [Images of CASENT 0915587 syntype male examined].

**Distribution.***Primary literature records*: Sri Lanka (Dias 2006a: 50); *Secondary literature records*: Sri Lanka (Dias 2006b: 8), Sri Lanka (Dias et al. 2012: 17).

#### *Tapinoma*: 3 species

***Tapinoma annandalei* (Wheeler, 1928)**

*Zatapinoma annandalei* Wheeler, 1928: 20. TL: Barkuda Island, Chilka Lake, Madras: India [Syntypes: MCZ].

**Distribution.** Dry Zone; *AntWeb records*: Polonnaruwa, Mahawa: CASENT 0172851, 0172852, CASENT 0172855 (AntWeb 2020).

***Tapinoma indicum* Forel, 1895**

*Tapinoma indicum* Forel, 1895a: 472. TL: [Poona] Pune: India [Syntype: MHNG]. [Images of CASENT 0909774 syntype worker examined].

**Distribution.** Wet, Dry and Intermediate Zones; *Primary literature records*: Ceylon (Forel 1909: 395), Trincomalee (Forel 1913b: 663), Sri Lanka Cashew Corporation-East, eastern Sri Lanka (Rickson and Rickson 1998: 843), Ratnapura, Galle (Dias 2006a: 45), Dambulla (Dias and Kosgamage 2008: 115), Gilimale Forest Reserve (Dias and Perera 2011: 71), Anuradhapura, Polonnaruwa (Dias and Kosgamage 2012: 61), Kirikanda Forest (Dias et al. 2013: 64), Sri Lanka (Dias 2014: 83), Kuluna Kanda Proposed Forest Reserve (Dias and Ruchirani 2014: 88), Mawathagama, Kurunegala (Dias and Peiris 2015b: 25), Anuradhapura, Colombo, Galle, Gampaha, Kurunegala, Polonnaruwa, Puttalam, Ratnapura (Dias and Rajapaksa 2016: 33); *Secondary literature records*: Ceylon (Emery 1913: 41), Ceylon (Chapman and Capco 1951: 193), Sri Lanka (Shattuck 1994: 145), Sri Lanka (Dias 2006b: 8), Sri Lanka (Dias et al. 2012: 17).

***Tapinoma melanocephalum* (Fabricius, 1793)**

*Formica melanocephala* Fabricius, 1793: 353. TL: Cayenne, French Guiana [Type: UNK].

**Distribution.** Wet, Dry and Intermediate Zones; *Primary literature records*: Colombo (Emery 1893a: 249), Ceylon (Forel 1895a: 472), Ceylon (Emery 1901: 121), Peradeniya (Forel 1911a: 226), Kelaniya, Colombo, Ratnapura (Dias 2006a: 45), Dambulla (Dias and Kosgamage 2008: 115), Nawalapitiya (Amarasinghe 2010: 12), Gilimale Forest Reserve (Dias and Perera 2011: 71), Anuradhapura, Polonnaruwa (Dias and Kosgamage 2012: 61), Sri Lanka (Dias 2014: 82), Kuluna Kanda Proposed Forest Reserve (Dias and Ruchirani 2014: 88), Ihakuluweva (Dias and Peiris 2015a: 5), Anuradhapura, Colombo, Galle, Gampaha, Kurunegala, Polonnaruwa, Puttalam, Ratnapura (Dias and Rajapaksa 2016: 33), Meethirigala Forest Reserve (Dias and Udayakantha 2016a: 53); *Secondary literature records*: Sri Lanka (Dias 2002: 19), Sri Lanka (Dias 2006b: 8), Sri Lanka (Dias et al. 2012: 17); *AntWeb records*: Laxapathiya, nr. Moratuwa: CASENT 0172853, 0172854 (AntWeb 2020).

#### *Technomyrmex*: 5 species

***Technomyrmex albipes* (Smith F, 1861)**

*Formica albipes* Smith F, 1861: 38. TL: Tondano [Tond], Sulawesi Utara: Indonesia [Syntypes: OUMNH]. [Images of CASENT 0102952 syntype worker examined].

**Distribution.** Wet, Dry and Intermediate Zones; *Primary literature records*: Kandy, Kottawa, Matale, Nawalapitiya (Emery 1893a: 249), Ceylon (Forel 1895a; 467), Ceylon (Emery 1901: 121), Puwakpitiya (Forel 1908: 3), Ceylon (Forel 1909: 395), Peradeniya, Seenigoda (Forel 1911a: 226), Peradeniya (Forel 1913a: 94), Bandarawela, Nalanda (Forel 1913b: 663), Peradeniya (Karavaiev 1926: 441), Nalanda (Baroni Urbani 1977a: 87), Colombo, Bandarawela, Nalanda (Shattuck 1994: 157, 158), South Sri Lanka (Way and Bolton 1997: 443), Sri Lanka Cashew Corporation-East, eastern Sri Lanka, Sri Lanka Cashew Corporation-West, western Sri Lanka, Panadura, Pushparanghnam Estate (Rickson and Rickson 1998: 843), Pattiyagedara, Sitrakala, Kandy, Hantana, Kataluoya Estate, Nuwara Eliya, Horton Plains N.P., Anuradhapura Dist., Maha Illupullansa Res. Farm, Prov. Uva, Egodapitiya Nilgala, Rat. Dist., Induruwa Jungle, Gilimale, Colombo, nr Kandy, Bandarawela, Nalanda (Bolton 2007: 71), Dambulla (Dias and Kosgamage 2008: 115), Sinharaja Forest Reserve (Gunawardene et al. 2008: 79), Nawalapitiya (Amarasinghe 2010: 12), Gilimale Forest Reserve (Dias and Perera 2011: 71), Anuradhapura, Polonnaruwa (Dias and Kosgamage 2012: 61), Sinharaja Forest Reserve (Gunawardene et al. 2012: 84), Kirikanda Forest (Dias et al. 2013: 64), Sri Lanka (Dias 2014: 85), Kuluna Kanda Proposed Forest Reserve, Wilpita “Aranya Kele” (Dias and Ruchirani 2014: 88), Ihakuluweva, Pallama, Madurankuliya (Dias and Peiris 2015a: 5), Sinhapura, Polonnaruwa (Dias and Peiris 2015b: 25), Meethirigala Forest Reserve (Udayakantha and Dias 2015: 31), Anuradhapura, Colombo, Galle, Gampaha, Kurunegala, Polonnaruwa, Puttalam, Ratnapura (Dias and Rajapaksa 2016: 33), Meethirigala Forest Reserve (Dias and Udayakantha 2016a: 54), Indikada Mukalana Forest Reserve (Dias and Udayakantha 2016b: 5), Indikada Mukalana Forest Reserve (Udayakantha and Dias 2018: 72); *Secondary literature records*: Ceylon (Viehmeyer 1912: 22), Ceylon (Emery 1922: 158), Ceylon (Chapman and Capco 1951: 195), Sri Lanka (Dias 2006a: 50), Sri Lanka (Dias 2006b: 8), Sri Lanka (Dias et al. 2012: 17); *AntWeb records*: Bandarawela, Nalanda, Induruwa Jungle, Gilimale: ANIC 32-011779, CASENT 0909789, CASENT 0909792, CASENT 0915552, FOCOL 0181–0182 (AntWeb 2020).

***Technomyrmex bicolor* Emery, 1893**

*Technomyrmex bicolor* Emery, 1893a: 249. TL: Kandy: [Ceylon] Sri Lanka [Syntype: MSNG]. [Images of CASENT 0905071 syntype worker examined].

**Distribution.** Wet, Dry and Intermediate Zones; *Primary literature records*: Kandy (Emery 1893a: 250), Ceylon (Forel 1895a: 467), Ceylon (Forel 1909: 395), Kelaniya (Dias 2006a: 45), Kandy (Bolton 2007: 72), Sinharaja Forest Reserve (Gunawardene et al. 2008: 79), Gilimale Forest Reserve (Dias and Perera 2011: 71), Anuradhapura, Polonnaruwa (Dias and Kosgamage 2012: 61), Sinharaja Forest Reserve (Gunawardene et al. 2012: 84), Sri Lanka (Dias 2014: 87), Ihakuluweva, Pallama, Egodayagama (Dias and Peiris 2015a: 5), Anuradhapura, Colombo, Galle, Gampaha, Kurunegala, Polonnaruwa, Puttalam, Ratnapura (Dias and Rajapaksa 2016: 33), Meethirigala Forest Reserve (Dias and Udayakantha 2016a: 53), Indikada Mukalana Forest Reserve (Dias and Udayakantha 2016b: 5), Indikada Mukalana Forest Reserve (Udayakantha and Dias 2018: 72); *Secondary literature records*: Ceylon (Emery 1913: 44), Kandy (Shattuck 1994: 159), Sri Lanka (Dias 2002: 19), Sri Lanka (Tiwari et al. 2004: 621), Sri Lanka (Dias 2006b: 8), Sri Lanka (Li-Zhong 2006: 272), Sri Lanka (Dias et al. 2012: 17); *AntWeb records*: Kandy: CASENT 0905071 (AntWeb 2020).

***Technomyrmex brunneus* Forel, 1895**

Technomyrmex albipes r. brunneus Forel, 1895a: 467. TL: [Poona] Pune: India [Syntype: MHNG]. [Images of CASENT 0909791 syntype worker examined].

**Distribution.** Wet and Dry Zones; *Primary literature records*: Kandy (Bolton 2007: 74), Sri Lanka (Dias 2014: 88), Puttalam (Dias and Rajapaksa 2016: 33); *Secondary literature records*: Sri Lanka (Le et al. 2010: 4).

***Technomyrmex elatior* Forel, 1902**

Technomyrmex modiglianii r. elatior Forel, 1902a: 293. TL: Assam: India [Syntypes: MHNG]. [Images of CASENT 0909804 syntype worker examined].

**Distribution.** Wet Zone; *Primary literature records*: Puwakpitiya (Forel 1908: 3), Ceylon (Forel 1909: 395), Kelaniya (Dias 2006a: 45); Sri Lanka (Dias 2014: 89), Colombo, Galle, Gampaha, Ratnapura (Dias and Rajapaksa 2016: 33); *Secondary literature records*: Ceylon (Emery 1913: 44), Ceylon (Chapman and Capco 1951: 196), Sri Lanka (Tiwari et al. 2004: 621), Sri Lanka (Dias 2006b: 8), Sri Lanka (Li-Zhong 2006: 272), Sri Lanka (Dias et al. 2012: 17).

***Technomyrmex horni* Forel, 1912**

*Technomyrmex horni* Forel, 1912b: 71. TL: Pilam [Formosa]: Taiwan [Syntypes: DEIC, MHNG]. [Images of CASENT 0909799, FOCOL 0169, 0170, FOCOL 0183 syntype workers and queen examined].

**Distribution.** Wet Zone; *Primary literature records*: Western Prov., Gampaha District, Pilikuttuwa (Bolton 2007: 85), Sri Lanka (Dias 2014: 90).

### DORYLINAE

#### *Aenictus*: 7 species

***Aenictus aitkenii* Forel, 1901**

*Aenictus aitkenii* Forel, 1901a: 475. TL: [Kanara], Karnataka India [Syntype: MHNG]. [Images of CASENT 0905981 syntype worker examined].

**Distribution.** Wet Zone; *Primary literature records*: Ceylon (Forel 1911b: 453), Peradeniya (Wilson 1964: 447), Udngama (Baroni Urbani 1977a: 65); *Secondary literature records*: Ceylon (Chapman and Capco 1951: 10), Sri Lanka (Shattuck 2008: 16); *AntWeb records*: Peradenyia: CASENT 0905986 (AntWeb 2020).

***Aenictus biroi* Forel, 1907**

*Aenictus biroi* Forel, 1907a: 10. TL: Pattipola: [Ceylon] Sri Lanka [Lectotype: MHNG]. [Images of CASENT 0905989 lectotype worker examined].

**Distribution.** Wet Zone; *Primary literature records*: Pattipola (Forel 1907a: 11), Pattipola (Wilson 1964: 451), Ceylon (Baroni Urbani 1977a: 65), Pattipola (Jaitrong et al. 2010: 37), Sri Lanka (Dias 2014: 43); *Secondary literature records*: Ceylon (Emery 1910: 29), Sri Lanka (Dias 2002: 17), Sri Lanka (Dias 2006a: 50), Sri Lanka (Dias et al. 2012: 15), Sri Lanka (Borowiec 2016: 85); *AntWeb records*: Pattipola: CASENT 0905989, CASENT 0922454 (AntWeb 2020).

***Aenictus ceylonicus* (Mayr, 1866)**

*Typhlatta ceylonica* Mayr, 1866: 505. TL: [Ceylon] Sri Lanka [Syntype: NHMW].

**Distribution.***Primary literature records*: Ceylon (Mayr 1866: 505), Ceylon (Forel 1901a: 477), Ceylon (Wilson 1964: 453), Sri Lanka (Jaitrong and Yamane 2013: 220), Sri Lanka (Dias 2014: 44); *Secondary literature records*: Ceylon (Emery 1910: 29), Ceylon (Chapman and Capco 1951: 11), Ceylon (Taylor 1987: 6), Sri Lanka (Ali 1991: 6), Sri Lanka (Tiwari 1999: 17), Sri Lanka (Dias 2002: 17), Sri Lanka (Dias 2006a: 50), Sri Lanka (Li-Zhong 2006: 263), Sri Lanka (Shattuck 2008: 16), Sri Lanka (Sheela 2008a: 6), Sri Lanka (Dias et al. 2012: 15), Sri Lanka (Borowiec 2016: 86).

***Aenictus fergusoni* Forel, 1901**

*Aenictus fergusoni* Forel, 1901a: 473. TL: Travancore: India [Syntypes: MHNG, SIZK, ZMHB]. [Images of CASENT 0905998, CASENT 0917746, FOCOL 1148 syntype workers examined].

**Distribution.** Wet and Dry Zones; *Primary literature records*: Sri Lanka (Dias 2002: 17), Mihintale Forest (Dias and Kosgamage 2012: 61), Sri Lanka (Dias 2014: 46), Anuradhapura, Colombo, Galle, Gampaha, Polonnaruwa, Ratnapura (Dias and Rajapaksa 2016: 33); *Secondary literature records*: Sri Lanka (Dias 2006a: 50), Sri Lanka (Dias 2006b: 8), Sri Lanka (Dias et al. 2012: 15).

***Aenictus gracilis* Emery, 1893**

*Aenictus gracilis* Emery, 1893c: 187. TL: Sarawak: Malaysia [Lectotype: MSNG]. [Images of CASENT 0903747 paralectotype worker examined].

**Distribution.** Wet Zone; *Primary literature records*: Ramboda (Karavaiev 1926: 424), Ramboda (Wilson 1964: 463); *Secondary literature records*: Ceylon (Chapman and Capco 1951: 13), Ceylon (Terayama and Yamane 1989: 599); *AntWeb records*: Ramboda: CASENT 0916860 (AntWeb 2020).

***Aenictus pachycerus* (Smith F, 1858)**

*Eciton pachycerus* Smith F, 1858: 153. TL: India [Syntype: NHMUK]. [Images of CASENT 0902674 syntype worker examined].

**Distribution.** Wet and Dry Zones; *Primary literature records*: Ceylon (Forel 1901a: 476), Ceylon (Wilson 1964: 471), Mihintale Forest (Dias and Kosgamage 2012: 61), Sri Lanka (Dias 2014: 47), Anuradhapura, Colombo, Galle, Gampaha, Polonnaruwa, Ratnapura (Dias and Rajapaksa 2016: 33); *Secondary literature records*: Ceylon (Emery 1910: 30), Ceylon (Chapman and Capco 1951: 10), Sri Lanka (Dias 2002: 17), Sri Lanka (Bharti 2003: 718), Sri Lanka (Dias 2006a: 50), Sri Lanka (Dias et al. 2012: 15).

***Aenictus porizonoides* Walker, 1860**

*Aenictus porizonoides* Walker, 1860: 306. TL: [Ceylon] Sri Lanka [Holotype: NHMUK]. [Images of CASENT 0902689 holotype male examined].

**Distribution.** Wet Zone; *Primary literature records*: Ceylon (Walker 1860: 306); Sri Lanka (Dias 2014: 49); *Secondary literature records*: Ceylon (Emery 1910: 30), Ceylon (Chapman and Capco 1951: 15), Sri Lanka (Dias 2002: 17), Sri Lanka (Dias 2006a: 50), Sri Lanka (Dias et al. 2012: 15), Sri Lanka (Borowiec 2016: 89); *AntWeb records*: Colombo: CASENT 0902690 (AntWeb 2020).

#### *Dorylus*: 2 species

***Dorylus labiatus* Shuckard, 1840**

*Dorylus labiatus* Shuckard, 1840: 319. TL: India [Syntype: OUMNH]. [Images of CASENT 0901950 syntype male examined].

**Distribution.** Wet and Dry Zones; *Primary literature records*: Sri Lanka (Dias et al. 2012: 17), Sri Lanka (Dias 2014: 93), Anuradhapura, Colombo, Galle, Gampaha, Polonnaruwa, Ratnapura (Dias and Rajapaksa 2016: 34).

***Dorylus orientalis* Westwood, 1835**

*Dorylus orientalis* Westwood, 1835: 72. TL: [East India]: India [Syntype: OUMNH]. [Images of CASENT 0901942 syntype male examined].

**Distribution.** Wet and Dry Zones; *Primary literature records*: Ceylon (Emery 1895a: 731), Ceylon (Emery 1901: 113), Ceylon (Forel 1901a: 464), Trincomalee, Ambalangoda (Forel 1909: 393), Peradeniya (Forel 1913a: 20), Peradeniya (Karavaiev 1926: 422), Kandy (Wilson 1964: 442), Gampaha (Dias 2006a: 45), Minneriya (Dias and Kosgamage 2012: 61), Sri Lanka (Dias 2014: 96), Anuradhapura, Colombo, Galle, Gampaha, Polonnaruwa, Ratnapura (Dias and Rajapaksa 2016: 34); *Secondary literature records*: Ceylon (Emery 1910: 15), Ceylon (Chapman and Capco 1951: 9), Sri Lanka (Roonwal 1976: 309), Sri Lanka (Ali 1991: 6), Sri Lanka (Tiwari 1997: 443), Sri Lanka (Tiwari 1999: 16), Sri Lanka (Tiwari et al. 1999: 228), Sri Lanka (Mathew and Tiwari 2000: 268), Sri Lanka (Dias 2002: 17), Sri Lanka (Tiwari and Tiwari 2002: 151), Sri Lanka (Tiwari et al. 2003: 472), Sri Lanka (Tak and Rathore 2004a: 166), Sri Lanka (Tak and Rathore 2004b: 85), Sri Lanka (Dias 2006b: 8), Sri Lanka (Rajan et al. 2006: 169), Sri Lanka (Tak et al. 2007: 128), Sri Lanka (Sheela 2008a: 9), Sri Lanka (Tak 2008: 9), Sri Lanka (Tak and Kazmi 2011: 41), Sri Lanka (Dias et al. 2012: 17).

#### *Lioponera*: 2 species

***Lioponera longitarsus* Mayr, 1879**

*Lioponera longitarsus* Mayr, 1879: 667. TL: [Ostind.]: India [Syntype: NHMW]. [Images of CASENT 0901942 syntype worker examined].

**Distribution.** Intermediate Zone; *Material examined*: 3 workers [ZEMK], Kurunegala District, Egodayagama, Kumbukweva Forest, 07°47'N, 80°12'E, 06.ii.2010 (leg. H.A.W.S. Peiris). First country record from Sri Lanka.

***Lioponera parva* Forel, 1900**

Lioponera longitarsus r. parva Forel, 1900b: 330. [Calcutta] Kolkata, West Bengal: India [Syntype: MHNG]. [Images of CASENT 0907070 syntype worker examined].

**Distribution.** Ceylon (Forel 1909: 393)

#### *Ooceraea*: 4 species

***Ooceraea alii* (Bharti & Akbar, 2013)**

*Cerapachys alii* Bharti & Akbar, 2013: 86. TL: Salim Ali Bird Sanctuary, Kerala: India [Holotype: PUAC]. [Holotype worker examined].

**Distribution.** Dry Zone; *Primary literature records*: Ihakuluwewa (Dias et al. 2018: 450).

***Ooceraea biroi* (Forel, 1907)**

*Cerapachys biroi* Forel, 1907a: 7. TL: Singapore [Lectotype: MHNG]. [Images of CASENT 0907059 paralectotype worker examined].

**Distribution.** Wet Zone; *Primary literature records*: Watinapaha (Dias et al. 2018: 451), Indikada Mukalana Forest Reserve (Udayakantha and Dias 2018: 72).

***Ooceraea coeca* Mayr, 1897**

*Ooceraea coeca* Mayr, 1897: 420. TL: Kalawewa: [Ceylon] Sri Lanka [Syntype: HNHM]. [Images of CASENT 0922424 syntype worker examined].

**Distribution.** Dry Zone; *Primary literature records*: Kalawewa (Mayr 1897: 421), Ceylon (Forel 1900b: 329), Kandy, Kantale (Brown 1975: 74); *Secondary literature records*: Ceylon (Emery 1911: 10), Ceylon (Chapman and Capco 1951: 20), Sri Lanka (Dias 2002: 17), Sri Lanka (Dias 2006a: 50), Sri Lanka (Dias et al. 2012: 17), Sri Lanka (Borowiec 2016: 198); *AntWeb records*: Kalawewa: CASENT 0922424 (AntWeb 2020).

***Ooceraea fragosa* Roger, 1862**

*Ooceraea fragosa* Roger, 1862: 249. TL: [Ceylon] Sri Lanka [Syntype: ZMHB]. [Images of FOCOL 0802 syntype worker examined].

**Distribution.** Wet, Dry and Intermediate Zones; *Primary literature records*: Ceylon (Roger 1862: 249), Ceylon (Forel 1900b: 329), Kantale, Yakkala (Brown 1975: 74), Sinharaja Forest Reserve (Gunawardene et al. 2008: 79), Sri Lanka (Dias 2014: 75), Colombo, Galle, Gampaha, Kurunegala, Ratnapura (Dias and Rajapaksa 2016: 34); *Secondary literature records*: Ceylon (Emery 1911: 10), Ceylon (Chapman and Capco 1951: 20), Sri Lanka (Dias 2002: 17), Sri Lanka (Dias 2006a: 50), Sri Lanka (Dias et al. 2012: 17), Sri Lanka (Borowiec 2016: 198); *AntWeb records*: Sinharaja Forest Reserve: CASENT 0106215, FOCOL 0802 (AntWeb 2020).

#### *Parasyscia*: 3 species

***Parasyscia aitkenii* (Forel, 1900)**

*Cerapachys aitkenii* Forel, 1900b: 332. TL: [Kanara], Karnataka: India [Syntypes: MHNG]. [Images of CASENT 0907048 syntype worker examined].

**Distribution.** Intermediate Zone; *Primary literature records*: Sri Lanka (Dias et al. 2012: 17), Sri Lanka (Dias 2014: 74), Egodayagama (Dias and Peiris 2015a: 5), Kurunegala (Dias and Rajapaksa 2016: 34).

***Parasyscia fossulata* (Forel, 1895)**

*Cerapachys fossulatus* Forel, 1895b: 48. TL: [Ceylon] Sri Lanka [Syntype: ZMHB]. [Images of FOCOL 0797 syntype worker examined].

**Distribution.** Wet and Intermediate Zones; *Primary literature records*: Ceylon (Forel 1895b: 49), Ceylon (Forel 1900b: 332), Ceylon (Brown 1975: 22), Sri Lanka (Dias 2014: 75), Colombo, Galle, Gampaha, Kurunegala, Ratnapura (Dias and Rajapaksa 2016: 34); *Secondary literature records*: Ceylon (Emery 1911: 9), Ceylon (Chapman and Capco 1951: 20), Sri Lanka (Dias 2002: 17), Sri Lanka (Dias 2006a: 50), Sri Lanka (Dias et al. 2012: 17), Sri Lanka (Borowiec 2016: 204); *AntWeb records*: Sri Lanka: FOCOL 0797 (AntWeb 2020).

***Parasyscia luteoviger* (Brown, 1975)**

*Cerapachys luteoviger* Brown, 1975: 70. TL: Gilimale, near Ratnapura: Sri Lanka [Paratype: NHMUK]. [Images of CASENT 0902744 paratype worker examined].

**Distribution.** Wet Zone; *Primary literature records*: Gilimale, Ratnapura (Brown 1975: 70), Sinharaja Forest Reserve (Gunawardene et al. 2012: 84); *Secondary literature records*: Sri Lanka (Dias 2006a: 50), Sri Lanka (Dias et al. 2012: 17), Sri Lanka (Borowiec 2016: 205); *AntWeb records*: Gilimale: CASENT 0902744 (AntWeb 2020).

#### *Syscia*: 1 species

***Syscia typhla* Roger, 1861**

*Syscia typhla* Roger, 1861: 20. TL: [Ceylon] Sri Lanka [Syntype: ZMHB]. [Images of FOCOL 0804 syntype worker examined].

**Distribution.** Wet, Dry and Intermediate Zones; *Primary literature records*: Ceylon (Roger 1861: 21), Ceylon (Forel 1900b: 329), Ceylon (Brown 1975: 24), Sinharaja Forest Reserve (Gunawardene et al. 2008: 79), Sinharaja Forest Reserve (Gunawardene et al. 2012: 84), Sri Lanka (Dias 2014: 76), Ihakuluweva (Dias and Peiris 2015a: 5), Anuradhapura, Kurunegala, Polonnaruwa (Dias and Rajapaksa 2016: 34); *Secondary literature records*: Ceylon (Emery 1911: 10), Ceylon (Chapman and Capco 1951: 20), Sri Lanka (Dias 2002: 17), Sri Lanka (Dias 2006a: 50), Sri Lanka (Dias et al. 2012: 17), Sri Lanka (Borowiec 2016: 224); *AntWeb records*: Sinharaja Forest Reserve: CASENT 0106214, FOCOL 0804 (AntWeb 2020).

### ECTATOMMINAE

#### *Gnamptogenys*: 2 species

***Gnamptogenys coxalis* (Smith F, 1857)**

*Ponera rugosa* Smith F, 1857: 66. TL: Borneo, Sarawak: Malaysia [Holotype: OXUM]. [Images of CASENT 0901369 holotype queen examined].

**Distribution.***AntWeb records*: Sri Lanka: CASENT 0281842 (AntWeb 2020).

***Gnamptogenys sinhala* Lattke, 2016**

*Gnamptogenys sinhala* Lattke, 2016: 146. TL: [Ceylon] Sri Lanka [Holotype: NHMW]. [Images of CASENT 0915911 holotype worker examined].

**Distribution.***Primary literature records*: Ceylon (Roger 1860: 309), Ceylon (Forel 1900b: 316), Ceylon (Brown 1954: 9), Ceylon (Brown 1958: 228), Sri Lanka (Lattke 2004: 103), Sri Lanka (Lattke and Delsinne 2016: 146); *Secondary literature records*: Ceylon (Emery 1911: 48), Ceylon (Wheeler 1919a: 51), Ceylon (Chapman and Capco 1951: 29), Sri Lanka (Ali 1991: 3), Sri Lanka (Dias 2002: 19), Sri Lanka (Dias 2006a: 52), Sri Lanka (Dias et al. 2012: 17), Sri Lanka (Dias 2014: 101); *AntWeb records*: Sri Lanka: CASENT 0915911, FOCOL 0873–0884 (AntWeb 2020).

### FORMICINAE

#### *Acropyga*: 2 species

***Acropyga acutiventris* Roger, 1862**

*Acropyga acutiventris* Roger, 1862: 243. TL: [Ceylon] Sri Lanka [Syntypes: ZMHB]. [Images of FOCOL 2238–2240 syntype worker and queens examined].

**Distribution.** Wet and Dry Zones; *Primary literature records*: Ceylon (Roger 1862: 244), Ceylon (Forel 1894: 418), Ceylon (Emery 1901: 121), Ceylon (Forel 1909: 395), Weligama (Forel 1913b: 663), Botanical Garden, Peradeniya (Lapolla 2004: 33), Sinharaja Forest Reserve (Gunawardene et al. 2008: 80), Gilimale Forest Reserve (Dias and Perera 2011: 71); Anuradhapura, Polonnaruwa (Dias and Kosgamage 2012: 61), Sri Lanka (Dias 2014: 108), Ihakuluwewa (Dias and Peiris 2015a: 5), Anuradhapura, Colombo, Galle, Gampaha, Polonnaruwa, Ratnapura (Dias and Rajapaksa 2016: 34); *Secondary literature records*: Ceylon (Emery 1925: 28), Ceylon (Chapman and Capco 1951: 210), Sri Lanka (Ali 1992: 7), Sri Lanka (Taylor 1992: 58), Sri Lanka (Dias 2002: 18), Sri Lanka (Terayama et al. 2002: 23), Sri Lanka (Dias et al. 2012: 18), Sri Lanka (Jaitrong and Nabhitabhata 2005: 10), Sri Lanka (Dias 2006b: 8); *AntWeb records*: Sri Lanka: FOCOL 2238–2240 (AntWeb 2020).

***Acropyga rubescens* Forel, 1894**

Acropyga acutiventris var. rubescens Forel, 1894: 418. TL: [Belgam] Belgaum: India [Syntypes: HNHM, MHNG, NHMUK]. [Images of CASENT 0916667, CASENT 0903175, CASENT 0909898 syntype workers examined].

**Distribution.** Wet Zone; *Primary literature records*: Peradeniya (Forel 1911a: 227); Ratnapura, Kandy (Lapolla 2004: 34); *Secondary literature records*: Ceylon (Emery 1925: 28), Ceylon (Chapman and Capco 1951: 211), Sri Lanka (Dias 2006a: 50); *AntWeb records*: Peradenyia: CASENT 0909900 (AntWeb 2020).

#### *Anoplolepis*: 1 species

***Anoplolepis gracilipes* (Smith F, 1857)**

*Formica gracilipes* Smith F, 1857: 55. TL: Singapore [Syntypes: NHMUK, OUMNH]. [Images of CASENT 0102951, CASENT 0103001, CASENT 0903237 syntype workers examined].

**Distribution.** Wet, Dry and Intermediate Zones; *Primary literature records*: Pointe de Galle (Emery 1887a: 247), Kandy, Galle, Matale (Emery 1893a: 253), Ceylon (Forel 1894: 415), Ceylon (Emery 1901: 121), Puwakpitiya, Galle (Forel 1908: 4), Peradeniya (Forel 1911a: 226), Peradeniya, Maha Iluppalama (Forel 1913a: 100), South Sri Lanka (Way and Bolton 1997: 443), Cashew Corporation-East, eastern Sri Lanka (Rickson and Rickson 1998: 843), Kelaniya, Gampaha, Ratnapura (Dias 2006a: 45), Dambulla (Dias and Kosgamage 2008: 115), Sinharaja Forest Reserve (Gunawardene et al. 2008: 80), Nawalapitiya (Amarasinghe 2010: 12), Gilimale Forest Reserve (Dias and Perera 2011: 71), Anuradhapura, Polonnaruwa (Dias and Kosgamage 2012: 61), Sri Lanka (Dias 2014: 109), Kuluna Kanda Proposed Forest Reserve, Wilpita “Aranya Kele” (Dias and Ruchirani 2014: 88), Namalweva, Ihakuluweva, Madurankuliya, Egodayagama (Dias and Peiris 2015a: 5), Mawathagama, Kurunegala (Dias and Peiris 2015b: 25), Anuradhapura, Colombo, Galle, Gampaha, Kurunegala, Polonnaruwa, Puttalam, Ratnapura (Dias and Rajapaksa 2016: 34); *Secondary literature records*: Sri Lanka (Mathew 1984: 308), Ceylon (Mukherji and Ribeiro 1925: 208), Sri Lanka (Ali 1992: 7), Sri Lanka (Tiwari et al. 1999: 279), Sri Lanka (Mathew 2000: 352), Sri Lanka (Mathew and Tiwari 2000: 348), Sri Lanka (Dias 2002: 18), Sri Lanka (Tiwari et al. 2003: 496), Sri Lanka (Tiwari et al. 2004: 620), Sri Lanka (Wetterer 2005: 4), Sri Lanka (Dias 2006b: 8), Sri Lanka (Dias et al. 2012: 18); *AntWeb records*: Dunhinda Falls, Uda Walawe, Peradenyia: ANIC 32-012852–32-012856; CASENT 0246608 (AntWeb 2020).

#### *Camponotus*: 41 species/subspecies

***Camponotus albipes* Emery, 1893**

*Camponotus albipes* Emery, 1893a: 253. TL: Kandy: [Ceylon] Sri Lanka [Syntype: MSNG]. [Images of CASENT 0905459 syntype worker examined].

**Distribution.** Wet Zone; *Primary literature records*: Kandy (Emery 1893a: 253); *Secondary literature records*: Ceylon (Emery 1896: 772), Ceylon (Emery 1925: 138), Ceylon (Chapman and Capco 1951: 230), Sri Lanka (Dias 2002: 18), Sri Lanka (Dias 2006a: 50), Sri Lanka (Dias et al. 2012: 18); *AntWeb records*: Kandy: CASENT 0905459 (AntWeb 2020).

***Camponotus angusticollis* (Jerdon, 1851)**

*Formica angusticollis* Jerdon, 1851: 120. TL: Malabar, Kerala: India [Type: UNK].

**Distribution.** Wet Zone; *Primary literature records*: Kottawa (Emery 1893a: 250), Puwakpitiya (Forel 1908: 8), Ceylon (Forel 1912c: 67).

***Camponotus auriculatus* Mayr, 1897**

*Camponotus auriculatus* Mayr, 1897: 432. TL: [Ceylon] Sri Lanka [Syntypes: HNHM, NHMW]. [Images of CASENT 0915603, CASENT 0922416 syntype workers examined].

**Distribution.***Primary literature records*: Ceylon (Mayr 1897: 435); *Secondary literature records*: Ceylon (Forel 1903a: 711), Ceylon (Emery 1925: 138), Ceylon (Chapman and Capco 1951: 230), Sri Lanka (Dias 2002: 18), Sri Lanka (Dias 2006a: 50), Sri Lanka (Dias et al. 2012: 18); *AntWeb records*: Sri Lanka: CASENT 0915603, CASENT 0922416 (AntWeb 2020).

***Camponotus barbatus* Roger, 1863**

*Camponotus barbatus* Roger, 1863: 138. TL: [Ceylon] Sri Lanka [Syntypes: ZMHB]. [Images of FOCOL 2427, 2428 syntype worker and queen examined].

**Distribution.** Wet Zone; *Primary literature records*: Ceylon (Roger 1863: 138), Ceylon (Forel 1892a: 233), Bandarawela (Emery 1901: 121); *Secondary literature records*: Ceylon (Emery 1896: 769), Ceylon (Forel 1903a: 711), Ceylon (Emery 1925: 92), Ceylon (Chapman and Capco 1951: 243), Sri Lanka (Tiwari 1999: 71), Sri Lanka (Dias 2002: 18), Sri Lanka (Dias 2006a: 50), Sri Lanka (Dias et al. 2012: 18); *AntWeb records*: Sri Lanka: FOCOL 2427, 2428 (AntWeb 2020).

***Camponotus barbatus infuscoides* Bingham, 1903**

Camponotus taylori var. infuscoides Bingham, 1903: 354. TL: [Ceylon] Sri Lanka [Syntype: MHNG]. [Images of CASENT 0910135 syntype worker examined].

**Distribution.***Primary literature records*: Ceylon (Bingham 1903: 354), Ceylon (Forel 1909: 397); *Secondary literature records*: Ceylon (Emery 1925: 93), Ceylon (Chapman and Capco 1951: 243); *AntWeb records*: Sri Lanka: CASENT 0910135 (AntWeb 2020).

***Camponotus barbatus taylori* Forel, 1892**

Camponotus maculatus r. taylori Forel, 1892a: 241. TL: [Orissa] Odisha: India [Syntypes: MHNG]. [Images of CASENT 0910133, 0910134 syntype workers examined].

**Distribution.** Wet Zone; *Primary literature records*: Ceylon (Forel 1903a: 711); Paradeniya (Forel 1907b: 19); *Secondary literature records*: Ceylon (Emery 1925: 93), Ceylon (Chapman and Capco 1951: 243), Sri Lanka (Tiwari 1999: 71), Sri Lanka (Tiwari et al. 1999: 281), Sri Lanka (Tiwari et al. 2003: 502), Sri Lanka (Tak 2008: 36), Sri Lanka (Tak 2010: 140); *AntWeb records*: Paradeniya: CASENT 0910132, CASENT 0917853 (AntWeb 2020).

***Camponotus carin* Emery, 1889**

Camponotus dorycus r. carin Emery, 1889: 512. TL: Tenasserim, M. Mooleyit: Myanmar [Syntypes: MSNG]. [Images of CASENT 0905251, 0905252 syntype workers examined].

**Distribution.***Primary literature records*: Ceylon (Forel 1892a: 236).

***Camponotus compressus* (Fabricius, 1787)**

*Formica compressa* Fabricius, 1787: 307. TL: Tharangambadi, Tamil Nadu: India [Type: UNK].

**Distribution.** Wet, Dry and Intermediate Zones; *Primary literature records*: Ceylon (Forel 1892a: 241), Ceylon (Emery 1901: 121), Ceylon (Forel 1909: 396), Anuradhapura, Polonnaruwa (Dias and Kosgamage 2012: 61), Sri Lanka (Dias 2014: 112), Namalweva, Ihakuluweva, Marawila, Pallama, Madurankuliya (Dias and Peiris 2015a: 5), Mawathagama, Kurunegala (Dias and Peiris 2015b: 25), Anuradhapura, Colombo, Galle, Gampaha, Kurunegala, Polonnaruwa, Puttalam, Ratnapura (Dias and Rajapaksa 2016: 34), Indikada Mukalana Forest Reserve (Udayakantha and Dias 2018: 72); *Secondary literature records*: Ceylon (Emery 1925: 98), Ceylon (Donisthorpe 1942b: 458), Sri Lanka (Ali 1992: 7), Sri Lanka (Tiwari 1997: 449), Sri Lanka (Tiwari 1999: 69), Sri Lanka (Mathew 2000: 352), Sri Lanka (Mathew and Tiwari 2000: 354), Sri Lanka (Dias 2002: 18), Sri Lanka (Tiwari and Tiwari 2002: 160), Sri Lanka (Tiwari et al. 2003: 500), Sri Lanka (Tak and Rathore 2004a: 168), Sri Lanka (Tak and Rathore 2004b: 86), Sri Lanka (Tiwari et al. 2004: 616), Sri Lanka (Ghosh et al. 2006: 378), Sri Lanka (Li-Zhong 2006: 264), Sri Lanka (Rajan et al. 2006: 169), Sri Lanka (Tak et al. 2007: 129), Sri Lanka (Sheela 2008a: 11), Sri Lanka (Tak 2008: 34), Sri Lanka (Tak 2010: 140), Sri Lanka (Tak and Kazmi 2011: 46), Sri Lanka (Dias et al. 2012: 18); *AntWeb records*: Jaffna: SAM-HYM-C 001820 (AntWeb 2020).

***Camponotus fletcheri* Donisthorpe, 1942**

*Camponotus fletcheri* Donisthorpe, 1942c: 250. TL: Humbantota: Sri Lanka [Holotype: NHMUK]. [Images of CASENT 0903578 holotype worker examined].

**Distribution.** Dry Zone; *Primary literature records*: Hambantota (Donisthorpe 1942c: 251); *Secondary literature records*: Ceylon (Chapman and Capco 1951: 241), Sri Lanka (Dias 2002: 18), Sri Lanka (Dias 2006a: 50), Sri Lanka (Dias et al. 2012: 18); *AntWeb records*: Hambantota: CASENT 0903578 (AntWeb 2020).

***Camponotus greeni* Forel, 1911**

*Camponotus greeni* Forel, 1911c: 54. TL: Namunukula: [Ceylon] Sri Lanka [Syntypes: MHNG]. [Images of CASENT 0910540, 0910541 syntype workers examined].

**Distribution.** Intermediate Zone; *Primary literature records*: Namunukula (Forel 1911c: 55); *Secondary literature records*: Ceylon (Emery 1925: 139), Ceylon (Chapman and Capco 1951: 231), Sri Lanka (Dias 2002: 18), Sri Lanka (Dias 2006a: 50), Sri Lanka (Dias et al. 2012: 18); *AntWeb records*: Namunukula: CASENT 0910540, 0910541 (AntWeb 2020).

***Camponotus indeflexus* (Walker, 1859)**

*Formica indeflexa* Walker, 1859: 373. TL: [Ceylon] Sri Lanka [Holotype: NHMUK]. [Images of CASENT 0903580 holotype worker examined].

**Distribution.***Primary literature records*: Ceylon (Walker 1859: 373); *Secondary literature records*: Emery 1925: 271 (Ceylon), Ceylon (Chapman and Capco 1951: 199), Sri Lanka (Dias 2002: 18), Sri Lanka (Dias 2006a: 50), Sri Lanka (Dias et al. 2012: 18); *AntWeb records*: Sri Lanka: CASENT 0903580 (AntWeb 2020).

***Camponotus irritans* (Smith F, 1857)**

*Formica irritans* Smith F, 1857: 55. TL: [Malac] Melaka: Malaysia [Syntype: OUMNH]. [Images of CASENT 0901899 syntype worker examined].

**Distribution.** Wet, Dry and Intermediate Zones; *Primary literature records*: Ceylon (Emery 1896: 769), Anuradhapura, Polonnaruwa (Dias and Kosgamage 2012: 61), Kirikanda Forest (Dias et al. 2013: 64), Sri Lanka (Dias 2014: 113), Ihakuluweva, Marawila, Madurankuliya, Egodayagama (Dias and Peiris 2015a: 5), Mawathagama, Kurunegala (Dias and Peiris 2015b: 25), Anuradhapura, Colombo, Galle, Gampaha, Kurunegala, Polonnaruwa, Puttalam, Ratnapura (Dias and Rajapaksa 2016: 34), Meethirigala Forest Reserve (Dias and Udayakantha 2016a: 53); *Secondary literature records*: Ceylon (Bingham 1903: 353), Ceylon (Emery 1925: 93), Ceylon (Chapman and Capco 1951: 246), Sri Lanka (Ali 1992: 8), Sri Lanka (Tiwari et al. 1999: 270), Sri Lanka (Dias 2002: 18), Sri Lanka (Tak and Rathore 2004a: 169), Sri Lanka (Tak and Rathore 2004b: 86), Sri Lanka (Dias 2006a: 50), Sri Lanka (Ghosh et al. 2006: 379), Sri Lanka (Rajan et al. 2006: 170), Sri Lanka (Tak 2008: 34), Sri Lanka (Dias et al. 2012: 18).

***Camponotus isabellae* Forel, 1909**

*Camponotus isabellae* Forel, 1909: 399. TL: [Ceylon] Sri Lanka [Syntypes: MHNG]. [Images of CASENT 0910539 syntype queen examined].

**Distribution.** Wet Zone; *Primary literature records*: Ambalangoda (Forel 1909: 401); *Secondary literature records*: Ceylon (Emery 1925: 139), Ceylon (Chapman and Capco 1951: 270), Sri Lanka (Dias 2002: 18), Sri Lanka (Dias 2006a: 50), Sri Lanka (Dias et al. 2012: 18); *AntWeb records*: Sri Lanka: CASENT 0910539 (AntWeb 2020).

***Camponotus latebrosus* (Walker, 1859)**

*Formica latebrosa* Walker, 1859: 371. TL: [Ceylon] Sri Lanka [Holotype: NHMUK]. [Images of CASENT 0903584 holotype male examined].

**Distribution.***Primary literature records*: Ceylon (Walker 1859: 371); *Secondary literature records*: Emery 1925: 271 (Ceylon), Ceylon (Chapman and Capco 1951: 200), Sri Lanka (Dias 2002: 18), Sri Lanka (Dias 2006a: 50), Sri Lanka (Dias et al. 2012: 18); *AntWeb records*: Sri Lanka: CASENT 0903584 (AntWeb 2020).

***Camponotus mendax* Forel, 1895**

Camponotus sericeus var. mendax Forel, 1895a: 454. TL: Mysore: India [Syntype: MHNG].

**Distribution.***Primary literature records*: Sri Lanka (Dias 2002: 18); *Secondary literature records*: Sri Lanka (Dias 2006a: 50), Sri Lanka (Dias et al. 2012: 18).

***Camponotus mendax integer* Forel, 1895**

Camponotus sericeus var. integer Forel, 1895a: 454. TL: [Ceylon] Sri Lanka [Syntype: MHNG]. [Images of CASENT 0910448 syntype worker examined].

**Distribution.** Wet Zone; *Primary literature records*: Ceylon (Forel 1895a: 455), Galle (Forel 1908: 6), Ceylon (Forel 1909: 397), Seenigoda (Forel 1911a: 227), Seenigoda, Peradeniya (Forel 1913a: 129); *Secondary literature records*: Ceylon (Emery 1896: 773), Ceylon (Chapman and Capco 1951: 241); *AntWeb records*: Sri Lanka: CASENT 0910448 (AntWeb 2020).

***Camponotus mitis* (Smith F, 1858)**

*Formica mitis* Smith F, 1858: 20. TL: [Ceylon] Sri Lanka [Syntype: NHMUK]. [Images of CASENT 0903590 syntype worker examined].

**Distribution.** Wet Zone; *Primary literature records*: Ceylon (Smith F, 1858: 20), Galle, Kandy, Colombo (Emery 1893a: 251), Pattipola, Puwakpitiya, Galle (Forel 1908: 6), Ceylon (Forel 1911a: 228), Peradeniya (Karavaiev 1929: 239); *Secondary literature records*: Ceylon (Emery 1925: 96), Ceylon (Chapman and Capco 1951: 252), Sri Lanka (Tiwari 1999: 72), Sri Lanka (Tiwari et al. 2003: 501), Sri Lanka (Dias 2006a: 50), Sri Lanka (Li-Zhong 2006: 264), Sri Lanka (Dias et al. 2012: 18); *AntWeb records*: Sri Lanka: CASENT 0903590 (AntWeb 2020).

***Camponotus oblongus* (Smith F, 1858)**

*Formica oblonga* Smith F, 1858: 21. TL: [Birmah] Myanmar [Holotype: NHMUK]. [Images of CASENT 0903585 holotype queen examined].

**Distribution.** Wet and Dry Zones; *Primary literature records*: Hakgala (Forel 1908: 6), Anuradhapura, Polonnaruwa (Dias and Kosgamage 2012: 61), Sri Lanka (Dias 2014: 113), Marawila (Dias and Peiris 2015a: 5), Anuradhapura, Polonnaruwa, Puttalam (Dias and Rajapaksa 2016: 34); *Secondary literature records*: Sri Lanka (Tiwari et al. 1999: 270), Sri Lanka (Tiwari et al. 2003: 501), Sri Lanka (Tiwari et al. 2004: 617), Sri Lanka (Dias et al. 2012: 18).

***Camponotus ominosus* Forel, 1911**

*Camponotus ominosus* Forel, 1911c: 52. TL: Namunukula: [Ceylon] Sri Lanka [Syntypes: MHNG]. [Images of CASENT 0910522, 0910523 syntype workers examined].

**Distribution.** Intermediate Zone; *Primary literature records*: Namunukula (Forel 1911c: 53); *Secondary literature records*: Ceylon (Emery 1925: 138), Ceylon (Chapman and Capco 1951: 233), Sri Lanka (Dias 2002: 18), Sri Lanka (Dias 2006a: 50), Sri Lanka (Dias et al. 2012: 18); *AntWeb records*: Namunukula: CASENT 0910522, 0910523 (AntWeb 2020).

***Camponotus opaciventris* Mayr, 1879**

*Camponotus opaciventris* Mayr, 1879: 648. TL: West Bengal: Kolkata: India [Syntypes: NHMW].

**Distribution.** Wet and Dry Zones; *Primary literature records*: Puwakpitiya, Peradeniya (Forel 1908: 6), Peradeniya (Forel 1911a: 227), Peradeniya, Maha Iluppalama (Forel 1913a: 129); *Secondary literature records*: Ceylon (Chapman and Capco 1951: 242).

***Camponotus parius* Emery, 1889**

Camponotus micans r. paria Emery, 1889: 513. TL: Yangon [Rangoon, Birmania]: Myanmar [Syntypes: MSNG]. [Images of CASENT 0905350, CASENT 0905805 syntype workers examined].

**Distribution.** Wet Zone; *Primary literature records*: Ceylon (Forel 1892a: 238), Ceylon (Emery 1901: 121), Puwakpitiya, Galle (Forel 1908: 6), Peradeniya (Forel 1911a: 227); *Secondary literature records*: Ceylon (Donisthorpe 1942b: 458), Sri Lanka (Ali 1992: 8), Sri Lanka (Tiwari 1999: 70), Sri Lanka (Tiwari et al. 1999: 280), Sri Lanka (Mathew and Tiwari 2000: 354), Sri Lanka (Dias 2002: 18), Sri Lanka (Rajan et al. 2006: 170); *AntWeb records*: Sri Lanka: CASENT 0905350 (AntWeb 2020).

***Camponotus reticulatus* Roger, 1863**

*Camponotus reticulatus* Roger, 1863: 139. TL: [Ceylon] Sri Lanka [Syntype: ZMHB?].

**Distribution.** Dry and Intermediate Zones; *Primary literature records*: Ceylon (Roger 1863: 139), Ceylon (Forel 1892a: 233), Anuradhapura, Polonnaruwa (Dias and Kosgamage 2012: 61), Namalweva, Marawila, Madurankuliya (Dias and Peiris 2015a: 5), Anuradhapura, Kurunegala, Polonnaruwa, Puttalam (Dias and Rajapaksa 2016: 34); *Secondary literature records*: Ceylon (Emery 1925: 139), Ceylon (Chapman and Capco 1951: 233), Sri Lanka (Dias 2002: 18), Sri Lanka (Dias 2006a: 50), Sri Lanka (Dias et al. 2012: 18).

***Camponotus reticulatus latitans* Forel, 1893**

Camponotus reticulatus var. latitans Forel, 1893b: 431. TL: [Ceylon] Sri Lanka [Syntypes: MHNG]. [Images of CASENT 0910528, 0910529 syntype workers examined].

**Distribution.** Wet Zone; *Primary literature records*: Ceylon (Forel 1893b: 431), Peradeniya (Forel 1911a: 228); *Secondary literature records*: Ceylon (Emery 1896: 772), Ceylon (Emery 1925: 139), Ceylon (Chapman and Capco 1951: 234); *AntWeb records*: Sri Lanka: CASENT 0910528, 0910529 (AntWeb 2020).

***Camponotus reticulatus yerburyi* Forel, 1893**

*Camponotus reticulatus yerburyi* Forel, 1893b: 431. TL: [Ceylon] Sri Lanka [Syntypes: MHNG]. [Images of CASENT 0910533, 0910534 syntype workers examined].

**Distribution.** Wet Zone; *Primary literature records*: Kottawa (Emery 1893a: 253), Ceylon (Forel 1893b: 432), Ceylon (Forel 1911a: 228); *Secondary literature records*: Ceylon (Emery 1896: 772), Ceylon (Emery 1925: 139), Ceylon (Chapman and Capco 1951: 234); *AntWeb records*: Sri Lanka: CASENT 0910533, 0910534 (AntWeb 2020).

***Camponotus rufoglaucus* (Jerdon, 1851)**

*Formica rufoglauca* Jerdon, 1851: 124. TL: Carnatic, Kerala: India [Type: UNK].

**Distribution.** Wet, Dry and Intermediate Zones; *Primary literature records*: Ceylon (Forel 1892a: 238), Kandy (Emery 1893a: 252), Puwakpitiya (Forel 1908: 6), Ceylon (Forel 1909: 397), Peradeniya (Forel 1911a: 227), Peradeniya (Forel 1913a: 129), Anuradhapura, Polonnaruwa (Dias and Kosgamage 2012: 61), Sri Lanka (Dias 2014: 114), Ihakuluweva, Marawila, Madurankuliya (Dias and Peiris 2015a: 5), Sinhapura, Polonnaruwa, Mawathagama, Kurunegala (Dias and Peiris 2015b: 26), Anuradhapura, Colombo, Galle, Gampaha, Kurunegala, Polonnaruwa, Puttalam, Ratnapura (Dias and Rajapaksa 2016: 34); *Secondary literature records*: Ceylon (Emery 1925: 105), Ceylon (Donisthorpe 1942b: 458), Ceylon (Chapman and Capco 1951: 238), Sri Lanka (Ali 1992: 8), Sri Lanka (Tiwari 1997: 450), Sri Lanka (Tiwari 1999: 70), Sri Lanka (Mathew and Tiwari 2000: 355), Sri Lanka (Dias 2002: 18), Sri Lanka (Tiwari and Tiwari 2002: 160), Sri Lanka (Dias 2006a: 50), Sri Lanka (Li-Zhong 2006: 264), Sri Lanka (Dias et al. 2012: 18).

***Camponotus sericeus* (Fabricius, 1798)**

*Formica sericea* Fabricius, 1798: 279. TL: Senegal [Type: UNK].

**Distribution.** Wet, Dry and Intermediate Zones; *Primary literature records*: Ceylon (Forel 1892a: 231), Kottawa, Colombo (Emery 1893a: 254), Ceylon (Emery 1901: 122), Ceylon (Forel 1908: 6), Peradeniya (Forel 1913a: 129), Peradeniya (Karavaiev 1929: 240), eastern Sri Lanka, Sri Lanka Cashew Corporation-West, western Sri Lanka, Panadura, Pushparanghnam Estate (Rickson and Rickson 1998: 843), Anuradhapura, Polonnaruwa (Dias and Kosgamage 2012: 61), Sri Lanka (Dias 2014: 115), Namalweva, Marawila, Pallama, Madurankuliya (Dias and Peiris 2015a: 5), Sinhapura, Polonnaruwa (Dias and Peiris 2015b: 26), Anuradhapura, Colombo, Galle, Gampaha, Kurunegala, Polonnaruwa, Puttalam, Ratnapura (Dias and Rajapaksa 2016: 34); *Secondary literature records*: Ceylon (Wheeler 1922: 974), Ceylon (Emery 1925: 125), Ceylon (Donisthorpe 1942b: 458), Ceylon (Menozzi 1934: 165), Ceylon (Chapman and Capco 1951: 242), Ceylon (Prins 1963: 107), Ceylon (Prins 1964: 92), Sri Lanka (Ali 1992: 8), Sri Lanka (Tiwari 1999: 69), Sri Lanka (Tiwari et al. 1999: 271), Sri Lanka (Mathew and Tiwari 2000: 356), Sri Lanka (Dias 2002: 18), Sri Lanka (Tiwari and Tiwari 2002: 160), Sri Lanka (Tak and Rathore 2004a: 169), Sri Lanka (Tiwari et al. 2004: 617), Sri Lanka (Dias 2006a: 50), Sri Lanka (Dias 2006b: 8), Sri Lanka (Rajan et al. 2006: 170), Sri Lanka (Tak 2008: 38), Sri Lanka (Tak and Kazmi 2011: 46), Sri Lanka (Dias et al. 2012: 19); *AntWeb records*: Kandy: SAM-HYM-C 005593 (AntWeb 2020).

***Camponotus sesquipedalis* Roger, 1863**

*Camponotus sesquipedalis* Roger, 1863: 137. TL: [Ceylon] Sri Lanka [Syntype: ZMHB?].

**Distribution.***Primary literature records*: Ceylon (Roger 1863: 137); *Secondary literature records*: Ceylon (Emery 1925: 90), Ceylon (Chapman and Capco 1951: 250), Sri Lanka (Dias 2002: 18), Sri Lanka (Dias 2006a: 50), Sri Lanka (Dias et al. 2012: 19).

***Camponotus simoni* Emery, 1893**

*Camponotus simoni* Emery, 1893a: 250. TL: Kottawa, Kandy: [Ceylon] Sri Lanka [Syntypes: MSNG]. [Images of CASENT 0905249, 0905250 syntype workers examined].

**Distribution.** Wet Zone; *Primary literature records*: Kottawa, Kandy (Emery 1893a: 250), Pattipola, Puwakpitiya, Peradeniya (Forel 1908: 6), Ceylon (Forel 1911a: 227); *Secondary literature records*: Ceylon (Emery 1896: 768), Ceylon (Emery 1925: 90), Ceylon (Chapman and Capco 1951: 250), Sri Lanka (Dias 2002: 18), Sri Lanka (Dias 2006a: 50), Sri Lanka (Dias et al. 2012: 18); *AntWeb records*: Kottawa, Kandy, Up Country: CASENT 0905249, 0905250, CASENT 0906989 (AntWeb 2020).

***Camponotus sklarus* Bolton, 1995**

*Camponotus sklarus* Bolton, 1995: 124. TL: Kandy: [Ceylon] Sri Lanka [Syntype: NHMUK]. [Images of CASENT 0903596 syntype worker examined].

**Distribution.** Wet Zone; *Primary literature records*: Ceylon (Chapman and Capco 1951: 250), Kandy (Bolton 1995: 124); *Secondary literature records*: Sri Lanka (Tiwari 1999: 71), Sri Lanka (Dias 2002: 18), Sri Lanka (Li-Zhong 2006: 264); *AntWeb records*: Kandy, Sri Lanka: CASENT 0903596 (AntWeb 2020).

***Camponotus thraso* Forel, 1893**

Camponotus maculatus r. thraso Forel, 1893b: 432. TL: [Ceylon] Sri Lanka [Syntype: MHNG]. [Images of CASENT 0910117 syntype worker examined].

**Distribution.** Dry Zone; *Primary literature records*: Ceylon (Forel 1893b: 433), Trincomalee, Anuradhapura (Emery 1901: 121); *Secondary literature records*: Ceylon (Emery 1925: 92), Ceylon (Chapman and Capco 1951: 251), Sri Lanka (Tiwari 1999: 72), Sri Lanka (Dias 2002: 18), Sri Lanka (Dias 2006a: 50), Sri Lanka (Dias et al. 2012: 18); *AntWeb records*: Sri Lanka: CASENT 0910117 (AntWeb 2020).

***Camponotus thraso diogenes* Forel, 1909**

Camponotus maculatus subsp. diogenes Forel, 1909: 396. TL: [Ceylon] Sri Lanka [Syntypes: MHNG]. [Images of CASENT 0910120, 0910121 syntype workers examined].

**Distribution.** Wet Zone; *Primary literature records*: Ceylon (Forel 1909: 396), Ambalagoda (Forel 1912c: 63); *Secondary literature records*: Ceylon (Chapman and Capco 1951: 251); *AntWeb records*: Sri Lanka: CASENT 0910120, 0910121 (AntWeb 2020).

***Camponotus varians* Roger, 1863**

*Camponotus varians* Roger, 1863: 138. TL: [Ceylon] Sri Lanka [Syntypes: ZMHB]. [Images of FOCOL 2276, 2277 syntype workers examined].

**Distribution.** Wet and Intermediate Zones; *Primary literature records*: Ceylon (Roger 1863: 139), Ceylon (Forel 1892a: 232), Kandy, Matale (Emery 1893a: 252); *Secondary literature records*: Ceylon (Emery 1896: 772), Ceylon (Emery 1925: 139), Ceylon (Chapman and Capco 1951: 234), Sri Lanka (Dias 2002: 18), Sri Lanka (Dias 2006a: 50), Sri Lanka (Dias et al. 2012: 19); *AntWeb records*: Kandy: CASENT 0906967, FOCOL 2276, 2277 (AntWeb 2020).

***Camponotus variegatus* (Smith F, 1858)**

*Formica variegata* Smith F, 1858: 19. TL: [Ceylon] Sri Lanka [Syntypes: NHMUK]. [Images of CASENT 0903586, 0903587 syntype worker and queen examined].

**Distribution.** Wet, Dry and Intermediate Zones; *Primary literature records*: Ceylon (Smith, F. 1858: 19), Trincomalee, Kandy (Emery 1893a: 252), Ceylon (Emery 1901: 121), Ceylon (Forel 1909: 397), Peradeniya, Seenigoda (Forel 1911a: 227), Peradeniya, Seenigoda, Haputale (Forel 1913a: 125); *Secondary literature records*: Ceylon (Emery 1925: 95), Ceylon (Donisthorpe 1942b: 458), Ceylon (Chapman and Capco 1951: 251), Sri Lanka (Tiwari 1999: 71), Sri Lanka (Dias 2002: 18), Sri Lanka (Dias 2006a: 50), Sri Lanka (Dias 2006b: 8), Sri Lanka (Li-Zhong 2006: 265), Sri Lanka (Tak 2008: 37), Sri Lanka (Tak 2010: 141), Sri Lanka (Dias et al. 2012: 19); *AntWeb records*: Sri Lanka: CASENT 0903587 (AntWeb 2020).

***Camponotus variegatus bacchus* (Smith F, 1858)**

*Formica bacchus* Smith F, 1858: 21. TL: [Ceylon] Sri Lanka [Syntype: NHMUK]. [Images of CASENT 0903588 syntype worker examined].

**Distribution.** Wet and Intermediate Zones; *Primary literature records*: Ceylon (Smith, F. 1858: 21), Ceylon (Forel 1892a: 242), Colombo (Forel 1907b: 19), Pattipola, Puwakpitiya, Galle (Forel 1908: 6), Ceylon (Forel 1909: 397), Peradeniya (Forel 1911a: 227), Ceylon (Forel 1913a: 125), Nalanda (Forel 1913b: 664); *Secondary literature records*: Ceylon (Emery 1925: 95), Ceylon (Chapman and Capco 1951: 251); *AntWeb records*: Sri Lanka: CASENT 0903588, 0903589 (AntWeb 2020).

***Camponotus variegatus crassinodis* Forel, 1892**

Camponotus mitis var. crassinodis Forel, 1892a: 230. TL: Myanmar [Syntypes: MHNG]. [Images of CASENT 0910165, 0910166 syntype workers examined].

**Distribution.** Wet Zone; *Primary literature records*: Ceylon (Forel 1909: 397), Peradeniya (Forel 1913a: 125); *Secondary literature records*: Ceylon (Chapman and Capco 1951: 249).

***Camponotus variegatus dulcis* Dalla Torre, 1893**

Camponotus mitis var. dulcis Dalla Torre, 1893: 243. TL: Bhamò [Birmania]: Myanmar [Syntype: MSNG]. [Images of CASENT 0905329 syntype worker examined].

**Distribution.***Primary literature records*: Ceylon (Forel 1892a: 243).

***Camponotus variegatus fuscithorax* Dalla Torre, 1893**

Camponotus mitis var. fuscithorax Dalla Torre, 1893: 243. TL: [Calcutta] Kolkata, West Bengal: India [Syntype: MHNG]. [Images of CASENT 0910167 syntype worker examined].

**Distribution.** Intermediate Zone; *Primary literature records*: Ceylon (Forel 1892a: 243), Kandy, Nuwara Eliya (Emery 1893a: 252), Ceylon (Emery 1901: 121), Ceylon (Forel 1909: 397), Dividsogala, Peradeniya (Forel 1911a: 228), Peradeniya, Seenigoda (Forel 1913a: 125); *Secondary literature records*: Ceylon (Emery 1925: 96), Ceylon (Chapman and Capco 1951: 251).

***Camponotus variegatus infuscus* Forel, 1892**

Camponotus maculatus r. infuscus Forel, 1892a: 242. TL: [Ceylon] Sri Lanka [Syntypes: MHNG]. [Images of CASENT 0910173, 0910174 syntype workers examined].

**Distribution.** Wet and Intermediate Zones; *Primary literature records*: Ceylon (Forel 1892a: 242), Nuwara Eliya (Emery 1893a: 252), Nuwara Elyia (Emery 1901: 121), Pidurutalagala, Hakgala, Pattipola (Forel 1908: 6), Haputale (Forel 1913a: 126); *Secondary literature records*: Ceylon (Emery 1925: 96), Ceylon (Chapman and Capco 1951: 252), Sri Lanka (Rajan et al. 2006: 169); *AntWeb records*: Sri Lanka: CASENT 0910173, 0910174 (AntWeb 2020).

***Camponotus variegatus intrans* Forel, 1911**

Camponotus maculatus subsp. intrans Forel, 1911c: 49 TL: Namunukula: [Ceylon] Sri Lanka [Syntypes: MHNG]. [Images of CASENT 0910171, 0910172 syntype workers examined].

**Distribution.** Intermediate Zone; *Primary literature records*: Namunukula (Forel: 1911c: 50); *Secondary literature records*: Ceylon (Emery 1925: 96), Ceylon (Chapman and Capco 1951: 252); *AntWeb records*: Namunukula: CASENT 0910171, 0910172 (AntWeb 2020).

***Camponotus variegatus somnificus* Forel, 1902**

Camponotus maculatus r. somnificus Forel, 1902a: 287. TL: Coonoor (Nilgiris), Tamil Nadu: India [Syntype: MHNG]. [Images of CASENT 0910185 syntype worker examined].

**Distribution.** Wet and Intermediate Zones; *Primary literature records*: Hakgala, Bandarawela (Forel 1908: 6), Haputale (Forel 1913a: 128); *Secondary literature records*: Sri Lanka (Tiwari 1999: 72), Ceylon (Chapman and Capco 1951: 252).

***Camponotus wedda* Forel, 1908**

*Camponotus wedda* Forel, 1908: 6. TL: [Ceylon] Sri Lanka [Syntypes: MHNG]. [Images of CASENT 0910543, 0910544 syntype workers examined].

**Distribution.** Wet Zone; *Primary literature records*: Ceylon (Forel 1908: 6), Ambalangoda (Forel 1909: 399), Ambalagoda (Forel 1912c: 67); *Secondary literature records*: Ceylon (Emery 1925: 140), Ceylon (Chapman and Capco 1951: 234), Sri Lanka (Dias 2002: 18), Sri Lanka (Dias 2006a: 50), Sri Lanka (Dias et al. 2012: 18); *AntWeb records*: Sri Lanka: CASENT 0910543, 0910544 (AntWeb 2020).

#### *Colobopsis*: 2 species

***Colobopsis badia* (Smith F, 1857)**

*Formica badia* Smith F, 1857: 54. TL: Singapore [Syntypes: NHMUK, OUMNH]. [Images of CASENT 0901897, CASENT 0903597 syntype workers examined].

**Distribution.***Primary literature records*: Ceylon (Emery 1925: 150); *Secondary literature records*: Sri Lanka (Dias 2002: 18), Sri Lanka (Li-Zhong 2006: 264); *AntWeb records*: Sri Lanka: CASENT 0903597 (AntWeb 2020).

***Colobopsis ceylonica* (Emery, 1925)**

*Camponotus ceylonicus* Emery, 1925: 145. TL: [Ceylon] Sri Lanka [Syntype: MSNG?].

**Distribution.***Primary literature records*: Ceylon (Emery 1925: 145); *Secondary literature records*: Ceylon (Emery 1925: 145), Ceylon (Chapman and Capco 1951: 223), Sri Lanka (Dias 2002: 18).

#### *Lepisiota*: 7 species/subspecies

***Lepisiota capensis* (Mayr, 1862)**

*Acantholepis capensis* Mayr, 1862: 699. TL: [Cap] Western Cape: South Africa [Syntypes: NHMW, ZMHB]. [Images of CASENT 0915714, 0915715, FOCOL 2208 syntype workers examined].

**Distribution.** Dry and Intermediate Zones; *Primary literature records*: Ceylon (Forel 1894: 414), Anuradhapura, Polonnaruwa (Dias and Kosgamage 2012: 61), Sri Lanka (Dias 2014: 116), Namalweva, Ihakuluweva, Marawila, Pallama, Egodayagama (Dias and Peiris 2015a: 5), Anuradhapura, Kurunegala, Polonnaruwa, Puttalam (Dias and Rajapaksa 2016: 34); *Secondary literature records*: Sri Lanka (Dias 2002: 18), Sri Lanka (Dias 2006a: 50), Sri Lanka (Dias 2006b: 8), Sri Lanka (Li-Zhong 2006: 262), Sri Lanka (Rajan et al. 2006: 171), Sri Lanka (Dias et al. 2012: 19).

***Lepisiota fergusoni* (Forel, 1895)**

*Acantholepis fergusoni* Forel, 1895a: 459. TL: Travancore: India [Syntypes: MHNG]. [Images of CASENT 0909883 syntype worker examined].

**Distribution.** Wet and Dry Zones; *Primary literature records*: Jayanthipura (Dias and Kosgamage 2012: 61), Namalweva, Marawila, Madurankuliya (Dias and Peiris 2015a: 5), Anuradhapura, Colombo, Galle, Gampaha, Polonnaruwa, Puttalam, Ratnapura (Dias and Rajapaksa 2016: 34).

***Lepisiota frauenfeldi* (Mayr, 1855)**

*Hypoclinea frauenfeldi* Mayr, 1855: 378. TL: [Syracus] Sicily: Italy; Attica: Greece; El Kantara: Algeria [Syntypes: MHNG, ZMHB]. [Images of CASENT 0909884, FOCOL 2199–2201 syntype workers and queen examined].

**Distribution.** Wet and Dry Zones; *Primary literature records*: Sri Lanka (Dias et al. 2012: 19), Sri Lanka (Dias 2014: 116), Anuradhapura, Colombo, Galle, Gampaha, Polonnaruwa, Puttalam, Ratnapura (Dias and Rajapaksa 2016: 34).

***Lepisiota lunaris* (Emery, 1893)**

*Acantholepis lunaris* Emery, 1893a: 250. TL: Colombo: [Ceylon] Sri Lanka [Syntypes: MSNG]. [Images of CASENT 0905157 syntype worker examined].

**Distribution.** Wet Zone; *Primary literature records*: Colombo (Emery 1893a: 250), Ceylon (Forel 1909: 395); *Secondary literature records*: Ceylon (Emery 1925: 24), Ceylon (Chapman and Capco 1951: 209); *AntWeb records*: Colombo: CASENT 0905157 (AntWeb 2020).

***Lepisiota opaca* (Forel, 1892)**

*Acantholepis opaca* Forel, 1892b: 43. TL: [Kanara], Karnataka: India [Syntypes: MHNG, MSNG]. [Images of CASENT 0905158, CASENT 0909893 syntype workers examined].

**Distribution.** Dry Zone; *Primary literature records*: Sri Lanka (Dias et al. 2012: 19), Sri Lanka (Dias 2014: 117), Anuradhapura, Polonnaruwa (Dias and Rajapaksa 2016: 34).

***Lepisiota pulchella* (Forel, 1892)**

Acantholepis opaca r. pulchella Forel, 1892b: 43. TL: [Poona] Pune [Syntypes: MHNG]. [Images of CASENT 0909894 syntype worker examined].

**Distribution.** Dry Zone; *Primary literature records*: Anuradhapura, Polonnaruwa (Dias and Rajapaksa 2016: 34).

***Lepisiota rothneyi wroughtonii* (Forel, 1902)**

Plagiolepis rothneyi r. wroughtonii Forel, 1902a: 292. TL: Ootacamune, Nilgiris, Tamil Nadu: India [Syntypes: MHNG]. [Images of CASENT 0909869 syntype worker examined].

**Distribution.** Wet Zone; *Primary literature records*: Bandarawela (Forel 1913b: 663).

#### *Myrmoteras*: 1 species

***Myrmoteras ceylonicum* Gregg, 1957**

*Myrmoteras ceylonica* Gregg, 1957: 41. TL: Udawattakele Sanctuary, Kandy: [Ceylon] Sri Lanka [Paratype: FMNH]. [Images of FMNHINS 0000062663 paratype worker examined].

**Distribution.** Wet Zone; *Primary literature records*: Udawattakele Sanctuary, Kandy (Gregg 1957: 44), Udawattakele Sanctuary, Kandy (Moffett 1985: 27); *Secondary literature records*: Sri Lanka (Dias 2006a: 50), Sri Lanka (Zettel and Sorger 2011: 66), Sri Lanka (Dias et al. 2012: 19); *AntWeb records*: Kandy, Udawattakele Sanctuary: FMNHINS 0000062663 (AntWeb 2020).

#### *Nylanderia*: 7 species/subspecies

***Nylanderia bourbonica* (Forel, 1886)**

Prenolepis nodifera r. bourbonica Forel, 1886: 210. TL: St. Denis, Réunion [Syntypes: MHNG].

**Distribution.** Wet Zone; *Primary literature records*: Sinharaja Forest Reserve (Gunawardene et al. 2008: 80).

***Nylanderia indica* (Forel, 1894)**

*Prenolepis indica* Forel, 1894: 409. TL: [Poona] Pune: India [Syntypes: MHNG]. [Images of CASENT 0911008 syntype worker examined].

**Distribution.** Wet and Dry Zones; *Primary literature records*: Ceylon (Forel 1894: 409), Ceylon (Emery 1901: 121), Hakgalla (Forel 1908: 4), Peradeniya, Seenigoda (Forel 1911a: 227), Anuradhapura, Polonnaruwa (Dias and Kosgamage 2012: 61); *Secondary literature records*: Ceylon (Emery 1925: 220), Sri Lanka (Ali 1992: 9), Sri Lanka (Tiwari et al. 1999: 276), Sri Lanka (Dias 2002: 19), Sri Lanka (Dias et al. 2012: 19).

***Nylanderia taylori* (Forel, 1894)**

*Prenolepis taylori* Forel, 1894: 410. TL: [Orissa] Odisha: India [Syntypes: MHNG]. [Images of CASENT 0911011 syntype worker examined].

**Distribution.** Dry Zone; *Primary literature records*: Ceylon (Forel 1902a: 292), Trincomalee (Forel 1913b: 663); *Secondary literature records*: Ceylon (Forel 1903a: 712), Ceylon (Emery 1925: 220), Sri Lanka (Tiwari et al. 1999: 276), Sri Lanka (Dias 2002: 19), Sri Lanka (Dias 2006a: 50), Sri Lanka (Li-Zhong 2006: 270), Sri Lanka (Tak 2008: 41), Sri Lanka (Dias et al. 2012: 19).

***Nylanderia taylori levis* (Forel, 1913)**

Prenolepis taylori r. levis Forel, 1913a: 104. TL: Peradeniya: [Ceylon] Sri Lanka [Syntype: MHNG]. [Images of CASENT 0911012 syntype worker examined].

**Distribution.** Wet Zone; *Primary literature records*: Peradeniya (Forel 1913a: 104); *Secondary literature records*: Ceylon (Emery 1925: 220), Ceylon (Chapman and Capco 1951: 217); *AntWeb records*: Peradeniya: CASENT 0911012 (AntWeb 2020).

***Nylanderia vagabunda* (Motschoulsky, 1863)**

*Paratrechina vagabunda* Motschoulsky, 1863: 13. TL: [Ceylon] Sri Lanka [Type: UNK].

**Distribution.***Primary literature records*: Ceylon (Motschoulsky 1863: 13); *Secondary literature records*: Ceylon (Emery 1925: 220), Ceylon (Chapman and Capco 1951: 218).

***Nylanderia vividula* (Nylander, 1846)**

*Formica vividula* Nylander, 1846a: 900. TL: Finland [Lectotype: MZH]. [Images of CASENT 0102536 lectotype worker examined].

**Distribution.***Primary literature records*: Ceylon (Chapman and Capco 1951: 218); *Secondary literature records*: Sri Lanka (Dias 2002: 19).

***Nylanderia yerburyi* (Forel, 1894)**

*Prenolepis yerburyi* Forel, 1894: 409. TL: [Ceylon] Sri Lanka [Syntype: MHNG]. [Images of CASENT 0911013 syntype worker examined].

**Distribution.** Wet, Dry and Intermediate Zones; *Primary literature records*: Nuwara Eliya (Emery 1893a: 250), Ceylon (Forel 1894: 409), Ceylon (Emery 1901: 121), Haputale, Pattipola (Forel 1913a: 104), Anuradhapura, Polonnaruwa (Dias and Kosgamage 2012: 61), Kirikanda Forest (Dias et al. 2013: 64), Sri Lanka (Dias 2014: 119), Kalugala Proposed Forest Reserve, Wilpita “Aranya Kele” (Dias and Ruchirani 2014: 88), Namalweva, Ihakuluweva, Marawila, Pallama, Madurankuliya, Egodayagama (Dias and Peiris 2015a: 5), Sinhapura, Polonnaruwa, Mawathagama, Kurunegala (Dias and Peiris 2015b: 26), Colombo, Gampaha, Galle, Ratnapura (Dias and Rajapaksa 2016: 35), Indikada Mukalana Forest Reserve (Udayakantha and Dias 2018: 72); *Secondary literature records*: Ceylon (Emery 1925: 220), Ceylon (Chapman and Capco 1951: 218), Sri Lanka (Tiwari 1999: 81), Sri Lanka (Dias 2002: 19), Sri Lanka (Dias 2006a: 50), Sri Lanka (Li-Zhong 2006: 270), Sri Lanka (Dias et al. 2012: 19), Sri Lanka (Wachkoo and Bharti 2015: 119); *AntWeb records*: Sri Lanka: CASENT 0911013 (AntWeb 2020).

#### *Oecophylla*: 1 species

***Oecophylla smaragdina* (Fabricius, 1775)**

*Formica smaragdina* Fabricius, 1775: 828. TL: India [Syntypes: ZMUK].

**Distribution.** Wet, Dry and Intermediate Zones; *Primary literature records*: Kandy, Colombo, Nawalapitiya (Emery 1893a: 250), Ceylon (Emery 1901: 121), Colombo (Forel 1904a: 387), Puwakpitiya, Peradeniya (Forel 1908: 6), Seenigoda, Peradeniya (Forel 1913a: 122), South Sri Lanka (Way and Bolton 1997: 443), Sri Lanka Cashew Corporation-West, western Sri Lanka, Panadura, Pushparanghnam Estate (Rickson and Rickson 1998: 843), Kelaniya, Gampaha, Colombo, Ratnapura, Galle (Dias 2006a: 45), Dambulla (Dias and Kosgamage 2008: 115), Sinharaja Forest Reserve (Gunawardene et al. 2008: 80), Nawalapitiya (Amarasinghe 2010: 12), Anuradhapura, Polonnaruwa (Dias and Kosgamage 2012: 61), Sri Lanka (Dias 2014: 120), Namalweva, Ihakuluweva, Marawila, Pallama, Madurankuliya, Egodayagama (Dias and Peiris 2015a: 5), Anuradhapura, Colombo, Galle, Gampaha, Kurunegala, Polonnaruwa, Puttalam, Ratnapura (Dias and Rajapaksa 2016: 35), Indikada Mukalana Forest Reserve (Dias and Udayakantha 2016b: 5), Indikada Mukalana Forest Reserve (Udayakantha and Dias 2018: 72); *Secondary literature records*: Ceylon (Viehmeyer 1912: 22), Emery 1925: 52 (Ceylon), Ceylon (Mukherji and Ribeiro 1925: 208), Ceylon (Menozzi 1932: 11), Ceylon (Donisthorpe 1942b: 457), Ceylon (Cole and Jones 1948: 642), Sri Lanka (Ali 1992: 8), Sri Lanka (Tiwari 1999: 66), Sri Lanka (Tiwari et al. 1999: 277), Sri Lanka (Mathew and Tiwari 2000: 345), Sri Lanka (Dias 2002: 19), Sri Lanka (Tiwari and Tiwari 2002: 159), Sri Lanka (Mathew 2003: 203), Sri Lanka (Tiwari et al. 2003: 496), Sri Lanka (Tak and Rathore 2004a: 172), Sri Lanka (Tiwari et al. 2004: 615), Sri Lanka (Dias 2006b: 8), Sri Lanka (Ghosh et al. 2006: 380), Sri Lanka (Li-Zhong 2006: 269), Sri Lanka (Rajan et al. 2006: 171), Sri Lanka (Sheela 2008a: 13), Sri Lanka (Tak 2008: 40), Sri Lanka (Tak and Kazmi 2011: 47), Sri Lanka (Dias et al. 2012: 19); *AntWeb records*: Kelaniya Jungle, Gal. Dist.: ANIC 32-043919, 32-043920 (AntWeb 2020).

#### *Paratrechina*: 1 species

***Paratrechina longicornis* (Latreille, 1802)**

*Formica longicornis* Latreille, 1802: 113. TL: Bangkok: Thailand [Neotype: ANIC].

**Distribution.** Wet, Dry and Intermediate Zones; *Primary literature records*: Galle, Colombo (Emery 1893a: 253), Ceylon (Forel 1894: 408), Ceylon (Emery 1901: 121), Galle (Forel 1908: 4), Seenigoda, Ambalangoda (Forel 1913a: 104), South Sri Lanka (Way and Bolton 1997: 443), eastern Sri Lanka, Sri Lanka Cashew Corporation-West, western Sri Lanka, Panadura, Pushparanghnam Estate (Rickson and Rickson 1998: 843), Kelaniya, Gampaha, Colombo, Ratnapura (Dias 2006a: 45), Sri Lanka (Dias 2006b: 8), Dambulla (Dias and Kosgamage 2008: 115), Nawalapitiya (Amarasinghe 2010: 12), Gilimale Forest Reserve (Dias and Perera 2011: 72), Sri Lanka (Dias et al. 2012: 19), Anuradhapura, Polonnaruwa (Dias and Kosgamage 2012: 61), Kirikanda Forest (Dias et al. 2013: 64), Sri Lanka (Dias 2014: 121), Namalweva, Ihakuluweva, Marawila, Pallama, Madurankuliya (Dias and Peiris 2015a: 5), Sinhapura, Polonnaruwa (Dias and Peiris 2015b: 26), Anuradhapura, Colombo, Galle, Gampaha, Kurunegala, Polonnaruwa, Puttalam, Ratnapura (Dias and Rajapaksa 2016: 35), Meethirigala Forest Reserve (Dias and Udayakantha 2016a: 53), Indikada Mukalana Forest Reserve (Dias and Udayakantha 2016b: 5), Indikada Mukalana Forest Reserve (Udayakantha and Dias 2018: 72); *Secondary literature records*: Sri Lanka (Dias 2002: 19), Sri Lanka (Tiwari and Tiwari 2002: 159), Sri Lanka (Tiwari et al. 2004: 619); *AntWeb records*: Hambantota, Puttalam, Colombo, Peradenyia: ANIC 32-053758, ANIC 32-053793, ANIC 32-053801, 32-053802 (AntWeb 2020).

#### *Plagiolepis*: 3 species

***Plagiolepis exigua* Forel, 1894**

*Plagiolepis exigua* Forel, 1894: 417. TL: [Java, Tandjong Slarmat]: Indonesia; [Poona] Pune, Mahrashtra: India [Syntypes: MHNG, SIZK]. [Images of CASENT 0101302, 0101303, 0101304, 0101305, CASENT 0101307, CASENT 0917872 syntype workers, queens and males examined].

**Distribution.** Dry Zone; *Primary literature records*: Nachchaduwa Forest (Dias and Kosgamage 2012: 61), Namalweva (Dias and Peiris 2015a: 5), Anuradhapura, Polonnaruwa (Dias and Rajapaksa 2016: 35).

***Plagiolepis jerdonii* Forel, 1894**

*Plagiolepis jerdonii* Forel, 1894: 416. TL: [Poona] Pune, Mahrashtra: India [Syntype: MHNG]. [Images of CASENT 0909852 syntype worker examined].

**Distribution.** Dry Zone; *Primary literature records*: Sri Lanka (Dias et al. 2012: 19), Giritale Forest (Dias and Kosgamage 2012: 62), Marawila, Pallama, Madurankuliya (Dias and Peiris 2015a: 5).

***Plagiolepis pissina* Roger, 1863**

*Plagiolepis pissina* Roger, 1863: 162. TL: [Ceylon] Sri Lanka [Syntypes: ZMHB]. [Images of FOCOL 2225, 2226 syntype worker and queen examined].

**Distribution.***Primary literature records*: Ceylon (Roger 1863: 162), Ceylon (Forel 1894: 417); *Secondary literature records*: Ceylon (Emery 1925: 21), Ceylon (Chapman and Capco 1951: 214), Sri Lanka (Dias 2002: 18), Sri Lanka (Dias 2006a: 50), Sri Lanka (Dias et al. 2012: 19); *AntWeb records*: Sri Lanka: FOCOL 2225, 2226 (AntWeb 2020).

#### *Polyrhachis*: 34 species/subspecies

***Polyrhachis aculeata* Mayr, 1879**

*Polyrhachis aculeata* Mayr, 1879: 657. TL: Indonesia [Holotype: NHMW]. [Images of CASENT 0915814 syntype worker examined].

**Distribution.***Primary literature records*: Ceylon (Forel 1893a: 28); *Secondary literature records*: Ceylon (Emery 1925: 205), Ceylon (Chapman and Capco 1951: 255), Sri Lanka (Tiwari 1999: 80), Sri Lanka (Dias 2002: 19), Sri Lanka (Kohout 2013: 142).

***Polyrhachis bugnioni* Forel, 1908**

*Polyrhachis bugnioni* Forel, 1908: 11. TL: Puwakpitiya, Up country: [Ceylon] Sri Lanka [Syntype: MHNG]. [Images of CASENT 0910965 syntype worker examined].

**Distribution.** Wet Zone; *Primary literature records*: Puwakpitiya (Forel 1908: 12), Ceylon (Forel 1909: 402), Puwakpitiya, Gilimale, Induruwa (Dorow and Kohout 1995: 98), Sinharaja Forest Reserve (Gunawardene et al. 2008: 80), Sinharaja Forest Reserve (Gunawardene et al. 2012: 84), Sri Lanka (Dias 2014: 125), Colombo, Galle, Gampaha, Ratnapura (Dias and Rajapaksa 2016: 35), Indikada Mukalana Forest Reserve (Dias and Udayakantha 2016b: 5), Indikada Mukalana Forest Reserve (Udayakantha and Dias 2018: 72); *Secondary literature records*: Ceylon (Emery 1925: 210), Ceylon (Chapman and Capco 1951: 256), Sri Lanka (Dias 2002: 19), Sri Lanka (Dias 2006a: 50), Sri Lanka (Dias et al. 2012: 19); *AntWeb records*: Up country: CASENT 0910965 (AntWeb 2020).

***Polyrhachis convexa* Roger, 1863**

*Polyrhachis convexa* Roger, 1863: 153. TL: [Ceylon] Sri Lanka [Syntype: ZMHB]. [Images of FOCOL 2615 syntype worker examined].

**Distribution.** Wet and Dry Zones; *Primary literature records*: Ceylon (Roger 1863: 154), Ceylon (Forel 1893a: 29), Ceylon (Forel 1909: 401), Sinharaja Forest Reserve (Gunawardene et al. 2008: 80), Anuradhapura, Polonnaruwa (Dias and Kosgamage 2012: 62), Kirikanda Forest (Dias et al. 2013: 64), Sri Lanka (Dias 2014: 127), Anuradhapura, Colombo, Galle, Gampaha, Polonnaruwa, Ratnapura (Dias and Rajapaksa 2016: 35); *Secondary literature records*: Ceylon (Emery 1896: 777), Ceylon (Emery 1925: 204), Ceylon (Chapman and Capco 1951: 270), Sri Lanka (Dias 2002: 19), Sri Lanka (Mathew and Tiwari 2000: 361), Sri Lanka (Ghosh et al. 2006: 381), Sri Lanka (Li-Zhong 2006: 271), Sri Lanka (Dias et al. 2012: 19); *AntWeb records*: Kandy, Sri Lanka: CASENT 0906801, FOCOL 2615 (AntWeb 2020).

***Polyrhachis convexa isabellae* Forel, 1908**

*Polyrhachis convexa isabellae* Forel, 1908: 9. TL: Hakgala, Puwakpitiya: [Ceylon] Sri Lanka [Syntype: MHNG]. [Images of CASENT 0910934 syntype worker examined].

**Distribution.** Wet Zone; *Primary literature records*: Hakgala, Puwakpitiya (Forel 1908: 10); *Secondary literature records*: Ceylon (Emery 1925: 204), Ceylon (Chapman and Capco 1951: 270); *AntWeb records*: Hakgala: CASENT 0910934 (AntWeb 2020).

***Polyrhachis curvispina* Forel, 1908**

Polyrhachis oedipus var. curvispina Forel, 1908: 8. TL: Puwakpitiya: [Ceylon] Sri Lanka [Syntypes: MHNG, NHMUK]. [Images of CASENT 0903370, CASENT 0910875 syntype workers examined].

**Distribution.** Wet Zone; *Primary literature records*: Puwakpitiya (Forel 1908: 9); *Secondary literature records*: Ceylon (Emery 1925: 193), Ceylon (Chapman and Capco 1951: 294); *AntWeb records*: Puwakpitiya: CASENT 0903370, CASENT 0910875 (AntWeb 2020).

***Polyrhachis dives* Smith F, 1857**

*Polyrhachis dives* Smith F, 1857: 64. TL: Singapore [Syntype: NHMUK]. [Images of CASENT 0903388 holotype worker examined].

**Distribution.***Primary literature records*: Ceylon (Forel 1893a: 34); *Secondary literature records*: Sri Lanka (Ali 1992: 8), Sri Lanka (Tiwari 1999: 75), Sri Lanka (Tiwari and Tiwari 2002: 162), Sri Lanka (Tiwari et al. 2003: 498), Sri Lanka (Ghosh et al. 2006: 381), Sri Lanka (Li-Zhong 2006: 271).

***Polyrhachis dives belli* Forel, 1912**

Polyrhachis dives subsp. belli Forel, 1912c: 74. TL: [Ceylon] Sri Lanka [Syntype: MHNG]. [Images of CASENT 0910885 syntype worker examined].

**Distribution.***Primary literature records*: Ceylon (Forel 1912c: 75); *Secondary literature records*: Ceylon (Emery 1925: 195), Ceylon (Chapman and Capco 1951: 289); *AntWeb records*: Sri Lanka: CASENT 0910885 (AntWeb 2020).

***Polyrhachis exercita* (Walker, 1859)**

*Formica exercita* Walker, 1859: 370. TL: [Ceylon] Sri Lanka [Holotype: NHMUK]. [Images of CASENT 0903298 holotype queen examined].

**Distribution.** Wet Zone; *Primary literature records*: Ceylon (Walker 1859: 370), Colombo, Kandy (Emery 1893a: 254), Ceylon (Forel 1893a: 29), Ceylon (Emery 1901: 122), Ceylon (Forel 1909: 401), Puwakpitiya (Forel 1908: 9), Peradeniya, Lady Black Drive (Forel 1911a: 228), Peradeniya, Seenigoda (Forel 1913a: 134); *Secondary literature records*: Ceylon (Emery 1896: 776), Ceylon (Emery 1925: 178), Ceylon (Donisthorpe 1942b: 459), Ceylon (Chapman and Capco 1951: 199), Sri Lanka (Ali 1992: 9), Sri Lanka (Tiwari 1999: 75), Sri Lanka (Tiwari et al. 1999: 272), Sri Lanka (Dias 2002: 19), Sri Lanka (Tak and Rathore 2004a: 171), Sri Lanka (Rajan et al. 2006: 172), Sri Lanka (Dias 2006a: 50), Sri Lanka (Dias et al. 2012: 19).

***Polyrhachis fornicata* Emery, 1900**

Polyrhachis rastellata subsp. fornicata Emery, 1900: 720. TL: [Giava] Java: Indonesia [Syntype: MSNG]. [Images of CASENT 0905540 syntype worker examined].

**Distribution.** Wet Zone; *Primary literature records*: Ceylon (Emery 1901: 122), Puwakpitiya (Forel 1908: 9), Ceylon (Forel 1911a: 228); *Secondary literature records*: Ceylon (Emery 1925: 208), Ceylon (Chapman and Capco 1951: 266).

***Polyrhachis frauenfeldi* Mayr, 1862**

*Polyrhachis frauenfeldi* Mayr, 1862: 687. TL: Java: Indonesia [Syntype: NHMW]. [Images of CASENT 0915820 syntype worker examined].

**Distribution.***Primary literature records*: Ceylon (Forel 1893a: 28).

***Polyrhachis gibbosa* Forel, 1908**

Polyrhachis aculeata var. gibbosa Forel, 1908: 9. TL: Puwakpitiya: [Ceylon] Sri Lanka [Syntype: MHNG]. [Images of CASENT 0910939 syntype worker examined].

**Distribution.** Wet Zone; *Primary literature records*: Puwakpitiya (Forel 1908: 9), Ceylon (Forel 1909: 402), Seenigoda (Forel 1911a: 228), Sinharaja Forest Reserve (Gunawardene et al. 2008: 80), Puwakpitiya, Sinharaja Forest Reserve (Kohout 2013: 148); *Secondary literature records*: Ceylon (Emery 1925: 205), Ceylon (Chapman and Capco 1951: 255); *AntWeb records*: Sri Lanka: CASENT 0910939 (AntWeb 2020).

***Polyrhachis hippomanes ceylonensis* Emery, 1893**

Polyrhachis hippomanes subsp. ceylonensis Emery, 1893a: 254. TL: Kandy: [Ceylon] Sri Lanka [Syntype: MSNG]. [Images of CASENT 0905632 syntype worker examined].

**Distribution.** Wet Zone; *Primary literature records*: Kottawa, Kandy (Emery 1893a: 254), Puwakpitiya (Forel 1908: 9), Ceylon (Forel 1909: 402), Sinharaja Forest Reserve (Gunawardene et al. 2008: 80); *Secondary literature records*: Ceylon (Emery 1896: 779), Ceylon (Emery 1925: 195), Ceylon (Chapman and Capco 1951: 291), Sri Lanka (Mathew 1984: 308), Sri Lanka (Mathew and Tiwari 2000: 360), Sri Lanka (Ghosh et al. 2006: 381); *AntWeb records*: Kandy: CASENT 0905632 (AntWeb 2020).

***Polyrhachis horni* Emery, 1901**

*Polyrhachis horni* Emery, 1901: 122. TL: Nalanda: [Ceylon] Sri Lanka [Syntypes: DEIC, MSNG]. [Images of CASENT 0905539, FOCOL 0086–0089 syntype workers and queen examined].

**Distribution.** Wet and Intermediate Zones; *Primary literature records*: Nalanda (Emery 1901: 122), Nalanda (Forel 1903a: 712), Puwakpitiya (Forel 1908: 8); *Secondary literature records*: Ceylon (Emery 1925: 201), Ceylon (Chapman and Capco 1951: 271), Sri Lanka (Ali 1992: 9), Sri Lanka (Dias 2002: 19), Sri Lanka (Dias 2006a: 51), Sri Lanka (Dias et al. 2012: 19); *AntWeb records*: Deniyaya, Nalanda: CASENT 0905539, CASENT 0906807, FOCOL 0086–0089 (AntWeb 2020).

***Polyrhachis illaudata* Walker, 1859**

*Polyrhachis illaudatus* Walker, 1859: 373. TL: [Ceylon] Sri Lanka [Holotype: NHMUK]. [Images of CASENT 0903443 holotype worker examined].

**Distribution.** Wet Zone; *Primary literature records*: Ceylon (Walker 1859: 373), Kandy (Emery 1893a: 255), Ceylon (Forel 1893a: 29), Ceylon (Emery 1901: 122), Peradeniya (Forel 1908: 9), Dividsogala (Forel 1911a: 228), Ambalagoda (Forel 1912c: 70), Sri Lanka (Bolton 1974b: 176), Sinharaja Forest Reserve (Gunawardene et al. 2008: 80), Sri Lanka (Dias 2014: 127), Kalugala Proposed Forest Reserve (Dias and Ruchirani 2014: 88), Colombo, Galle, Gampaha, Ratnapura (Dias and Rajapaksa 2016: 35); *Secondary literature records*: Ceylon (Emery 1896: 777, 780), Ceylon (Emery 1925: 209), Ceylon (Donisthorpe 1942b: 460), Ceylon (Chapman and Capco 1951: 271), Sri Lanka (Tiwari 1999: 74), Sri Lanka (Tiwari et al. 1999: 273), Sri Lanka (Mathew 2000: 353), Sri Lanka (Mathew and Tiwari 2000: 362), Sri Lanka (Dias 2002: 19), Sri Lanka (Tiwari and Tiwari 2002: 161), Sri Lanka (Mathew 2003: 204), Sri Lanka (Tiwari et al. 2003: 498), Sri Lanka (Ghosh et al. 2006: 382), Sri Lanka (Jaitrong and Nabhitabhata 2005: 39), Sri Lanka (Dias 2006a: 51), Sri Lanka (Sheela 2008a: 16), Sri Lanka (Dias et al. 2012: 19), Sri Lanka (Andersen et al. 2013: 144); *AntWeb records*: Sri Lanka: CASENT 0903443.

***Polyrhachis jerdonii* Forel, 1892**

*Polyrhachis jerdonii* Forel, 1892c: 17. TL: [Ceylon] Sri Lanka [Syntype: MHNG]. [Images of CASENT 0910853 syntype worker examined].

**Distribution.** Wet and Dry Zones; *Primary literature records*: Ceylon (Forel 1892c: 17), Ceylon (Forel 1893a: 28), Anuradhapura (Emery 1901: 122), Sri Lanka (Kohout 2006: 146), Anuradhapura, Polonnaruwa (Dias and Kosgamage 2012: 62), Kirikanda Forest (Dias et al. 2013: 64), Sri Lanka (Dias 2014: 127), Anuradhapura, Polonnaruwa, Puttalam (Dias and Rajapaksa 2016: 35); *Secondary literature records*: Ceylon (Emery 1896: 779), Ceylon (Emery 1925: 191), Ceylon (Chapman and Capco 1951: 292), Sri Lanka (Dias 2002: 19), Sri Lanka (Dias 2006a: 51), Sri Lanka (Dias et al. 2012: 19); *AntWeb records*: Sri Lanka: CASENT 0910853 (AntWeb 2020).

***Polyrhachis lacteipennis* Smith F, 1858**

*Polyrhachis lacteipennis* Smith F, 1858: 60. TL: North India [Holotype: NHMUK]. [Images of CASENT 0903386 holotype queen examined].

**Distribution.***Primary literature records*: Ceylon (Forel 1893a: 34), Ceylon (Emery 1901: 122); *Secondary literature records*: Ceylon (Emery 1925: 196), Sri Lanka (Tiwari 1999: 75), Sri Lanka (Tiwari et al. 1999: 273), Sri Lanka (Dias 2002: 19), Sri Lanka (Tiwari et al. 2004: 618), Sri Lanka (Tak et al. 2007: 120).

***Polyrhachis nigra* Mayr, 1862**

*Polyrhachis niger* Mayr, 1862: 683. TL: [Ceylon] Sri Lanka [Syntype: NHMW]. [Images of CASENT 0915821 syntype queen examined].

**Distribution.***Primary literature records*: Ceylon (Mayr 1862: 683); *Secondary literature records*: Ceylon (Emery 1896: 777), Ceylon (Emery 1925: 201), Ceylon (Chapman and Capco 1951: 273), Sri Lanka (Dias 2002: 19), Sri Lanka (Dias 2006a: 51), Sri Lanka (Dias et al. 2012: 19); *AntWeb records*: Sri Lanka: CASENT 0915821 (AntWeb 2020).

***Polyrhachis oedipus* Forel, 1893**

*Polyrhachis oedipus* Forel, 1893a: 31. TL: [Ceylon] Sri Lanka [Syntype: MHNG]. [Images of CASENT 0910874 syntype worker examined].

**Distribution.** Wet Zone; *Primary literature records*: Kandy (Emery 1893a: 255), Ceylon (Forel 1893a: 31), Puwakpitiya (Forel 1908: 8), Ceylon (Forel 1909: 401), Sinharaja Forest Reserve (Gunawardene et al. 2008: 80); *Secondary literature records*: Ceylon (Emery 1896: 779), Ceylon (Emery 1925: 193), Ceylon (Chapman and Capco 1951: 294), Sri Lanka (Dias 2002: 19); *AntWeb records*: Sri Lanka: CASENT 0910874 (AntWeb 2020).

***Polyrhachis proxima* Roger, 1863**

*Polyrhachis proxima* Roger, 1863: 155. TL: Linga, Victoria: Australia [Syntypes: ZMHB]. [Images of FOCOL 2624, 2625 syntype workers examined].

**Distribution.***Primary literature records*: Ceylon (Emery 1925: 202); *Secondary literature records*: Ceylon (Chapman and Capco 1951: 274), Sri Lanka (Dias 2002: 19), Sri Lanka (Mathew and Tiwari 2000: 365).

***Polyrhachis punctillata* Roger, 1863**

*Polyrhachis punctillata* Roger, 1863: 152. TL: [Ceylon] Sri Lanka [Syntypes: ZMHB]. [Images of FOCOL 2620–2622 syntype workers and queen examined].

**Distribution.** Dry and Intermediate Zones; *Primary literature records*: Ceylon (Roger 1863: 153), Ceylon (Forel 1893a: 29), Anuradhapura, Polonnaruwa (Dias and Kosgamage 2012: 62), Sri Lanka (Dias 2014: 128), Namalweva, Egodayagama (Dias and Peiris 2015a: 5), Sinhapura, Polonnaruwa (Dias and Peiris 2015b: 26), Anuradhapura, Kurunegala, Polonnaruwa, Puttalam (Dias and Rajapaksa 2016: 35); *Secondary literature records*: Ceylon (Emery 1896: 777), Ceylon (Forel 1902a: 289), Ceylon (Emery 1925: 204), Ceylon (Chapman and Capco 1951: 275), Sri Lanka (Tiwari 1999: 77), Sri Lanka (Mathew and Tiwari 2000: 365), Sri Lanka (Dias 2002: 19), Sri Lanka (Tak and Rathore 2004a: 170), Sri Lanka (Tiwari et al. 2004: 618), Sri Lanka (Dias 2006a: 51), Sri Lanka (Li-Zhong 2006: 271), Sri Lanka (Rajan et al. 2006: 173), Sri Lanka (Dias et al. 2012: 19); *AntWeb records*: Sri Lanka: FOCOL 2620–2622 (AntWeb 2020).

***Polyrhachis rastellata* (Latreille, 1802)**

*Formica rastellata* Latreille, 1802: 130. TL: Indes Orientales: Indonesia [Holotype: MNHN (Lost)].

**Distribution.** Wet Zone; *Primary literature records*: Kandy, Wackwella, Nawalapitiya (Emery 1893a: 254), Ceylon (Forel 1893a: 30), Ceylon (Forel 1909: 402), Colombo (Dias 2006a: 45), Sinharaja Forest Reserve (Gunawardene et al. 2008: 80), Sri Lanka (Dias 2014: 123), Gonapola (Dias 2015: 125), Colombo, Galle, Gampaha, Ratnapura (Dias and Rajapaksa 2016: 35); *Secondary literature records*: Ceylon (Emery 1925: 208), Ceylon (Chapman and Capco 1951: 265), Sri Lanka (Tiwari 1999: 76), Sri Lanka (Dias 2002: 19), Sri Lanka (Dias 2006b: 8), Sri Lanka (Li-Zhong 2006: 271), Sri Lanka (Dias et al. 2012: 19); *AntWeb records*: Sri Lanka: CASENT 0903407, CASENT 0912103 (AntWeb 2020).

***Polyrhachis rupicapra* Roger, 1863**

*Polyrhachis rupicapra* Roger, 1863: 154. TL: [Ceylon] Sri Lanka [Syntype: ZMHB?].

**Distribution.** Wet Zone; *Primary literature records*: Ceylon (Roger 1863: 154), Ceylon (Forel 1893a: 33), Bandarawela (Emery 1901: 122); *Secondary literature records*: Ceylon (Emery 1896: 779), Ceylon (Emery 1925: 196), Sri Lanka (Dias 2002: 19), Sri Lanka (Dias 2006a: 51), Sri Lanka (Li-Zhong 2006: 271), Sri Lanka (Rajan et al. 2006: 173), Sri Lanka (Dias et al. 2012: 19); *AntWeb records*: Sri Lanka: CASENT 0906783 (AntWeb 2020).

***Polyrhachis saevissima argentea* Mayr, 1862**

*Polyrhachis argenteus* Mayr, 1862: 682. TL: Philippines [Syntype: NHMW]. [Images of CASENT 0915827 syntype worker examined].

**Distribution.** Wet Zone; *Primary literature records*: Kandy (Emery 1893a: 254).

***Polyrhachis scissa* (Roger, 1862)**

*Hemioptica scissa* Roger, 1862: 240. TL: [Ceylon] Sri Lanka [Lectotype: ZMHB]. [Images of FOCOL 2556, 2557 lectotype and paralectotype workers examined].

**Distribution.** Wet, Dry and Intermediate Zones; *Primary literature records*: Ceylon (Roger 1862: 240), Kandy (Emery 1893a: 255), Ceylon (Forel 1893a: 27), Ceylon (Emery 1901: 122), Puwakpitiya, Galle (Forel 1908: 13), Ceylon (Forel 1909: 402), Seenigoda (Forel 1911a: 228), Anuradhapura, Yala National Park (Dorow and Kohout 1995: 101), Sri Lanka (Dias 2014: 129), Anuradhapura, Colombo, Galle, Gampaha, Kurunegala, Polonnaruwa, Ratnapura (Dias and Rajapaksa 2016: 35); *Secondary literature records*: Ceylon (Emery 1896: 780), Ceylon (Emery 1925: 210), Ceylon (Donisthorpe 1942b: 461), Ceylon (Chapman and Capco 1951: 256), Sri Lanka (Dias 2002: 19), Sri Lanka (Dias 2006a: 50), Sri Lanka (Dias et al. 2012: 19); *AntWeb records*: Sri Lanka: FOCOL 2556, 2557 (AntWeb 2020).

***Polyrhachis sophocles* Forel, 1908**

*Polyrhachis sophocles* Forel, 1908: 10. TL: [Ceylon] Sri Lanka [Holotype: MHNG]. [Images of CASENT 0910898 holotype worker examined].

**Distribution.***Primary literature records*: Ceylon (Forel 1908: 10); *Secondary literature records*: Ceylon (Emery 1925: 196), Sri Lanka (Dias 2002: 19), Sri Lanka (Dias 2006a: 51), Sri Lanka (Dias et al. 2012: 19); *AntWeb records*: Sri Lanka: CASENT 0910898 (AntWeb 2020).

***Polyrhachis thrinax* Roger, 1863**

*Polyrhachis thrinax* Roger, 1863: 152. TL: Nadungayam, Malabar: India; [Ceylon] Sri Lanka [Syntypes: NHMUK, ZMHB]. [Images of CASENT 0903324, FOCOL 2525, 2526 syntype workers examined].

**Distribution.** Wet Zone; *Primary literature records*: Ceylon (Roger 1863: 152), Nawalapitiya, Wacwell (Emery 1893a: 254), Ceylon (Forel 1893a: 28), Ceylon (Emery 1901: 122), Puwakpitiya, Galle (Forel 1908: 9), Ceylon (Forel 1909: 401), Peradeniya (Forel 1913a: 135), Ceylon (Brown 1959: 164), Sinharaja Forest Reserve (Gunawardene et al. 2008: 80); *Secondary literature records*: Ceylon (Emery 1896: 776), Ceylon (Emery 1925: 183), Ceylon (Donisthorpe 1942b: 460), Ceylon (Chapman and Capco 1951: 302), Sri Lanka (Ali 1992: 9), Sri Lanka (Tiwari 1999: 79), Sri Lanka (Tiwari et al. 1999: 273), Sri Lanka (Dias 2002: 19), Sri Lanka (Jaitrong and Nabhitabhata 2005: 40), Sri Lanka (Dias 2006a: 51), Sri Lanka (Dias et al. 2012: 19); *AntWeb records*: Sri Lanka: FOCOL 2525, 2526, SAM-HYM-C 005302 (AntWeb 2020).

***Polyrhachis thrinax lancearia* Forel, 1893**

Polyrhachis thrinax var. lancearius Forel, 1893a: 19. TL: [Kanara], Karnataka: India [Syntype: MHNG]. [Images of CASENT 0910799 syntype worker examined].

**Distribution.** Sri Lanka (Simon Robson personal collection).

***Polyrhachis tibialis* Smith F, 1858**

*Polyrhachis tibialis* Smith F, 1858: 63. TL: Myanmar [Syntype: NHMUK?].

**Distribution.***Primary literature records*: Sri Lanka (Ali 1992: 9); *Secondary literature records*: Sri Lanka (Tiwari et al. 1999: 273), Sri Lanka (Dias 2002: 19), Sri Lanka (Sheela 2008a: 19), Sri Lanka (Dias et al. 2012: 19).

***Polyrhachis tibialis parsis* Emery, 1900**

Polyrhachis tibialis var. parsis Emery, 1900: 717. TL: [Kanara], Karnataka: India [Syntype: MSNG]. [Images of CASENT 0905637 syntype worker examined].

**Distribution.** Wet Zone; *Primary literature records*: Puwakpitiya (Forel 1908: 9), Ceylon (Forel 1909: 401), Ceylon (Forel 1911a: 228); *Secondary literature records*: Ceylon (Emery 1925: 196), Ceylon (Donisthorpe 1942b: 460).

***Polyrhachis tibialis pectita* Santschi, 1928**

Polyrhachis tibialis var. pectita Santschi, 1928: 136. TL: Paradeniya: [Ceylon] Sri Lanka [Syntype: NHMB]. [Images of CASENT 0912159 syntype worker examined].

**Distribution.** Wet Zone; *Primary literature records*: Paradeniya (Santschi 1928: 136); *Secondary literature records*: Sri Lanka (Dias 2006a: 51); *AntWeb records*: Sri Lanka: CASENT 0912159 (AntWeb 2020).

***Polyrhachis tubericeps* Forel, 1893**

*Polyrhachis tubericeps* Forel, 1893a: 33. TL: [Benares] Banaras: India [Syntype: MHNG]. [Images of CASENT 0910899 syntype worker examined].

**Distribution.***Primary literature records*: Sri Lanka (Dias 2002: 19).

***Polyrhachis wroughtonii* Forel, 1894**

*Polyrhachis wroughtonii* Forel, 1894: 398. TL: [Kanara], Karnataka: India [Syntypes: MHNG, NHMUK]. [Images of CASENT 0903351, CASENT 0910854 syntype workers examined].

**Distribution.** Sri Lanka (Simon Robson personal collection).

***Polyrhachis xanthippe* Forel, 1911**

*Polyrhachis xanthippe* Forel, 1911c: 61. TL: Namunukula: [Ceylon] Sri Lanka [Syntypes: MHNG, NHMUK]. [Images of CASENT 0903385, CASENT 0910900 syntype workers examined].

**Distribution.** Intermediate Zone; *Primary literature records*: Namunukula (Forel 1911c: 62); *Secondary literature records*: Ceylon (Emery 1925: 197), Ceylon (Chapman and Capco 1951: 300), Sri Lanka (Dias 2002: 19), Sri Lanka (Dias 2006a: 51), Sri Lanka (Dias et al. 2012: 19); *AntWeb records*: Namunukula: CASENT 0903385, CASENT 0910900 (AntWeb 2020).

***Polyrhachis yerburyi* Forel, 1893**

*Polyrhachis yerburyi* Forel, 1893a: 29. TL: [Ceylon] Sri Lanka [Syntype: MHNG]. [Images of CASENT 0910933 syntype worker examined].

**Distribution.** Wet Zone; *Primary literature records*: Ceylon (Forel 1893a: 29), Sinharaja Forest Reserve (Gunawardene et al. 2008: 80); *Secondary literature records*: Ceylon (Emery 1896: 777), Ceylon (Emery 1925: 203), Ceylon (Chapman and Capco 1951: 279), Sri Lanka (Dias 2002: 19), Sri Lanka (Dias 2006a: 51), Sri Lanka (Dias et al. 2012: 19); *AntWeb records*: Sri Lanka: CASENT 0910933 (AntWeb 2020).

#### *Prenolepis*: 1 species

***Prenolepis naoroji* Forel, 1902**

*Prenolepis naoroji* Forel, 1902a: 290. TL: North West Provinces: India [Syntype: MHNG]. [Images of CASENT 0911042 syntype worker examined].

**Distribution.** Wet and Intermediate Zones; *Primary literature records*: Sri Lanka (Dias et al. 2012: 19), Kirikanda Forest (Dias et al. 2013: 64), Egodayagama (Dias and Peiris 2015a: 5), Colombo, Galle, Gampaha, Ratnapura (Dias and Rajapaksa 2016: 35).

#### *Pseudolasius*: 1 species

***Pseudolasius isabellae* Forel, 1908**

*Pseudolasius isabellae* Forel, 1908: 4. TL: Puwakpitiya: [Ceylon] Sri Lanka [Syntype: MHNG]. [Images of CASENT 0910976 syntype queen examined].

**Distribution.** Wet and Dry Zones; *Primary literature records*: Puwakpitiya (Forel 1908: 5), Ceylon (Forel 1909: 396), Pohoranwewa, Dambulla (Dias and Kosgamage 2012: 62); *Secondary literature records*: Ceylon (Emery 1925: 215), Ceylon (Chapman and Capco 1951: 204), Sri Lanka (Dias 2002: 18), Sri Lanka (Dias 2006a: 51), Sri Lanka (Dias et al. 2012: 19); *AntWeb records*: Puwakpitiya: CASENT 0910976 (AntWeb 2020).

### LEPTANILLINAE

#### *Leptanilla*: 1 species

***Leptanilla besucheti* Baroni Urbani, 1977**

*Leptanilla besucheti* Baroni Urbani, 1977b: 448. TL: Pidurutalagala: Sri Lanka [Holotype: MHNG]. [Images of CASENT 0911183 holotype worker examined].

**Distribution.** Wet Zone; *Primary literature records*: Pidurutalagala (Baroni Urbani 1977a: 72), Pidurutalagala (Baroni Urbani 1977b: 449), Sri Lanka (Dias 2014: 149); *Secondary literature records*: Sri Lanka (Dias 2006a: 52), Sri Lanka (Bharti and Kumar 2012a: 624), Sri Lanka (Dias et al. 2012: 18); *AntWeb records*: Pidurutalagala: CASENT 0902779, CASENT 0911183 (AntWeb 2020).

#### *Protanilla*: 1 species

***Protanilla schoedli* Baroni Urbani and De Andrade, 2006**

*Protanilla schoedli* Baroni Urbani and De Andrade, 2006: 45. TL: Uva, Inginirgala [Inginiyagala] Sri Lanka [Holotype: MHNG]. [Images of CASENT 0911228 holotype queen examined].

**Distribution.** Wet and Dry Zones; *Primary literature records*: Uva, Inginirgala (Baroni Urbani and De Andrade 2006: 45), Pompekelle, Gilimale Forest Reserve, Sinharaja Forest Reserve, Anuradhapura Sanctuary (Dias et al. 2019: 163); *Secondary literature records*: Sri Lanka (Terayama 2009: 126); *AntWeb records*: Uva, Inginirgala: CASENT 0911228 (AntWeb 2020).

#### *Yavnella*: 1 species

***Yavnella* sp.**

**Distribution.** Wet Zone; *Primary literature records*: Sri Lanka (Boudinot 2015: 31); *AntWeb records*: Victoria, Randenigala Rantembe Sanctuary: CASENT 0106366, CASENT 0106375–0106381 (AntWeb 2020).

### MYRMICINAE

#### *Acanthomyrmex*: 1 species

***Acanthomyrmex luciolae* Emery, 1893**

*Acanthomyrmex luciolae* Emery, 1893b: cclxxvi. TL: Kandy: [Ceylon] Sri Lanka [Syntypes: MSNG]. [Images of CASENT 0904701, 0904702 syntype workers examined].

**Distribution.** Wet Zone; *Primary literature records*: Kandy (Emery 1893a: 245), Ceylon (Emery 1893b: cclxxvi), Kandy (Moffett 1986: 76), Sinharaja Forest Reserve (Gunawardene et al. 2008: 81); *Secondary literature records*: Kandy (Forel 1903a: 696), Ceylon (Chapman and Capco 1951: 115), Sri Lanka (Dias 2002: 18), Sri Lanka (Dias 2006a: 51), Sri Lanka (Dias et al. 2012: 15); *AntWeb records*: Gilimale, Kandy: CASENT 0281791, CASENT 0904701, 0904702 (AntWeb 2020).

#### *Anillomyrma*: 1 species

***Anillomyrma decamera* (Emery, 1901)**

*Monomorium decamerum* Emery, 1901: 117. TL: Anuradhapura: [Ceylon] Sri Lanka [Syntype: MSNG]. [Images of CASENT 0904590 syntype worker examined].

**Distribution.** Dry Zone; *Primary literature records*: Anuradhapura (Emery 1901: 118), Ceylon (Ettershank 1966: 98), Anuradhapura (Eguchi et al. 2010: 33); *Secondary literature records*: Ceylon (Forel 1903a: 687), Ceylon (Emery 1922: 184), Ceylon (Chapman and Capco 1951: 161), Sri Lanka (Bolton 1987: 274), Sri Lanka (Dias 2002: 18), Sri Lanka (Dias 2006a: 51), Sri Lanka (Terayama 2009: 151), Sri Lanka (Dias et al. 2012: 15); *AntWeb records*: Sri Lanka: CASENT 0904590 (AntWeb 2020).

#### *Aphaenogaster*: 1 species

***Aphaenogaster beccarii* Emery, 1887**

*Aphaenogaster beccarii* Emery, 1887b: 456. TL: Sumatra: Indonesia [Syntypes: MSNG]. [Images of CASENT 0904186 syntype worker examined].

**Distribution.***Primary literature records*: Sri Lanka (Dias 2006a: 51).

#### *Calyptomyrmex*: 3 species

***Calyptomyrmex singalensis* Baroni Urbani, 1975**

*Calyptomyrmex singalensis* Baroni Urbani, 1975: 402. TL: Kantale: [Ceylon Eastern] Sri Lanka [Holotype: MHNG]. [Images of CASENT 0911132 holotype worker examined].

**Distribution.** Dry Zone; *Primary literature records*: Kantale, Inginiyagala, Uva (Baroni Urbani 1975: 403); *Secondary literature records*: Sri Lanka (Dias 2006a: 51), Sri Lanka (Dias et al. 2012: 15); *AntWeb records*: Kantale: CASENT 0900993, CASENT 0911132 (AntWeb 2020).

***Calyptomyrmex tamil* Baroni Urbani, 1975**

*Calyptomyrmex tamil* Baroni Urbani, 1975: 398. TL: 15 Uva near Wellawaya [Ceylon] Sri Lanka [Holotype: MHNG]. [Images of CASENT 0911133 holotype worker examined].

**Distribution.** Dry Zone; *Primary literature records*: Wellawaya, Uva (Baroni Urbani 1975: 401); *Secondary literature records*: Sri Lanka (Dias 2006a: 51), Sri Lanka (Dias et al. 2012: 15); *AntWeb records*: Uva near Wellawaya: CASENT 0911133 (AntWeb 2020).

***Calyptomyrmex vedda* Baroni Urbani, 1975**

*Calyptomyrmex vedda* Baroni Urbani, 1975: 404. TL: 63 Inginiyagala, Uva: [Ceylon] Sri Lanka [Holotype: MHNG]. [Images of CASENT 0911134 holotype worker examined].

**Distribution.** Dry Zone; *Primary literature records*: Inginiyagala, Uva (Baroni Urbani 1975: 405); *Secondary literature records*: Sri Lanka (Dias 2006a: 51); Sri Lanka (Dias et al. 2012: 15); *AntWeb records*: Uva Inginiyagala: CASENT 0911134 (AntWeb 2020).

#### *Cardiocondyla*: 5 species

***Cardiocondyla emeryi* Forel, 1881**

*Cardiocondyla emeryi* Forel, 1881: 5. TL: St. Thomas Island, Virgin Islands [Syntypes: MHNG, SIZK]. [Images of CASENT 0916973 syntype worker examined].

**Distribution.** Wet Zone; *Primary literature records*: Bandarawela (Seifert 2003: 277); *Secondary literature records*: Sri Lanka (Mohammadi et al. 2012: 849), Sri Lanka (Wetterer 2012a: 16).

***Cardiocondyla itsukii* Seifert, Okita & Heinze, 2017**

*Cardiocondyla itsukii* Siefert, Okita & Heinze, 2017: 339. TL: Shizuoka, Iwata-shi: Japan [Holotype: SMNG]. [Images of ANTWEB 1038017 holotype worker examined].

**Distribution.** Wet Zone; *Primary literature records*: Bandarawela, Nuwara Eliya (Seifert et al. 2017: 341).

***Cardiocondyla kagutsuchi* Terayama, 1999**

*Cardiocondyla kagutsuchi* Terayama, 1999: 100. TL: Omoto-dake, Ishigaki-jima, Okinawa: Japan [Holotype: MNHAH]. [Images of ANTWEB 1041248 paratype worker examined].

**Distribution.** Wet Zone; *Primary literature records*: Bandarawela, Nuwara Eliya, Labukelle (Seifert 2003: 252), Sri Lanka (Seifert 2008: 45).

***Cardiocondyla minutior* Forel, 1899**

Cardiocondyla nuda var. minutior Forel, 1899: 120. TL: Molokai Mountains, Hawaiian Islands: United States [Syntypes: MHNG, NHMUK]. [Images of CASENT 0908344 syntype worker examined].

**Distribution.** Wet and Intermediate Zones; *Primary literature records*: Nuwara Eliya, Labukelle, Gavarammana (Seifert 2003: 283); *Secondary literature records*: Sri Lanka (Wetterer 2014: 568).

***Cardiocondyla wroughtonii* (Forel, 1890)**

*Emeryia wroughtonii* Forel, 1890: cxi. TL: [Poona] Pune: India [Syntypes: NHMUK, MHNG]. [Images of CASENT 0908349, 0908350, CASENT 0901753 syntype workers and ergatoid male examined].

**Distribution.** Wet Zone; *Primary literature records*: Peradeniya, Nawalapitiya (Bolton 1982: 318), Peradeniya (Seifert 2003: 270), Sinharaja Forest Reserve (Gunawardene et al. 2008: 81).

#### *Carebara*: 12 species/subspecies

***Carebara affinis* (Jerdon, 1851)**

*Oecodoma affinis* Jerdon, 1851: 110. TL: Malabar, Kerala: India [Type: UNK].

**Distribution.** Intermediate Zone; *Primary literature records*: Ceylon (Bingham 1903: 165), Nalanda (Forel 1913b: 662); *Secondary literature records*: Ceylon (Forel 1903a: 691), Sri Lanka (Ali 1992: 5), Sri Lanka (Tiwari 1999: 60), Sri Lanka (Tiwari et al. 1999: 262), Sri Lanka (Mathew and Tiwari 2000: 331), Sri Lanka (Dias 2002: 17), Sri Lanka (Tiwari et al. 2004: 612), Sri Lanka (Ghosh et al. 2006: 389), Sri Lanka (Rajan et al. 2006: 182), Sri Lanka (Tak et al. 2007: 129).

***Carebara bruni* (Forel, 1913)**

*Oligomyrmex bruni* Forel, 1913a: 61. TL: Peradeniya: [Ceylon] Sri Lanka [Syntypes: MHNG, ZMHB]. [Images of CASENT 0908901, 0908902, FOCOL 1926 syntype workers examined].

**Distribution.** Wet Zone; *Primary literature records*: Peradeniya, Botanical Garden (Forel 1913a: 63); *Secondary literature records*: Ceylon (Chapman and Capco 1951: 156), Sri Lanka (Dias 2002: 17), Sri Lanka (Dias 2006a: 51), Sri Lanka (Dias et al. 2012: 16); *AntWeb records*: Peradeniya: CASENT 0908901, 0908902, FOCOL 1926 (AntWeb 2020).

***Carebara butteli* (Forel, 1913)**

*Pheidologeton butteli* Forel, 1913a: 56. TL: Peradeniya: [Ceylon] Sri Lanka [Syntypes: MHNG, ZMHB]. [Images of CASENT 0908888, 0908889, FOCOL 1907–1912 syntype workers examined].

**Distribution.** Wet Zone; *Primary literature records*: Peradeniya, Experiment Station (Forel 1913a: 58), Peradeniya, Experiment Station (Fischer et al. 2015: 85); *Secondary literature records*: Ceylon (Wheeler 1936: 220), Ceylon (Chapman and Capco 1951: 155), Ceylon (Ettershank 1966: 123), Sri Lanka (Dias 2002: 17), Sri Lanka (Dias 2006a: 51), Sri Lanka (Dias et al. 2012: 16); *AntWeb records*: Sri Lanka: CASENT 0908888, 0908889, FOCOL 1907–1912 (AntWeb 2020).

***Carebara ceylonensis* (Forel, 1911)**

Pheidologeton pygmaeus subsp. ceylonensis Forel, 1911a: 220. TL: Dividosgala, Peradeniya: [Ceylon] Sri Lanka [Syntypes: MHNG]. [Images of CASENT 0908950 syntype queen examined].

**Distribution.** Wet Zone; *Primary literature records*: Dividosgala, Peradeniya (Forel 1911a: 221), Ambalagoda (Forel 1912c: 54); *Secondary literature records*: Ceylon (Chapman and Capco 1951: 154), Ceylon (Ettershank 1966: 118), Sri Lanka (Dias 2002: 17), Sri Lanka (Yamane 2003: 83), Sri Lanka (Dias 2006a: 52), Sri Lanka (Dias et al. 2012: 16); *AntWeb records*: Dividosgala: CASENT 0908950 (AntWeb 2020).

***Carebara deponens* (Walker, 1859)**

*Crematogaster deponens* Walker, 1859: 374. TL: [Ceylon] Sri Lanka [Holotype: NHMUK]. [Images of CASENT 0902387 holotype queen examined].

**Distribution.***Primary literature records*: Ceylon (Walker 1859: 374); *Secondary literature records*: Ceylon (Emery 1922: 158), Ceylon (Chapman and Capco 1951: 95), Sri Lanka (Dias 2002: 18), Sri Lanka (Dias 2006a: 51), Sri Lanka (Dias et al. 2012: 16); *AntWeb records*: Sri Lanka: CASENT 0902387 (AntWeb 2020).

***Carebara diversa* (Jerdon, 1851)**

*Oecodoma diversa* Jerdon, 1851: 109. TL: Wynaad, Kerala: India [Type: UNK].

**Distribution.** Wet Zone; *Primary literature records*: Colombo, Ratgama Kellie (Emery 1893a: 243), Kelaniya, Ratnapura (Dias 2006a: 45), Nawalapitiya (Amarasinghe 2010: 12), Sri Lanka (Dias 2014: 183), Indikada Mukalana Forest Reserve (Udayakantha and Dias 2018: 72); *Secondary literature records*: Sri Lanka (Dias 2002: 17), Sri Lanka (Yamane 2003: 77), Sri Lanka (Dias 2006b: 8), Sri Lanka (Li-Zhong 2006: 270), Sri Lanka (Dias et al. 2012: 16).

***Carebara diversa taprobanae* (Smith F, 1858)**

*Pheidole taprobanae* Smith F, 1858: 175. TL: [Ceylon] Sri Lanka [Holotype: NHMUK]. [Images of CASENT 0900735 holotype queen examined].

**Distribution.** Wet Zone; *Primary literature records*: Ceylon (Smith, F. 1858: 175), Bentota (Emery 1901: 120), Ceylon (Forel 1909: 394), Seenigoda (Forel 1911a: 220); *Secondary literature records*: Ceylon (Forel 1903a: 691), Seenigoda (Forel 1913a: 55), Ceylon (Chapman and Capco 1951: 95), Sri Lanka (Dias 2002: 17), Sri Lanka (Yamane 2003: 83), Sri Lanka (Dias 2006a: 51), Sri Lanka (Dias et al. 2012: 16); *AntWeb records*: Sri Lanka: CASENT 0908918, 0908919 (AntWeb 2020).

***Carebara escherichi* (Forel, 1911)**

*Paedalgus escherichi* Forel, 1911a: 218. TL: Peradenyia: Ceylon Sri Lanka [Syntypes: MHNG, NHMUK, SIZK]. [Images of CASENT 0902375, CASENT 0908921, CASENT 0917025 syntype workers examined].

**Distribution.** Wet Zone; *Primary literature records*: Peradeniya (Forel 1911a: 219), Peradeniya (Bolton and Belshaw 1993: 185), Sri Lanka (Dias 2014: 166); *Secondary literature records*: Ceylon (Wheeler 1936: 220), Ceylon (Chapman and Capco 1951: 158), Ceylon (Ettershank 1966: 129); Sri Lanka (Dias 2002: 17); Sri Lanka (Dias 2006a: 51), Sri Lanka (Dias et al. 2012: 16); *AntWeb records*: Peradeniya: CASENT 0902375, CASENT 0908921, CASENT 0917025 (AntWeb 2020).

***Carebara nana* (Roger, 1863)**

*Pheidologeton nanus* Roger, 1863: 191. TL: [Ceylon] Sri Lanka [Syntype: ZMHB].

**Distribution.** Wet and Intermediate Zones; *Primary literature records*: Ceylon (Roger 1863: 193), Kandy (Emery 1893a: 243), Nalanda (Emery 1901: 120), Ceylon (Forel 1908: 3), Peradeniya (Forel 1911a: 220); *Secondary literature records*: Ceylon (Forel 1903a: 691), Ceylon (Chapman and Capco 1951: 160), Ceylon (Ettershank 1966: 119), Sri Lanka (Dias 2002: 18), Sri Lanka (Yamane 2003: 83), Sri Lanka (Ghosh et al. 2006: 390), Sri Lanka (Li-Zhong 2006: 270); *AntWeb records*: Sri Lanka: CASENT 0906202, CASENT 0906213 (AntWeb 2020).

***Carebara pygmaea* (Emery, 1887)**

*Pheidologeton pygmaeus* Emery, 1887c: 465. TL: Ternate, Acqui Conora, Maluku: Indonesia [Syntypes: MSNG]. [Images of CASENT 0904653, 0904654 syntype workers examined].

**Distribution.** Wet Zone; *Primary literature records*: Ambalangoda (Forel 1912c: 54), Sinharaja Forest Reserve (Gunawardene et al. 2012: 85), Peradeniya (Fischer et al. 2014: 72); *Secondary literature records*: Ceylon (Chapman and Capco 1951: 154), Ceylon (Ettershank 1966: 118), Sri Lanka (Yamane 2003: 78), Sri Lanka (Dias 2006a: 52), Sri Lanka (Dias et al. 2012: 16); *AntWeb records*: Dividosgala: CASENT 0908951, CASENT 0908952 (AntWeb 2020).

***Carebara silenus* (Smith F, 1858)**

*Pheidole silenus* Smith F, 1858: 176. TL: Singapore [Syntypes: NHMUK]. [Images of CASENT 0900737, 0900738 syntype workers examined].

**Distribution.***Primary literature records*: Ceylon (Forel 1903a: 691); *Secondary literature records*: Ceylon (Chapman and Capco 1951: 160), Sri Lanka (Dias 2002: 18), Sri Lanka (Yamane 2003: 79).

***Carebara sinhala* Fischer, Azorsa & Fisher, 2014**

*Carebara sinhala* Fischer, Azorsa & Fisher, 2014: 71. TL: Sri Lanka [Syntype: MHNG].

**Distribution.** Wet Zone; *Primary literature records*: Peradeniya (Forel 1911a: 220); *Secondary literature records*: Ceylon (Wheeler 1936: 220), Ceylon (Chapman and Capco 1951: 158), Ceylon (Ettershank 1966: 119), Sri Lanka (Dias 2002: 18), Sri Lanka (Dias 2006a: 51), Sri Lanka (Dias et al. 2012: 16), Ceylon (Fischer et al. 2014: 71).

#### *Cataulacus*: 4 species

***Cataulacus granulatus* (Latreille, 1802)**

*Formica granulata* Latreille, 1802: 275. TL: Singapore [Holotype: OUMNH].

**Distribution.** Wet Zone; *Primary literature records*: Kandy (Bolton 1974a: 65).

***Cataulacus latus* Forel, 1891**

*Cataulacus latus* Forel, 1891: 144. TL: [Kanara], Karnataka, [Poona] Pune, [Pooree] Puri [Syntypes: MHNG, ZSM]. [Images of CASENT 0909239, 0909240, FOCOL 0660–0662 syntype workers, queens and male examined].

**Distribution.** Wet Zone; *Primary literature records*: Sinharaja Forest Reserve (Gunawardene et al. 2008: 81).

***Cataulacus simoni* Emery, 1893**

*Cataulacus simoni* Emery, 1893a: 248. TL: Kandy: [Ceylon] Sri Lanka [Syntype: MSNG]. [Images of CASENT 0904879 syntype worker examined].

**Distribution.** Wet and Dry Zones; *Primary literature records*: Colombo, Kandy (Emery 1893a: 248), Galle (Forel 1908: 2), Ceylon (Forel 1909: 393), Laxapathiya, Peradenya, Yakkala, Polonnaruwa (Bolton 1974a: 73), Sinharaja Forest Reserve (Gunawardene et al. 2008: 81); *Secondary literature records*: Colombo, Kandy (Forel 1903a: 706), Ceylon (Chapman and Capco 1951: 86), Sri Lanka (Mathew and Tiwari 2000: 322), Sri Lanka (Dias 2002: 18), Sri Lanka (Dias 2006a: 51), Sri Lanka (Dias et al. 2012: 15); *AntWeb records*: Kandy, Coconut Research Institute, Lunuwila: CASENT 0280797, CASENT 0904879 (AntWeb 2020).

***Cataulacus taprobanae* Smith F, 1853**

*Cataulacus taprobanae* Smith F, 1853: 225. TL: [Ceylon] Sri Lanka [Holotype: NHMUK]. [Images of CASENT 0900254 holotype worker examined].

**Distribution.** Wet and Dry Zones; *Primary literature records*: Ceylon (Smith, F. 1853: 225), Kandy, Wackwella (Emery 1893a: 248), Ceylon (Emery 1901: 121), Puwakpitiya (Forel 1908: 2), Ceylon (Forel 1909: 393), Dividsogala, Peradeniya (Forel 1911a: 226), Seenigoda (Forel 1913a: 83), Kandy, Polonnaruwa, Gilimale, Belihuloya (Bolton 1974a: 84), Sri Lanka Cashew Corporation-West, western Sri Lanka (Rickson and Rickson 1998: 843), Anuradhapura, Polonnaruwa (Dias and Kosgamage 2012: 62), Sri Lanka (Dias 2014: 168), Anuradhapura, Colombo, Galle, Gampaha, Polonnaruwa, Puttalam, Ratnapura (Dias and Rajapaksa 2016: 35); *Secondary literature records*: Ceylon (Forel 1903a: 706), Ceylon (Chapman and Capco 1951: 86), Sri Lanka (Ali 1992: 2), Sri Lanka (Dias 2002: 18), Sri Lanka (Mathew and Tiwari 2000: 322), Sri Lanka (Mathew 2003: 199), Sri Lanka (Dias 2006a: 51), Sri Lanka (Rajan et al. 2006: 174), Sri Lanka (Dias et al. 2012: 15); *AntWeb records*: Sri Lanka: CASENT 0900254 (AntWeb 2020).

#### *Crematogaster*: 21 species/subspecies

***Crematogaster anthracina* Smith F, 1857**

*Crematogaster anthracinus* Smith F, 1857: 75. TL: Singapore [Syntype: OUMNH]. [Images of CASENT 0901434 syntype worker examined].

**Distribution.***Primary literature records*: Sri Lanka (Dias 2002: 18) ; *Secondary literature records*: Sri Lanka (Mathew and Tiwari 2000: 324), Sri Lanka (Dias 2006a: 51), Sri Lanka (Rajan et al. 2006: 175), Sri Lanka (Hosoishi and Ogata 2009: 45), Sri Lanka (Dias et al. 2012: 15).

***Crematogaster apicalis* Motschoulsky, 1863**

*Crematogaster apicalis* Motschoulsky, 1863: 20. TL: [Ceylon] Sri Lanka [Type: UNK].

**Distribution.** Wet Zone; *Primary literature records*: Ceylon (Motschoulsky 1863: 20); *Secondary literature records*: Ceylon (Emery 1922: 149), Ceylon (Chapman and Capco 1951: 86), Sri Lanka (Dias 2002: 18), Nuwara Eliya Mountains (Hosoishi and Ogata 2009: 45), Sri Lanka (Dias 2006a: 51), Sri Lanka (Dias et al. 2012: 15).

***Crematogaster biroi* Mayr, 1897**

*Crematogaster biroi* Mayr, 1897: 428. TL: Colombo: [Ceylon] Sri Lanka [Syntype: HNHM]. [Images of CASENT 0916605 syntype worker examined].

**Distribution.** Wet, Dry and Intermediate Zones; *Primary literature records*: Colombo (Mayr 1897: 429), Peradeniya (Forel 1911a: 223), Peradenyia (Menozzi 1935: 106), Pulliyarahandiya (Dias and Kosgamage 2012: 62), Sri Lanka (Dias 2014: 170), Mawathagama, Kurunegala (Dias and Peiris 2015b: 26), Anuradhapura, Colombo, Galle, Gampaha, Kurunegala, Polonnaruwa, Ratnapura (Dias and Rajapaksa 2016: 35), Colombo (Hosoishi and Ogata 2016: 577); *Secondary literature records*: Ceylon (Forel 1903a: 682), Ceylon (Emery 1922: 131), Colombo (Wheeler 1930: 66), Ceylon (Wheeler 1935: 21), Ceylon (Chapman and Capco 1951: 96), Sri Lanka (Tiwari 1999: 49), Sri Lanka (Dias 2002: 18), Sri Lanka (Mathew and Tiwari 2000: 324), Sri Lanka (Tiwari et al. 2003: 487), Sri Lanka (Dias 2006a: 51), Sri Lanka (Ghosh et al. 2006: 387), Sri Lanka (Li-Zhong 2006: 265), Colombo, Peradenyia (Hosoishi and Ogata 2009: 64), Sri Lanka (Dias et al. 2012: 15); *AntWeb records*: Colombo, Kandy: CASENT 0907505, CASENT 0914303, CASENT 0916605 (AntWeb 2020).

***Crematogaster brunnea* Smith F, 1857**

*Crematogaster brunneus* Smith F, 1857: 75. TL: Sarawak: Malaysia [Syntypes: NHMUK, OUMNH]. [Images of CASENT 0901435, CASENT 0902122 syntype workers examined].

**Distribution.** Intermediate Zone; *Primary literature records*: Sri Lanka (Dias 2002: 18), Mawathagama, Kurunegala (Dias and Peiris 2015b: 26); *Secondary literature records*: Sri Lanka (Hosoishi and Ogata 2009: 46).

***Crematogaster brunnea nicevillei* Emery, 1922**

*Crematogaster brunnea nicevillei* Emery, 1922: 149. TL: [Calcutta] Kolkata, West Bengal: India [Syntype: MHNG]. [Images of CASENT 0908565 syntype worker examined].

**Distribution.** Wet Zone; *Primary literature records*: Colombo (Forel 1912c: 57).

***Crematogaster brunnea rabula* Forel, 1902**

Crematogaster subnuda r. rabula Forel, 1902b: 207. TL: [Bombay] Mumbai, [Poona] Pune: India [Syntypes: MHNG, NHMUK]. [Images of CASENT 0908568 syntype worker examined].

**Distribution.** Wet and Dry Zones; *Primary literature records*: Trincomalee, Ambalangoda (Forel 1909: 394), Seenigoda (Forel 1913a: 75); *Secondary literature records*: Ceylon (Chapman and Capco 1951: 87).

***Crematogaster brunnescens* Motschoulsky, 1863**

*Crematogaster brunnescens* Motschoulsky, 1863: 20. TL: [Ceylon] Sri Lanka [Type: UNK].

**Distribution.** Wet Zone; *Primary literature records*: Ceylon (Motschoulsky 1863: 20); *Secondary literature records*: Ceylon (Emery 1922: 150), Ceylon (Chapman and Capco 1951: 89), Sri Lanka (Dias 2002: 18), Sri Lanka (Dias 2006a: 51), Colombo (Hosoishi and Ogata 2009: 48), Sri Lanka (Dias et al. 2012: 15).

***Crematogaster consternens* (Walker, 1859)**

*Myrmica consternens* Walker, 1859: 374. TL: [Ceylon] Sri Lanka [Holotype: NHMUK]. [Images of CASENT 0902127 holotype male examined].

**Distribution.***Primary literature records*: Ceylon (Walker 1859: 374); *Secondary literature records*: Ceylon (Chapman and Capco 1951: 125), Sri Lanka (Dias 2002: 18), Sri Lanka (Dias 2006a: 51).

***Crematogaster contemta* Mayr, 1879**

*Crematogaster contemta* Mayr, 1879: 685. TL: [Calcutta] Kolkata, West Bengal: India [Syntype: NHMW]. [Images of CASENT 0919680 syntype worker examined].

**Distribution.** Dry Zone; *Primary literature records*: Ceylon (Emery 1922: 150), Ceylon (Chapman and Capco 1951: 87); Puttalam (Dias and Rajapaksa 2016: 35); *Secondary literature records*: Sri Lanka (Tiwari 1997: 445), Sri Lanka (Tiwari et al. 1999: 251), Sri Lanka (Tak and Rathore 2004b: 87), Sri Lanka (Tak 2008: 16), Sri Lanka (Hosoishi and Ogata 2009: 46), Sri Lanka (Tak and Kazmi 2011: 42).

***Crematogaster desecta* Forel, 1911**

*Crematogaster desecta* Forel, 1911c: 27. TL: Namunukula: [Ceylon] Sri Lanka [Syntype: MHNG]. [Images of CASENT 0908573 syntype worker examined].

**Distribution.** Intermediate Zone; *Primary literature records*: Namunukula (Forel 1911c: 28); *Secondary literature records*: Ceylon (Emery 1922: 150), Ceylon (Weber 1943: 343), Ceylon (Chapman and Capco 1951: 89), Sri Lanka (Dias 2002: 18), Namunukula (Hosoishi and Ogata 2009: 48); *AntWeb records*: Namunukula: CASENT 0908573 (AntWeb 2020).

***Crematogaster dohrni* Mayr, 1879**

*Crematogaster dohrni* Mayr, 1879: 682. TL: [Ceylon] Sri Lanka [Syntype: NHMW]. [Images of CASENT 0919682 syntype worker examined].

**Distribution.** Wet and Dry Zones; *Primary literature records*: Ceylon (Mayr 1879: 683), Kandy (Emery 1893a: 243), Ceylon (Emery 1901: 120), Kelaniya Valley (Forel 1908: 2), Ceylon (Forel 1909: 394), Dividsogala, Peradeniya (Forel 1911a: 223), Anuradhapura, Polonnaruwa (Dias and Kosgamage 2012: 62), Sri Lanka (Dias 2014: 171), Namalweva, Marawila, Pallama, Madurankuliya (Dias and Peiris 2015a: 6), Sinhapura, Polonnaruwa (Dias and Peiris 2015b: 26), Anuradhapura, Polonnaruwa, Puttalam (Dias and Rajapaksa 2016: 35); *Secondary literature records*: Ceylon (Forel 1903a: 682), Ceylon (Emery 1922: 150), Ceylon (Chapman and Capco 1951: 89), Sri Lanka (Mathew 1984: 307), Sri Lanka (Ali 1992: 2), Sri Lanka (Tiwari 1999: 43), Sri Lanka (Mathew 2000: 350), Sri Lanka (Dias 2002: 18), Sri Lanka (Dias 2006a: 51), Sri Lanka (Dias 2006b: 8), Sri Lanka (Li-Zhong 2006: 265), Sri Lanka (Rajan et al. 2006: 175), Sri Lanka (Hosoishi and Ogata 2009: 49), Sri Lanka (Dias et al. 2012: 15); *AntWeb records*: Kalthota: CASENT 0914077, CASENT 0919682 (AntWeb 2020).

***Crematogaster dohrni gigas* Forel, 1913**

*Crematogaster dohrni gigas* Forel, 1913a: 74. TL: [Huppulama] Maha Iluppalama: [Ceylon] Sri Lanka [Syntypes: MHNG, ZMHB]. [Images of CASENT 0907516, FOCOL 1451 syntype workers examined].

**Distribution.** Dry Zone; *Primary literature records*: Maha Iluppulama (Forel 1913a: 75); *Secondary literature records*: Ceylon (Emery 1922: 150), Ceylon (Chapman and Capco 1951: 89), Maha Iluppulama (Hosoishi and Ogata 2009: 49); *AntWeb records*: Maha Iluppulama: CASENT 0907516, FOCOL 1451 (AntWeb 2020).

***Crematogaster pellens* Walker, 1859**

*Crematogaster pellens* Walker, 1859: 374. TL: [Ceylon] Sri Lanka [Holotype: NHMUK]. [Images of CASENT 0902125 holotype male examined].

**Distribution.***Primary literature records*: Ceylon (Walker 1859: 374); *Secondary literature records*: Ceylon (Emery 1922: 158), Sri Lanka (Dias 2002: 18), Sri Lanka (Dias 2006a: 51), Sri Lanka (Hosoishi and Ogata 2009: 54), Sri Lanka (Dias et al. 2012: 15); *AntWeb records*: Sri Lanka: CASENT 0902125.

***Crematogaster ransonneti* Mayr, 1868**

*Crematogaster ransonneti* Mayr, 1868: 287. TL: [Ceylon] Sri Lanka [Syntypes: NHMUK, NHMW, ZMHB]. [Images of CASENT 0902124, CASENT 0919685, FOCOL 1449, 1450 syntype workers examined].

**Distribution.** Wet Zone; *Primary literature records*: Ceylon (Mayr 1868: 287), Nuwara Eliya (Emery 1893a: 243), Ceylon (Forel 1908: 2); *Secondary literature records*: Ceylon (Forel 1903a: 683), Ceylon (Emery 1922: 151), Ceylon (Chapman and Capco 1951: 93), Sri Lanka (Tiwari 1999: 46), Sri Lanka (Dias 2002: 18), Sri Lanka (Tiwari et al. 2003: 487), Sri Lanka (Dias 2006a: 51), Sri Lanka (Rajan et al. 2006: 175), Sri Lanka (Hosoishi and Ogata 2009: 56), Sri Lanka (Dias et al. 2012: 15); *AntWeb records*: Sri Lanka: CASENT 0902124, CASENT 0919685, FOCOL 1449, 1450 (AntWeb 2020).

***Crematogaster rogenhoferi* Mayr, 1879**

*Crematogaster rogenhoferi* Mayr, 1879: 683. TL: [Mulmein], [Molmein, Brit. Birma] Mawlamyine: Myanmar [Syntypes: NHMW, ZMHB]. [Images of CASENT 0919686, FOCOL 1452 syntype workers examined].

**Distribution.** Wet and Dry Zones; *Primary literature records*: Ceylon (Forel 1903a: 682), Colombo, Galle, Gampaha, Puttalam, Ratnapura (Dias and Rajapaksa 2016: 35); *Secondary literature records*: Ceylon (Wheeler 1921: 535), Ceylon (Emery 1922: 150), Ceylon (Chapman and Capco 1951: 90), Sri Lanka (Tiwari 1999: 43), Sri Lanka (Tiwari et al. 1999: 252), Sri Lanka (Mathew and Tiwari 2000: 327), Sri Lanka (Mathew 2003: 200), Sri Lanka (Tiwari et al. 2003: 488), Sri Lanka (Tiwari et al. 2004: 613), Sri Lanka (Dias 2006a: 51), Sri Lanka (Rajan et al. 2006: 175), Sri Lanka (Hosoishi and Ogata 2009: 56), Sri Lanka (Dias et al. 2012: 15).

***Crematogaster rogenhoferi lutea* Emery, 1893**

Crematogaster rogenhoferi var. lutea Emery, 1893c: 193. TL: Deli, Sumatra: Indonesia [Syntype: MSNG]. [Images of CASENT 0904527 syntype worker examined].

**Distribution.** Wet Zone; *Primary literature records*: Ramboda (Menozzi 1935: 116); *Secondary literature records*: Sri Lanka (Hosoishi and Ogata 2009: 56).

***Crematogaster rogeri* Emery, 1922**

*Crematogaster rogeri* Emery, 1922: 151. TL: [Ceylon] Sri Lanka [Syntype: MSNG?].

**Distribution.***Primary literature records*: Ceylon (Emery 1922: 151); *Secondary literature records*: Sri Lanka (Dias 2006a: 51), Sri Lanka (Hosoishi and Ogata 2009: 57), Sri Lanka (Dias et al. 2012: 15).

***Crematogaster rothneyi* Mayr, 1879**

*Crematogaster rothneyi* Mayr, 1879: 685. TL: [Calcutta] Kolkata, West Bengal: India [Syntype: NHMW]. [Images of CASENT 0919687 syntype worker examined].

**Distribution.** Wet, Dry and Intermediate Zones; *Primary literature records*: Ceylon (Forel 1908: 3), Haputale (Forel 1913a: 75), Bandarawela (Forel 1913b: 662), Polonnaruwa (Kosgamage and Dias 2009: 79), Anuradhapura, Polonnaruwa (Dias and Kosgamage 2012: 62), Sri Lanka (Dias 2014: 172), Namalweva, Ihakuluweva, Egodayagama (Dias and Peiris 2015a: 6), Sinhapura, Polonnaruwa, Mawathagama, Kurunegala (Dias and Peiris 2015b: 26), Anuradhapura, Colombo, Galle, Gampaha, Kurunegala, Polonnaruwa, Puttalam, Ratnapura (Dias and Rajapaksa 2016: 36); *Secondary literature records*: Sri Lanka (Dias 2002: 18), Sri Lanka (Hosoishi and Ogata 2009: 57), Sri Lanka (Dias et al. 2012: 15).

***Crematogaster rothneyi haputalensis* Forel, 1913**

Crematogaster rothneyi var. haputalensis Forel, 1913a: 75. TL: Haputale: [Ceylon] Sri Lanka [Syntype: MHNG?].

**Distribution.** Intermediate Zone; *Primary literature records*: Haputale (Forel 1913a: 75); *Secondary literature records*: Ceylon (Emery 1922: 152), Ceylon (Chapman and Capco 1951: 93), Sri Lanka (Dias 2006a: 51), Haputale (Hosoishi and Ogata 2009: 57).

***Crematogaster subnuda* Mayr, 1879**

*Crematogaster subnuda* Mayr, 1879: 682. TL: [Calcutta] Kolkata, West Bengal: India [Syntype: NHMW]. [Images of CASENT 0919689 syntype worker examined].

**Distribution.** Dry Zone; *Primary literature records*: Ceylon (Forel 1903a: 682), Trincomalee (Forel 1913b: 662); *Secondary literature records*: Ceylon (Emery 1922: 150), Sri Lanka (Ali 1992: 2), Sri Lanka (Tiwari 1999: 46), Sri Lanka (Tiwari et al. 1999: 251), Sri Lanka (Rajan et al. 2006: 176), Sri Lanka (Sheela 2008a: 24), Sri Lanka (Hosoishi and Ogata 2009: 58).

***Crematogaster walshi* Forel, 1902**

*Crematogaster walshi* Forel, 1902b: 205. TL: [Pooree] Puri: India [Syntype: MHNG]. [Images of CASENT 0908374 syntype worker examined].

**Distribution.** Wet Zone; *Primary literature records*: Peradeniya (Forel 1911b: 455).

#### *Dilobocondyla*: 1 species

***Dilobocondyla didita* (Walker, 1859)**

*Atta didita* Walker, 1859: 375. TL: [Ceylon] Sri Lanka [Syntypes: NHMUK]. [Images of CASENT 0902012 syntype worker examined].

**Distribution.** Wet Zone; *Primary literature records*: Ceylon (Walker 1859: 375), Peradeniya (Forel 1911a: 223); *Secondary literature records*: Peradenyia (Chapman and Capco 1951: 82), Sri Lanka (Dias 2002: 18), Sri Lanka (Dias 2006a: 51), Sri Lanka (Bharti and Kumar 2013: 42); *AntWeb records*: Peradenyia: CASENT 0902012, CASENT 0908983 (AntWeb 2020).

#### *Erromyrma*: 1 species

***Erromyrma latinodis* (Mayr, 1872)**

*Monomorium latinode* Mayr, 1872: 152. TL: Borneo, Sarawak: Malaysia [Lectotype: NHMUK]. [Images of CASENT 0905756, FOCOL 0634 lectotype and paralectotype workers examined].

**Distribution.** Wet Zone; *Primary literature records*: Kandy (Emery 1893a: 243), Sri Lanka (Bolton 1987: 430), South Sri Lanka (Way and Bolton 1997: 442), eastern Sri Lanka (Rickson and Rickson 1998: 843), Kandy (Heterick 2006: 108); *Secondary literature records*: Ceylon (Forel 1903a: 687), Ceylon (Emery 1922: 171), Ceylon (Chapman and Capco 1951: 165), Ceylon (Ettershank 1966: 88), Sri Lanka (Tiwari 1999: 55), Sri Lanka (Tiwari et al. 1999: 256), Sri Lanka (Dias 2002: 18), Sri Lanka (Tiwari and Tiwari 2002: 157), Sri Lanka (Tiwari et al. 2004: 611), Sri Lanka (Dias 2006a: 51), Sri Lanka (Rajan et al. 2006: 178), Sri Lanka (Sheela 2008a: 29), Sri Lanka (Tak 2008: 19), Sri Lanka (Dias et al. 2012: 15); *AntWeb records*: Kandy: CASENT 0008632 (AntWeb 2020).

#### *Lophomyrmex*: 3 species

***Lophomyrmex bedoti* Emery, 1893**

*Lophomyrmex bedoti* Emery, 1893c: 192. TL: Deli, Sumatra: Indonesia [Syntype: MSNG]. [Images of CASENT 0904647 syntype worker examined].

**Distribution.** Wet Zone; *Primary literature records*: Peradenyia (Rigato 1994: 55); *Secondary literature records*: Sri Lanka (Bharti and Kumar 2012b: 267), Sri Lanka (Yamane and Hosoishi 2014: 67).

***Lophomyrmex birmanus* Emery, 1893**

*Lophomyrmex birmanus* Emery, 1893c: 192. TL: Carin Cheba: Myanmar [Syntype: MSNG]. [Images of CASENT 0904646 syntype worker examined].

**Distribution.** Wet Zone; *Primary literature records*: Peradenyia (Rigato 1994: 58); *Secondary literature records*: Sri Lanka (Sheela and Ghosh 2008), Sri Lanka (Yamane and Hosoishi 2014: 67).

***Lophomyrmex quadrispinosus* (Jerdon, 1851)**

*Oecodoma quadrispinosa* Jerdon, 1851: 111. TL: Malabar, Kerala: India [Type: UNK].

**Distribution.** Wet, Dry and Intermediate Zones; *Primary literature records*: Kandy (Emery 1893a: 243), Ceylon (Emery 1893c: 192), Peradeniya (Forel 1911a: 223), Mahaoya, Sugarcane Research Institute, Katunayake, Lunuwila, C.R.I, Ratnapura, Kandy, Polonnaruwa, Laxapathiya nr Moratuwa, Mahawa (Rigato 1994: 59), Kelaniya, Gampaha, Colombo (Dias 2006a: 45), Nawalapitiya (Amarasinghe 2010: 12), Gilimale Forest Reserve (Dias and Perera 2011: 72), Anuradhapura, Polonnaruwa (Dias and Kosgamage 2012: 62), Sri Lanka (Dias 2014: 174), Namalweva, Ihakuluweva, Marawila, Pallama, Madurankuliya (Dias and Peiris 2015a: 6), Sinhapura, Polonnaruwa, Mawathagama, Kurunegala (Dias and Peiris 2015b: 26), Anuradhapura, Colombo, Galle, Gampaha, Kurunegala, Polonnaruwa, Puttalam, Ratnapura (Dias and Rajapaksa 2016: 36); *Secondary literature records*: Ceylon (Forel 1903a: 685), Ceylon (Wheeler 1936: 220), Ceylon (Chapman and Capco 1951: 156), Ceylon (Ettershank 1966: 134), Sri Lanka (Tiwari 1999: 59), Sri Lanka (Dias 2002: 17), Sri Lanka (Tiwari and Tiwari 2002: 158), Sri Lanka (Tiwari et al. 2003: 489), Sri Lanka (Dias 2006b: 8), Sri Lanka (Sheela and Ghosh 2008), Sri Lanka (Dias et al. 2012: 15), *AntWeb records*: Lunuwila CRI: CASENT 0281611, CASENT 0908880 (AntWeb 2020).

#### *Meranoplus*: 5 species

***Meranoplus bicolor* (Guerin-Meneville, 1844)**

*Cryptocerus bicolor* Guérin-Méneville, 1844: 425. TL: Pudicherry, Tamil Nadu: India [Type: UNK].

**Distribution.** Wet, Dry and Intermediate Zones; *Primary literature records*: Kandy, Galle, Kottawa, Colombo (Emery 1893a: 248), Ceylon (Emery 1901: 120), Ceylon (Forel 1903a: 705), Puwakpitiya, Galle (Forel 1908: 2), Peradeniya, Seenigoda (Forel 1911a: 226), Peradeniya, Seenigoda, Maha Iluppalama (Forel 1913a: 83), Ramboda (Karavaiev 1935: 99), Peradeniya, Henarathgoda, Colombo, Bandarawela, Dehiwala, Ratmalana, Laxapathiya, Polonnaruwa, Medirigiriya, Yakkala, Kandy, Moratuwa, Mahawa, Udugala, Sawaragomuwa, Opanayaka, Ratnapura, Kantale, Uva, Wellawaya (Schödl 1998: 372), Kelaniya, Gampaha, Colombo, Ratnapura, Galle (Dias 2006a: 45), Dambulla (Dias and Kosgamage 2008: 115), Sinharaja Forest Reserve (Gunawardene et al. 2008: 81), Nawalapitiya (Amarasinghe 2010: 12), Gilimale Forest Reserve (Dias and Perera 2011: 72), Anuradhapura, Polonnaruwa (Dias and Kosgamage 2012: 62), Sri Lanka (Dias 2014: 175), Ihakuluweva, Marawila, Pallama, Madurankuliya, Egodayagama (Dias and Peiris 2015a: 6), Mawathagama, Kurunegala (Dias and Peiris 2015b: 26), Anuradhapura, Colombo, Galle, Gampaha, Kurunegala, Polonnaruwa, Puttalam, Ratnapura (Dias and Rajapaksa 2016: 36), Indikada Mukalana Forest Reserve (Udayakantha and Dias 2018: 72); *Secondary literature records*: Sri Lanka (Dias 2002: 18), Sri Lanka (Tiwari and Tiwari 2002: 158), Sri Lanka (Tiwari et al. 2004: 612), Sri Lanka (Dias 2006b: 8), Sri Lanka (Tak 2008: 18), Sri Lanka (Tak and Kazmi 2011: 43), Sri Lanka (Dias et al. 2012: 15); *AntWeb records*: Dehiwala, Colombo: CASENT 0318981 (AntWeb 2020).

***Meranoplus boltoni* Schödl, 1998**

*Meranoplus boltoni* Schödl, 1998: 376. TL: Diyatalawa: Sri Lanka [Holotype: NHMUK]. [Images of CASENT 0902031 holotype worker examined].

**Distribution.** Intermediate Zone; *Primary literature records*: Diyatalawa (Schodl 1998: 376); *AntWeb records*: Diyatalawa: CASENT 0902031 (AntWeb 2020).

***Meranoplus levis* Donisthorpe, 1942**

*Meranoplus levis* Donisthorpe, 1942b: 455. TL: Dohnavur, Tinnevelly: India [Holotype: NHMUK]. [Images of CASENT 0902025 holotype worker examined].

**Distribution.** Dry Zone; *Primary literature records*: Hambantota (Donisthorpe 1942b: 456); Hambantota, Valleygatha (Schodl 1998: 381); *Secondary literature records*: Sri Lanka (Dias 2002: 18).

***Meranoplus loebli* Schödl, 1998**

*Meranoplus loebli* Schödl, 1998: 384. TL: Hasalaka: [Ceylon Central] Sri Lanka [Holotype: MHNG]. [Images of CASENT 0911194 holotype worker examined].

**Distribution.** Wet and Dry Zones; *Primary literature records*: Hasalaka, Kandy, Peradeniya, Kantale, Horton Plains (Schodl 1998: 384), Sinharaja Forest Reserve (Gunawardene et al. 2008: 81); *AntWeb records*: Hasalaka: CASENT 0902032, CASENT 0911194 (AntWeb 2020).

***Meranoplus rothneyi* Forel, 1902**

*Meranoplus rothneyi* Forel, 1902b: 241. TL: [Cochin] Kochi: India [Lectotype: NHMB]. [Images of CASENT 0915542 lectotype worker examined].

**Distribution.** Wet Zone; *Primary literature records*: Ceylon (Forel 1912d: 82), Sinharaja Forest Reserve (Gunawardene et al. 2008: 81).

#### *Metapone*: 1 species

***Metapone greeni* Forel, 1911**

*Metapone greeni* Forel, 1911b: 449. TL: Peradeniya: [Ceylon] Sri Lanka [Lectotype: MHNG]. [Images of CASENT 0907444 lectotype worker examined].

**Distribution.** Wet Zone; *Primary literature records*: Peradeniya (Forel 1911b: 452), Peradeniya (Wheeler 1919b: 181), Hantana (Karavaiev 1933: 120), Peradeniya, Hantana, Kandy (Taylor and Alpert 2016: 513); *Secondary literature records*: Peradeniya, Hantana (Smith, M. R. 1947: 75), Hantana (Chapman and Capco 1951: 114), Peradeniya, Hantana (Gregg 1958: 119, 120), Peradeniya, Hantana (Kusnezov 1960: 126), Sri Lanka (Dias 2002: 18), Sri Lanka (Dias 2006a: 51), Sri Lanka (Dias et al. 2012: 15); *AntWeb records*: Central Highlands, Peradeniya: CASENT 0907444, CASENT 0914068, CASENT 0916851 (AntWeb 2020).

#### *Monomorium*: 6 species

***Monomorium dichroum* Forel, 1902**

*Monomorium dichroum* Forel, 1902b: 212. TL: [Bombay] Mumbai, [Poona] Pune: India [Syntypes: MHNG, NHMUK, SIZK]. [Images of CASENT 0902222, CASENT 0908718, CASENT 0917812 syntype workers examined].

**Distribution.** Wet Zone; *Primary literature records*: Puwakpitiya (Forel 1908: 3), Seenigoda (Forel 1911a: 211), South Sri Lanka (Way and Bolton 1997: 442).

***Monomorium floricola* (Jerdon, 1851)**

*Atta floricola* Jerdon, 1851: 107. TL: Malabar, Thalassery, Kerala: India [Type: UNK].

**Distribution.** Wet, Dry and Intermediate Zones; *Primary literature records*: Dividsogala, Peradeniya (Forel 1911a: 221), Colombo (Bolton 1987: 391), South Sri Lanka (Way and Bolton 1997: 442), Kelaniya, Colombo (Dias 2006a: 45), Sinharaja Forest Reserve (Gunawardene et al. 2008: 81), Sinharaja Forest Reserve (Gunawardene et al. 2012: 84), Anuradhapura, Polonnaruwa (Dias and Kosgamage 2012: 62), Kirikanda Forest (Dias et al. 2013: 64), Sri Lanka (Dias 2014: 178), Namalweva, Ihakuluweva, Egodayagama (Dias and Peiris 2015a: 6), Sinhapura, Polonnaruwa, Mawathagama, Kurunegala (Dias and Peiris 2015b: 26), Anuradhapura, Colombo, Galle, Gampaha, Kurunegala, Polonnaruwa, Puttalam, Ratnapura (Dias and Rajapaksa 2016: 36); *Secondary literature records*: Ceylon (Emery 1922: 172), Ceylon (Chapman and Capco 1951: 164), Ceylon (Ettershank 1966: 89), Sri Lanka (Ali 1992: 3), Sri Lanka (Tiwari 1999: 54), Sri Lanka (Tiwari et al. 1999: 256), Sri Lanka (Dias 2002: 18), Sri Lanka (Tiwari and Tiwari 2002: 156), Sri Lanka (Tiwari et al. 2004: 610), Sri Lanka (Dias 2006b: 8), Peradeniya (Heterick 2006: 122), Sri Lanka (Li-Zhong 2006: 268), Sri Lanka (Rajan et al. 2006: 178), Sri Lanka (Dias et al. 2012: 15); *AntWeb records*: Peradenyia: CASENT 0908699, CASENT 0917813 (AntWeb 2020).

***Monomorium monomorium* Bolton, 1987**

*Monomorium monomorium* Bolton, 1987: 287. TL: Lido, Italy [Syntypes: NHMUK, NHMW]. [Images of CASENT 0902281, CASENT 0916000 syntype workers examined].

**Distribution.***Primary literature records*: Sri Lanka (Mathew and Tiwari 2000: 304); *Secondary literature records*: Sri Lanka (Dias 2002: 18), Sri Lanka (Li-Zhong 2006: 268).

***Monomorium pharaonis* (Linnaeus, 1758)**

*Formica pharaonis* Linnaeus, 1758: 580. TL: Egypt [Syntypes: UNK].

**Distribution.** Dry and Intermediate Zones; *Primary literature records*: Mihintale, Bibile (Bolton 1987: 356), Sri Lanka (Heterick 2006: 101), Anuradhapura, Polonnaruwa (Dias and Kosgamage 2012: 62), Sri Lanka (Dias 2014: 179), Namalweva, Ihakuluweva, Marawila, Madurankuliya (Dias and Peiris 2015a: 6), Sinhapura, Polonnaruwa (Dias and Peiris 2015b: 26), Anuradhapura, Kurunegala, Polonnaruwa, Puttalam (Dias and Rajapaksa 2016: 36); *Secondary literature records*: Sri Lanka (Dias 2002: 18), Sri Lanka (Dias 2006a: 51), Sri Lanka (Dias 2006b: 8), Sri Lanka (Dias et al. 2012: 15); *AntWeb records*: Sri Lanka: CASENT 0102682 (AntWeb 2020).

***Monomorium subopacum* (Smith F, 1858)**

*Myrmica subopaca* Smith F, 1858: 127. TL: Madeira Is.: Portugal [Lectotype: NHMUK]. [Images of CASENT 0010948 lectotype worker examined].

**Distribution.***Primary literature records*: Ceylon (Chapman and Capco 1951: 166), Sri Lanka (Bolton 1987: 360), Sri Lanka (Heterick 2006: 103); *Secondary literature records*: Ceylon (Emery 1922: 176), Ceylon (Ettershank 1966: 89), Sri Lanka (Tiwari 1999: 53), Sri Lanka (Dias 2002: 18), Sri Lanka (Dias 2006a: 51), Sri Lanka (Rajan et al. 2006: 179), Sri Lanka (Dias et al. 2012: 15); *AntWeb records*: Sri Lanka: CASENT 0010950, 0010951 (AntWeb 2020).

***Monomorium taprobanae* Forel, 1913**

Monomorium minutum var. taprobanae Forel, 1913a: 53. TL: Peradeniya: [Ceylon] Sri Lanka [Syntype: MHNG]. [Images of CASENT 0908703 syntype worker examined].

**Distribution.** Wet Zone; *Primary literature records*: Peradeniya (Forel 1913a: 53); *Secondary literature records*: Ceylon (Chapman and Capco 1951: 167), Ceylon (Ettershank 1966: 92), Sri Lanka (Dias 2006a: 51), Sri Lanka (Dias et al. 2012: 15); *AntWeb records*: Peradeniya: CASENT 0908703 (AntWeb 2020).

#### *Myrmecina*: 1 species

***Myrmecina striata* Emery, 1889**

*Myrmecina striata* Emery, 1889: 500. TL: Myanmar [Syntype: MSNG?].

**Distribution.** Wet and Dry Zones; *Primary literature records*: Sri Lanka (Dias et al. 2012: 15), Mihintale Forest (Dias and Kosgamage 2012: 62), Sri Lanka (Dias 2014: 180), Anuradhapura, Colombo, Galle, Gampaha, Polonnaruwa, Ratnapura (Dias and Rajapaksa 2016: 36).

#### *Myrmicaria*: 2 species

***Myrmicaria brunnea* Saunders, 1842**

*Myrmicaria brunnea* Saunders, 1842: 57. TL: India [Type: UNK].

**Distribution.** Wet and Dry Zones; *Primary literature records*: Ceylon (Forel 1903a: 708), Paradeniya (Forel 1907b: 17), Puwakpitiya, Kandy (Forel 1908: 2), Peradeniya (Forel 1911a: 226), Ceylon (Forel 1913a: 74), Ceylon (Karavaiev 1935: 82), Sinharaja Forest Reserve (Gunawardene et al. 2008: 81), Sinharaja Forest Reserve (Gunawardene et al. 2012: 84), Maimbula, Gampaha (Yahya et al. 2009: 251), Nawalapitiya (Amarasinghe 2010: 12), Gilimale Forest Reserve (Dias and Perera 2011: 72), Anuradhapura, Polonnaruwa (Dias and Kosgamage 2012: 62), Kirikanda Forest (Dias et al. 2013: 64), Sri Lanka (Dias 2014: 181), Kalugala Proposed Forest Reserve (Dias and Ruchirani 2014: 88), Anuradhapura, Colombo, Galle, Gampaha, Polonnaruwa, Ratnapura (Dias and Rajapaksa 2016: 36), Meethirigala Forest Reserve (Dias and Udayakantha 2016a: 53), Indikada Mukalana Forest Reserve (Dias and Udayakantha 2016b: 5), Indikada Mukalana Forest Reserve (Udayakantha and Dias 2018: 72); *Secondary literature records*: Ceylon (Emery 1922: 122), Ceylon (Donisthorpe 1942b: 454), Ceylon (Chapman and Capco 1951: 124), Sri Lanka (Ali 1992: 4), Sri Lanka (Tiwari 1999: 42), Sri Lanka (Tiwari et al. 1999: 257), Sri Lanka (Dias 2002: 18), Sri Lanka (Mathew and Tiwari 2000: 336), Sri Lanka (Tiwari and Tiwari 2002: 155), Sri Lanka (Dias 2006a: 51), Sri Lanka (Dias et al. 2012: 16).

***Myrmicaria fodiens* (Jerdon, 1851)**

*Myrmica fodicus* Jerdon, 1851: 115. TL: Malabar, Kerala: India [Type: UNK].

**Distribution.** Wet Zone; *Primary literature records*: Kandy (Emery 1893a: 249), Ceylon (Emery 1893c: 219), Ceylon (Emery 1901: 121).

#### *Paratopula*: 1 species

***Paratopula ceylonica* (Emery, 1901)**

*Atopomyrmex ceylonicus* Emery, 1901: 114. TL: Negombo: [Ceylon] Sri Lanka [Syntypes: DEIC, MSNG]. [Images of CASENT 0904714 syntype queen examined].

**Distribution.** Wet Zone; *Primary literature records*: Negombo (Emery 1901: 115), Colombo, Galle, Gampaha, Ratnapura (Dias and Rajapaksa 2016: 36); *Secondary literature records*: Negombo (Forel 1903a: 699), Ceylon (Chapman and Capco 1951: 118), Ceylon (Bolton 1988: 138), Sri Lanka (Dias 2002: 18), Sri Lanka (Dias 2006a: 51), Sri Lanka (Sheela 2008b: 423), Sri Lanka (Xu and Xu 2011: 595), Sri Lanka (Dias et al. 2012: 16); *AntWeb records*: Negombo: CASENT 0904714, FOCOL 0309 (AntWeb 2020).

#### *Pheidole*: 27 species/subspecies

***Pheidole barreleti* Forel, 1903**

*Pheidole barreleti* Forel, 1903b: 252. TL: Kandy: [Ceylon] Sri Lanka [Syntypes: MHNG]. [Images of CASENT 0907862, 0907863 syntype workers examined].

**Distribution.** Wet Zone; *Primary literature records*: Kandy (Forel 1903b: 252); *Secondary literature records*: Ceylon (Chapman and Capco 1951: 140), Sri Lanka (Dias 2002: 17), Sri Lanka (Dias 2006a: 51), Sri Lanka (Dias et al. 2012: 16); *AntWeb records*: Kandy: CASENT 0907862, 0907863 (AntWeb 2020).

***Pheidole ceylonica* (Motschoulsky, 1863)**

*Oecophthora ceylonica* Motschoulsky, 1863: 18. TL: [Ceylon] Sri Lanka [Type: UNK].

**Distribution.** Wet Zone; *Primary literature records*: Nuwara Eliya, Patannas, Colombo (Motschoulsky 1863: 18); *Secondary literature records*: Ceylon (Emery 1922: 97), Ceylon (Chapman and Capco 1951: 140), Sri Lanka (Dias 2002: 17), Sri Lanka (Dias 2006a: 51), Sri (Lanka Dias et al. 2012: 16).

***Pheidole diffidens* (Walker, 1859)**

*Formica diffidens* Walker, 1859: 372. TL: [Ceylon] Sri Lanka [Holotype: NHMUK]. [Images of CASENT 0901536 holotype worker examined].

**Distribution.***Primary literature records*: Ceylon (Walker 1859: 372); *Secondary literature records*: Emery 1925: 271 (Ceylon), Ceylon (Chapman and Capco 1951: 199), Sri Lanka (Dias 2002: 18), Sri Lanka (Dias 2006a: 51), Sri Lanka (Dias et al. 2012: 16); *AntWeb records*: Sri Lanka: CASENT 0901536.

***Pheidole fergusoni* Forel, 1902**

*Pheidole fergusoni* Forel, 1902b: 169. TL: Travancore: India [Syntypes: MHNG]. [Images of CASENT 0907877, 0907878 syntype workers examined].

**Distribution.** Dry Zone; *AntWeb records*: Ruhunu National Park: CASENT 0281642, 0281643 (AntWeb 2020).

***Pheidole fervens* Smith F, 1858**

*Pheidole fervens* Smith F, 1858: 176. TL: Singapore [Syntypes: NHMUK]. [Images of CASENT 0901519, 0901520 syntype workers examined].

**Distribution.** Wet Zone; *Primary literature records*: Sri Lanka (Ogata 1982: 197), Indikada Mukalana Forest Reserve (Udayakantha and Dias 2018: 73); *Secondary literature records*: Sri Lanka (Azuma and Kinjo 1987), Sri Lanka (Yamane et al. 1999), Sri Lanka (Li-Zhong 2006: 270), Sri Lanka (Terayama et al. 2014).

***Pheidole gracilipes* (Motschoulsky, 1863)**

*Leptomyrma gracilipes* Motschoulsky, 1863: 17. TL: Nuwara Eliya: [Ceylon] Sri Lanka [Type: UNK].

**Distribution.** Wet Zone; *Primary literature records*: Nuwara Eliya (Motschoulsky 1863: 17); *Secondary literature records*: Ceylon (Emery 1922: 97), Ceylon (Chapman and Capco 1951: 142), Sri Lanka (Dias 2002: 17), Sri Lanka (Dias 2006a: 51), Sri Lanka (Dias et al. 2012: 16).

***Pheidole horni* Emery, 1901**

*Pheidole horni* Emery, 1901: 118. TL: Bentota: [Ceylon] Sri Lanka [Syntype: MSNG]. [Images of CASENT 0904230 syntype worker examined].

**Distribution.** Wet Zone; *Primary literature records*: Bentota (Emery 1901: 118), Ceylon (Forel 1902b: 198), Ceylon (Forel 1902c: 546); *Secondary literature records*: Ceylon (Chapman and Capco 1951: 142), Sri Lanka (Dias 2002: 17), Sri Lanka (Tiwari et al. 2004: 612), Sri Lanka (Dias 2006a: 51), Sri Lanka (Dias et al. 2012: 16); *AntWeb records*: Bentota, Peradeniya: CASENT 0281653, CASENT 0904230 (AntWeb 2020).

***Pheidole indica* Mayr, 1879**

*Pheidole indica* Mayr, 1879: 679. TL: [Calcutta] Kolkata, West Bengal: India [Lectotype: NHMW]. [Images of CASENT 0906612, 0906613 paralectotype workers examined].

**Distribution.** Wet and Dry Zones; *Primary literature records*: Anuradhapura (Emery 1901: 118), Puwakpitiya (Forel 1908: 3), Trincomalee (Forel 1909: 394), Trincomalee, Peradeniya (Forel 1911a: 222); *Secondary literature records*: Ceylon (Forel 1902b: 199), Ceylon (Forel 1902c: 546), Ceylon (Chapman and Capco 1951: 143), Sri Lanka (Azuma and Kinjo 1987), Sri Lanka (Ogata 1982: 196), Sri Lanka (Yamane et al. 1999), Sri Lanka (Zhou and Zheng 1999: 84), Sri Lanka (Dias 2002: 17), Sri Lanka (Li-Zhong 2006: 270), Sri Lanka (Terayama et al. 2014); *AntWeb records*: Peradeniya: CASENT 0907901, 0907902 (AntWeb 2020).

***Pheidole jucunda* Forel, 1885**

*Pheidole jucunda* Forel, 1885: 179. TL: India [Syntype: MHNG?].

**Distribution.***Primary literature records*: Sri Lanka (Xu et al. 1998: 229); *Secondary literature records*: Sri Lanka (Tiwari et al. 1999: 260), Sri Lanka (Dias 2002: 17), Sri Lanka (Mathew and Tiwari 2000: 315).

***Pheidole latinoda* Roger, 1863**

*Pheidole latinoda* Roger, 1863: 195. TL: [Ceylon] Sri Lanka [Syntype: ZMHB?].

**Distribution.** Wet Zone; *Primary literature records*: Ceylon (Roger 1863: 196), Colombo (Emery 1893a: 243), Weligama (Emery 1901: 118); *Secondary literature records*: Ceylon (Forel 1902b: 189), Ceylon (Forel 1902c: 540), Ceylon (Chapman and Capco 1951: 144), Sri Lanka (Tiwari et al. 1999: 260), Sri Lanka (Dias 2002: 17), Sri Lanka (Dias 2006a: 51), Sri Lanka (Dias et al. 2012: 16).

***Pheidole latinoda peradeniyae* Forel, 1911**

*Pheidole latinoda peradeniyae* Forel, 1911a: 222. TL: Peradeniya: [Ceylon] Sri Lanka [Syntypes: MHNG]. [Images of CASENT 0907922, 0907923 syntype workers examined].

**Distribution.** Wet Zone; *Primary literature records*: Peradeniya (Forel 1911a: 222); *Secondary literature records*: Peradeniya (Chapman and Capco 1951: 144); *AntWeb records*: Peradeniya: CASENT 0907922, 0907923 (AntWeb 2020).

***Pheidole malinsii* Forel, 1902**

*Pheidole malinsii* Forel, 1902b: 167. TL: [Ceylon] Sri Lanka [Syntype: MHNG]. [Images of CASENT 0907927 syntype worker examined].

**Distribution.***Primary literature records*: Ceylon (Forel 1902b: 187), Ceylon (Forel 1902c: 539); *Secondary literature records*: Ceylon (Chapman and Capco 1951: 145), Sri Lanka (Tiwari 1999: 38), Sri Lanka (Dias 2002: 17), Sri Lanka (Mathew and Tiwari 2000: 316), Sri Lanka (Tiwari et al. 2003: 484), Sri Lanka (Dias 2006a: 51), Sri Lanka (Dias et al. 2012: 16); *AntWeb records*: Sri Lanka: CASENT 0907927 (AntWeb 2020).

***Pheidole megacephala* (Fabricius, 1793)**

*Formica megacephala* Fabricius, 1793: 361. TL: Camizard Mt., Bambous: Mauritius [Neotype: CAS]. [Images of CASENT 0104990 neotype worker examined].

**Distribution.** Wet, Dry and Intermediate Zones; *Primary literature records*: Ceylon (Smith F, 1858: 175); Colombo, Kandy (Emery 1893a: 243), Puwakpitiya (Forel 1908: 3), Peradeniya, Haputale (Forel 1913a: 27), Puttalam, Bandarawela (Forel 1913b: 662); *Secondary literature records*: Sri Lanka (Dias 2006a: 51), Sri Lanka (Dias et al. 2012: 16), Sri Lanka (Wetterer 2012b: 55), Ceylon (Fischer and Fischer 2013: 332).

***Pheidole nietneri* Emery, 1901**

*Pheidole nietneri* Emery, 1901: 118. TL: [Ceylon] Sri Lanka [Syntype: MSNG?].

**Distribution.** Wet and Dry Zones; *Primary literature records*: Bandarawela (Emery 1901: 119), Ceylon (Forel 1902b: 193), Ceylon (Forel 1902c: 543), Trincomalee (Forel 1909: 394); *Secondary literature records*: Ceylon (Chapman and Capco 1951: 146), Sri Lanka (Dias 2006a: 51), Sri Lanka (Dias et al. 2012: 16).

***Pheidole nodus* Smith F, 1874**

*Pheidole nodus* Smith F, 1874: 407. TL: Hyogo: Japan [Syntype: NHMUK]. [Images of CASENT 0901507 syntype worker examined].

**Distribution.** Wet and Dry Zones; *Primary literature records*: Ceylon (Forel 1902b: 195), Ceylon (Forel 1902c: 544), Peradeniya (Forel 1911a: 223), Puttalam (Forel 1913b: 662), Ceylon (Eguchi 2008: 59), Colombo, Galle, Gampaha, Ratnapura (Dias and Rajapaksa 2016: 36), Indikada Mukalana Forest Reserve (Dias and Udayakantha 2016b: 5), Indikada Mukalana Forest Reserve (Udayakantha and Dias 2018: 73); *Secondary literature records*: Ceylon (Wheeler 1929: 3), Ceylon (Chapman and Capco 1951: 148), Sri Lanka (Ogata 1982: 196), Sri Lanka (Dias 2002: 17), Sri Lanka (Dias 2006a: 51), Sri Lanka (Dias et al. 2012: 16), Sri Lanka (Terayama et al. 2014); *AntWeb records*: Sri Lanka: CASENT 0907942, 0907943, CASENT 0916618 (AntWeb 2020).

***Pheidole parva* Mayr, 1865**

*Pheidole parva* Mayr, 1865: 98. TL: [Ceylon] Sri Lanka [Lectotype: NHMW]. [Images of CASENT 0319248 lectotype worker examined].

**Distribution.** Wet and Intermediate Zones; *Primary literature records*: Ceylon (Mayr 1865: 98), Ceylon (Forel 1902b: 192), Ceylon (Forel 1902c: 542), Peradeniya (Forel 1911a: 221), Haputale (Forel 1913a: 41), Kandy (Eguchi et al. 2007: 264), Kandy (Eguchi 2008: 67), Sri Lanka (Fischer and Fischer 2013: 340); *Secondary literature records*: Ceylon (Emery 1922: 95), Ceylon (Chapman and Capco 1951: 147), Sri Lanka (Mathew and Tiwari 2000: 317), Sri Lanka (Dias 2002: 17), Sri Lanka (Jaitrong and Nabhitabhata 2005: 34), Sri Lanka (Dias 2006a: 51), Sri Lanka (Ghosh et al. 2006: 390), Sri Lanka (Le et al. 2010: 7), Sri Lanka (Dias et al. 2012: 16); *AntWeb records*: Sri Lanka: CASENT 0922207, 0922208, CASENT 0319248 (AntWeb 2020).

***Pheidole pronotalis* Forel, 1902**

*Pheidole pronotalis* Forel, 1902b: 173. TL: [Ceylon] Sri Lanka [Syntypes: MHNG]. [Images of CASENT 0907987, 0907988 syntype workers examined].

**Distribution.** Wet Zone; *Primary literature records*: Kandy, Nuwara Eliya (Emery 1893a: 244), Ceylon (Forel 1902b: 190), Ceylon (Forel 1902c: 541); *Secondary literature records*: Ceylon (Emery 1922: 95), Ceylon (Chapman and Capco 1951: 148), Sri Lanka (Mathew and Tiwari 2000: 317), Sri Lanka (Dias 2002: 17), Sri Lanka (Tiwari et al. 2003: 484), Sri Lanka (Dias 2006a: 52), Sri Lanka (Dias et al. 2012: 16); *AntWeb records*: Sri Lanka: CASENT 0907987, 0907988 (AntWeb 2020).

***Pheidole rogersi taylori* Forel, 1902**

*Pheidole rogersi taylori* Forel, 1902b: 182. TL: [Orissa] Odisha: India [Syntypes: MHNG]. [Images of CASENT 0907949, 0907950 syntype workers examined].

**Distribution.** Wet Zone; *Primary literature records*: Peradeniya (Forel 1911a: 222).

***Pheidole rugosa* Smith F, 1858**

*Pheidole rugosa* Smith F, 1858: 175. TL: [Ceylon] Sri Lanka [Holotype: NHMUK]. [Images of CASENT 0901525 holotype worker examined].

**Distribution.***Primary literature records*: Ceylon (Smith F, 1858: 176), Ceylon (Forel 1902b: 194), Ceylon (Forel 1902c: 544); *Secondary literature records*: Ceylon (Chapman and Capco 1951: 149), Sri Lanka (Dias 2002: 17), Sri Lanka (Dias 2006a: 52), Sri Lanka (Dias et al. 2012: 16); *AntWeb records*: Sri Lanka: CASENT 0901525 (AntWeb 2020).

***Pheidole sharpi* Forel, 1902**

*Pheidole sharpi* Forel, 1902b: 169. TL: Salem, Madras Pres.: India [Syntypes: MHNG]. [Images of CASENT 0907951, 0907952 syntype workers examined].

**Distribution.** Dry Zone; *Primary literature records*: Sri Lanka (Dias 2002: 17); *AntWeb records*: Wirawila: CASENT 0281640, 0281641 (AntWeb 2020).

***Pheidole spathifera* Forel, 1902**

*Pheidole spathifera* Forel, 1902b: 168. TL: Coonoor, [Nilghiris] Tamil Nadu: India [Syntypes: MHNG, SIZK]. [Images of CASENT 0907955, 0907956, CASENT 0917784, 0917785 syntype workers examined].

**Distribution.** Wet Zone; *Primary literature records*: Ceylon (Forel 1902b: 187), Ceylon (Forel 1902c: 539), Kelaniya (Dias 2006a: 45), Colombo, Galle, Gampaha, Ratnapura (Dias and Rajapaksa 2016: 36); *Secondary literature records*: Sri Lanka (Ali 1992: 4), Sri Lanka (Tiwari 1997: 444), Sri Lanka (Xu et al. 1998: 234), Sri Lanka (Tiwari 1999: 39), Sri Lanka (Tiwari et al. 1999: 261), Sri Lanka (Dias 2002: 17), Sri Lanka (Tiwari and Tiwari 2002: 155), Sri Lanka (Rajan et al. 2006: 180), Sri Lanka (Dias et al. 2012: 16); *AntWeb records*: Nuwara Eliya (AntWeb 2020: CASENT 0281634, 0281635).

***Pheidole spathifera yerburyi* Forel, 1902**

*Pheidole spathifera yerburyi* Forel, 1902b: 168. TL: [Ceylon] Sri Lanka [Syntypes: MHNG]. [Images of CASENT 0907959, 0907960 syntype workers examined].

**Distribution.** Wet Zone; *Primary literature records*: Bandarawela (Emery 1901: 118), Ceylon (Forel 1902b: 188), Ceylon (Forel 1902c: 540), Puwakpitiya (Forel 1908: 3), Peradeniya, Seenigoda (Forel 1911a: 221); *Secondary literature records*: Ceylon (Chapman and Capco 1951: 150); *AntWeb records*: Sri Lanka: CASENT 0907959, 0907960 (AntWeb 2020).

***Pheidole sulcaticeps* Roger, 1863**

*Pheidole sulcaticeps* Roger, 1863: 193. TL: [Ceylon] Sri Lanka [Syntypes: ZMHB].

**Distribution.** Dry Zone; *Primary literature records*: Ceylon (Roger 1863: 195), Maha Iluppalama (Forel 1913a: 42); *Secondary literature records*: Ceylon (Chapman and Capco 1951: 151), Sri Lanka (Tiwari et al. 1999: 261), Sri Lanka (Dias 2002: 17), Sri Lanka (Tiwari and Tiwari 2002: 155), Sri Lanka (Tak and Rathore 2004a: 181), Sri Lanka (Dias 2006a: 52), Sri Lanka (Li-Zhong 2006: 270), Sri Lanka (Tak 2008: 28), Sri Lanka (Dias et al. 2012: 16).

***Pheidole sulcaticeps vellicans* Forel, 1911**

*Pheidole sulcaticeps vellicans* Forel, 1911a: 222. TL: Peradeniya: [Ceylon] Sri Lanka [Syntypes: MHNG]. [Images of CASENT 0907963, 0907964 syntype workers examined].

**Distribution.** Wet Zone; *Primary literature records*: Peradeniya (Forel 1911a: 222); *Secondary literature records*: Ceylon (Chapman and Capco 1951: 151); *AntWeb records*: Peradeniya: CASENT 0907963, 0907964 (AntWeb 2020).

***Pheidole templaria euscrobata* Forel, 1913**

Pheidole templaria r. euscrobata Forel, 1913a: 41. TL: Haputale: [Ceylon] Sri Lanka [Syntypes: MHNG, ZMHB]. [Images of CASENT 0908004, FOCOL 1376 syntype workers examined].

**Distribution.** Intermediate Zone; *Primary literature records*: Haputale (Forel 1913a: 41); *Secondary literature records*: Ceylon (Emery 1922: 95), Ceylon (Chapman and Capco 1951: 151); *AntWeb records*: Haputale: CASENT 0908004, FOCOL 1376 (AntWeb 2020).

***Pheidole watsoni* Forel, 1902**

*Pheidole watsoni* Forel, 1902b: 171. TL: Myingyan, [N. Burma] Myanmar; [Orissa] Odisha: India [Syntypes: MHNG]. [Images of CASENT 0908005, 0908006 syntype workers examined].

**Distribution.***Primary literature records*: Ceylon (Forel 1902b: 189); *Secondary literature records*: Ceylon (Emery 1922: 95), Ceylon (Chapman and Capco 1951: 152), Sri Lanka (Xu et al. 1998: 232), Sri Lanka (Tiwari et al. 1999: 262), Sri Lanka (Mathew and Tiwari 2000: 319), Sri Lanka (Dias 2002: 17), Sri Lanka (Ghosh et al. 2006: 390), Sri Lanka (Rajan et al. 2006: 181).

***Pheidole woodmasoni* Forel, 1885**

*Pheidole woodmasoni* Forel, 1885: 180. TL: [Calcutta] Kolkata, West Bengal; [Orissa] Odisha: India [Syntypes: MHNG]. [Images of CASENT 0908007, 0908008 syntype workers examined].

**Distribution.** Wet and Intermediate Zones; *Primary literature records*: Matale, Kandy (Emery 1893a: 243), Ceylon (Forel 1902b: 191), Ceylon (Forel 1902c: 541); *Secondary literature records*: Ceylon (Emery 1922: 95), Ceylon (Chapman and Capco 1951: 152), Sri Lanka (Ali 1992: 5), Sri Lanka (Tiwari et al. 1999: 262), Sri Lanka (Mathew and Tiwari 2000: 320), Sri Lanka (Dias 2002: 17), Sri Lanka (Rajan et al. 2006: 181), Sri Lanka (Sheela 2008a: 37).

#### *Pristomyrmex*: 2 species

***Pristomyrmex punctatus* (Smith F, 1860)**

*Myrmica punctata* Smith F, 1860a: 108. TL: [Bac.] Bacan, Maluku Utara: Indonesia [Syntype: OUMNH]. [Images of CASENT 0901379 syntype worker examined].

**Distribution.***Primary literature records*: Ceylon (Forel 1903a: 696); *Secondary literature records*: Sri Lanka (Dias 2002: 18).

***Pristomyrmex sinharaja* Dias & Yamane, 2016**

*Pristomyrmex sinharaja* Dias & Yamane, 2016: 190. TL: Sinharaja NP, Sri Lanka [Holotype: SKYC]. [Images of ANTWEB 1041160 holotype worker examined].

**Distribution.** Wet Zone; *Primary literature records*: Sinharaja National Park (Yamane and Dias 2016: 190), Colombo, Galle, Gampaha, Ratnapura (Dias and Rajapaksa 2016: 36); *AntWeb records*: Sinharaja National Park: ANTWEB 1041160, 1041161 (AntWeb 2020).

#### *Recurvidris*: 2 species

***Recurvidris pickburni* Bolton, 1992**

*Recurvidris pickburni* Bolton, 1992: 45. TL: Kandy: Sri Lanka [Paratype: NHMUK]. [Images of CASENT 0902055 paratype worker examined].

**Distribution.** Wet Zone; *Primary literature records*: Kandy (Bolton 1992: 45), Sinharaja Forest Reserve (Gunawardene et al. 2012: 85), Sri Lanka (Dias 2014: 186); *Secondary literature records*: Sri Lanka (Dias 2006a: 52), Sri Lanka (Dias et al. 2012: 16); *AntWeb records*: Kandy: CASENT 0902055 (AntWeb 2020).

***Recurvidris recurvispinosa* (Forel, 1890)**

*Trigonogaster recurvispinosus* Forel, 1890: cix. TL: [Poona] Pune: India [Syntype: MHNG]. [Images of CASENT 0908882 syntype worker examined].

**Distribution.** Wet, Dry and Intermediate Zones; *Primary literature records*: Sri Lanka (Dias et al. 2012: 16), Giritale Forest, Mihintale (Dias and Kosgamage 2012: 63), Sri Lanka (Dias 2014: 187), Mawathagama, Kurunegala (Dias and Peiris 2015b: 26), Anuradhapura, Colombo, Galle, Gampaha, Kurunegala, Polonnaruwa, Ratnapura (Dias and Rajapaksa 2016: 36), Indikada Mukalana Forest Reserve (Udayakantha and Dias 2018: 73).

#### *Rhopalomastix*: 2 species

***Rhopalomastix escherichi* Forel, 1911**

*Rhopalomastix escherichi* Forel, 1911a: 217. TL: Peradenyia: [Ceylon] Sri Lanka [Syntype: MHNG]. [Images of CASENT 0908321 syntype queen examined].

**Distribution.** Wet Zone; *Primary literature records*: Peradenyia (Forel 1911a: 217); *Secondary literature records*: Ceylon (Emery 1922: 119), Ceylon (Chapman and Capco 1951: 111), Sri Lanka (Dias 2002: 18), Sri Lanka (Dias 2006a: 52), Sri Lanka (Dias et al. 2012: 16); *AntWeb records*: Peradenyia: CASENT 0908321 (AntWeb 2020).

***Rhopalomastix rothneyi* Forel, 1900**

*Rhopalomastix rothneyi* Forel, 1900c: 24. TL: Barrackpore, West Bengal: India; [Ceylon] Sri Lanka [Syntypes: MHNG, ZMHB]. [Images of CASENT 0908322, 0908323, FOCOL 2102 syntype workers examined].

**Distribution.** Wet Zone; *Primary literature records*: Peradenyia (Forel 1911a: 217), Sinharaja Forest Reserve (Gunawardene et al. 2012: 85); *Secondary literature records*: Ceylon (Emery 1922: 119), Ceylon (Chapman and Capco 1951: 111), Sri Lanka (Dias 2002: 18), Peradenyia: *AntWeb records*: CASENT 0908323, FOCOL 2102 (AntWeb 2020).

#### *Solenopsis*: 2 species

***Solenopsis geminata* (Fabricius, 1804)**

*Atta geminata* Fabricius, 1804: 423. TL: Central America [Syntypes: ZMUC].

**Distribution.** Wet, Dry and Intermediate Zones; *Primary literature records*: Ceylon (Mayr 1865: 109), Colombo (Emery 1893a: 243), Ceylon (Emery 1901: 120), Galle (Forel 1908: 3), Ambalangoda (Forel 1909: 394), Seenigoda (Forel 1913a: 55), South Sri Lanka (Way and Bolton 1997: 442), Kelaniya, Gampaha, Colombo, Ratnapura, Galle (Dias 2006a: 45), Dambulla (Dias and Kosgamage 2008: 115), Nawalapitiya (Amarasinghe 2010: 12), Anuradhapura, Polonnaruwa (Dias and Kosgamage 2012: 63), Sri Lanka (Dias 2014: 189), Namalweva, Marawila, Pallama, Madurankuliya (Dias and Peiris 2015a: 6), Sinhapura, Polonnaruwa (Dias and Peiris 2015b: 26), Anuradhapura, Colombo, Galle, Gampaha, Kurunegala, Polonnaruwa, Puttalam, Ratnapura (Dias and Rajapaksa 2016: 36); *Secondary literature records*: Ceylon (Chapman and Capco 1951: 168), Sri Lanka (Dias 2002: 18), Sri Lanka (Tak and Rathore 2004a: 176), Sri Lanka (Dias 2006b: 8), Sri Lanka (Tak 2008: 26), Sri Lanka (Wetterer 2011: 27), Sri Lanka (Dias et al. 2012: 16).

***Solenopsis nitens* Bingham, 1903**

*Solenopsis nitens* Bingham, 1903: 160. TL: Peradeniyan near Kandy: [Ceylon] Sri Lanka [Holotype: NHMUK]. [Images of CASENT 0902364 holotype worker examined].

**Distribution.** Wet Zone; *Primary literature records*: Peradeniya (Bingham 1903: 160), Peradeniya (Forel 1911b: 455); *Secondary literature records*: Ceylon (Chapman and Capco 1951: 168), Ceylon (Ettershank 1966: 142), Sri Lanka (Dias 2002: 18), Sri Lanka (Dias 2006a: 52), Sri Lanka (Dias et al. 2012: 16); *AntWeb records*: Peradeniya: CASENT 0902364 (AntWeb 2020).

#### *Stereomyrmex*: 1 species

***Stereomyrmex horni* Emery, 1901**

*Stereomyrmex horni* Emery, 1901: 116. TL: Bandarawela, Uva: [Ceylon] Sri Lanka [Syntypes: DEIC, MSNG]. [Images of CASENT 0904458, 0904459, FOCOL 0290–0295 syntype workers and males examined].

**Distribution.** Wet, Dry and Intermediate Zones; *Primary literature records*: Bandarawela (Emery 1901: 117), Bandarawela (Forel 1903a: 699), Bandarawela (Forel 1908: 3), Anuradhapura Sanctuary (Dias et al. 2011: 100), Sri Lanka (Dias 2014: 190), Kalugala Proposed Forest Reserve, Wilpita “Aranya Kele” (Dias and Ruchirani 2014: 88), Anuradhapura, Colombo, Galle, Gampaha, Polonnaruwa, Ratnapura (Dias and Rajapaksa 2016: 36); *Secondary literature records*: Ceylon (Emery 1922: 120), Ceylon (Chapman and Capco 1951: 173), Sri Lanka (Dias 2002: 18), Sri Lanka (Dias 2006a: 52), Sri Lanka (Dias et al. 2012: 16); *AntWeb records*: Diyatalawa, Bandarawela: CASENT 0101263, 0101264, CASENT 0101796, CASENT 0178593, CASENT 0904458, 0904459, CASENT 0922896, FOCOL 0290–0295, MCZ 594252 (AntWeb 2020).

#### *Strumigenys*: 6 species

***Strumigenys emmae* (Emery, 1890)**

*Epitritus emmae* Emery, 1890: 70. TL: [S. Thomas], Virgin Islands: United States [Syntype: MSNG]. [Images of CASENT 0102082 syntype worker examined].

**Distribution.** Wet Zone; *Primary literature records*: Indikada Mukalana Forest Reserve, Lenagala Forest Reserve, Watinapaha (Dias et al. 2018: 252), Indikada Mukalana Forest Reserve (Udayakantha and Dias 2018: 73).

***Strumigenys godeffroyi* Mayr, 1866**

*Strumigenys godeffroyi* Mayr, 1866: 516. TL: Samoa [Syntype: NHMW]. [Images of CASENT 0915692 syntype worker examined].

**Distribution.** Wet Zone; *Primary literature records*: Kandy (Emery 1893a: 249), Peradeniya (Forel 1913a: 84), Peradeniya, Gilimale (Bolton 2000: 791); *Secondary literature records*: Ceylon (Emery 1914: 417), Ceylon (Chapman and Capco 1951: 108), Sri Lanka (Lin and Wu 1996: 143), Sri Lanka (Dias 2002: 18), Sri Lanka (Dias 2006a: 52), Sri Lanka (Li-Zhong 2006: 272), Sri Lanka (Dias et al. 2012: 16); *AntWeb records*: Peradeniya: CASENT 0909311 (AntWeb 2020).

***Strumigenys inopinata* (De Andrade, 1994)**

*Rhopalothrix inopinata* De Andrade, 1994: 54. TL: Kandy: [Ceylon] Sri Lanka [Holotype: MHNG]. [Images of CASENT 0911230 holotype worker examined].

**Distribution.** Wet Zone; *Primary literature records*: Kandy (Baroni Urbani and De Andrade 1994: 54); *AntWeb records*: Kandy: CASENT 0911230 (AntWeb 2020).

***Strumigenys lyroessa* (Roger, 1862)**

*Labidogenys lyroessa* Roger, 1862: 251. TL: [Ceylon] Sri Lanka [Syntype: ZMHB]. [Images of FOCOL 2155 syntype worker examined].

**Distribution.** Wet and Dry Zones; *Primary literature records*: Ceylon (Roger 1862: 251), Ceylon (Forel 1903a: 707), Anuradhapura, Polonnaruwa (Dias and Kosgamage 2012: 63), Sri Lanka (Dias 2014: 192), Anuradhapura, Colombo, Galle, Gampaha, Polonnaruwa, Ratnapura (Dias and Rajapaksa 2016: 36); *Secondary literature records*: Ceylon (Emery 1897a: 574), Ceylon (Chapman and Capco 1951: 109), Sri Lanka (Bolton 2000: 872), Sri Lanka (Dias 2002: 18), Sri Lanka (Dias 2006a: 52), Sri Lanka (Dias et al. 2012: 16); *AntWeb records*: Sri Lanka: FOCOL 2155 (AntWeb 2020).

***Strumigenys membranifera* Emery, 1869**

*Strumigenys membranifera* Emery, 1869: 24. TL: Portici: Italy [Syntype: MSNG]. [Images of CASENT 0102081 syntype worker examined].

**Distribution.** Wet Zone; *Primary literature records*: Watinapaha (Dias et al. 2018: 253).

***Strumigenys veddha* De Andrade, 2007**

*Strumigenys veddha* De Andrade, 2007: 141. TL: North Central, Aluthoya: Sri Lanka [Holotype: MHNG]. [Images of CASENT 0911244 holotype worker examined].

**Distribution.** Dry Zone; *Primary literature records*: North central, Aluthoya (Baroni Urbani and De Andrade 2007: 141); *AntWeb records*: North Central, Aluthoya: CASENT 0911244 (AntWeb 2020).

#### *Syllophopsis*: 1 species

***Syllophopsis australica* (Forel, 1907)**

Monomorium subcoecum subsp. australicum Forel, 1907a: 20. TL: Mount Victoria, New South Wales: Australia [Lectotype: HNHM]. [Images of CASENT 0916610 lectotype worker examined].

**Distribution.***Primary literature records*: South Sri Lanka (Way and Bolton 1997: 442).

#### *Tetramorium*: 16 species

***Tetramorium bicarinatum* (Nylander, 1846)**

*Myrmica bicarinata* Nylander, 1846b: 1061. TL: California: USA [Syntypes: Lost].

**Distribution.** Wet, Dry and Intermediate Zones; *Primary literature records*: Mahaoya Distr., Udugala, Laxapathiya, Panadura, Galle Distr., Udugama (Bolton 1977: 96), Gilimale Forest Reserve (Dias and Perera 2011: 72), Anuradhapura, Polonnaruwa (Dias and Kosgamage 2012: 63), Sri Lanka (Dias 2014: 193), Kalugala Proposed Forest Reserve (Dias and Ruchirani 2014: 88), Namalweva, Ihakuluweva, Pallama, Madurankuliya, Egodayagama (Dias and Peiris 2015a: 6), Sinhapura, Polonnaruwa (Dias and Peiris 2015b: 26), Anuradhapura, Colombo, Galle, Gampaha, Kurunegala, Polonnaruwa, Puttalam, Ratnapura (Dias and Rajapaksa 2016: 36), Indikada Mukalana Forest Reserve (Dias and Udayakantha 2016b: 5), Indikada Mukalana Forest Reserve (Udayakantha and Dias 2018: 73); *Secondary literature records*: Sri Lanka (Mathew and Tiwari 2000: 268), Sri Lanka (Mathew 2003: 201), Sri Lanka (Dias 2006a: 52), Sri Lanka (Dias et al. 2012: 16).

***Tetramorium curvispinosum* Mayr, 1897**

*Tetramorium curvispinosum* Mayr, 1897: 430. TL: Kalawewa: [Ceylon] Sri Lanka [Syntype: HNHM]. [Images of CASENT 0916589 syntype worker examined].

**Distribution.** Wet and Dry Zones; *Primary literature records*: Kalawewa (Mayr 1897: 431), Kalawewa (Forel 1903a: 702), Kandy (Bolton 1977: 122); *Secondary literature records*: Ceylon (Chapman and Capco 1951: 177), Sri Lanka (Dias 2002: 18), Sri Lanka (Dias 2006a: 52), Sri Lanka (Dias et al. 2012: 16); *AntWeb records*: Kandy, Kalawewa: CASENT 0280895, CASENT 0916589 (AntWeb 2020).

***Tetramorium indicum* Forel, 1913**

Tetramorium guineense var. indica Forel, 1913a: 81. TL: [Poona] Pune: India [Syntype: MHNG]. [Images of CASENT 0909109 syntype worker examined].

**Distribution.** Wet Zone; *Primary literature records*: Ratnapura, Colombo, Yakkala (Bolton 1977: 99); *Secondary literature records*: Sri Lanka (Dias 2002: 18).

***Tetramorium insolens* (Smith F, 1861)**

*Myrmica insolens* Smith F, 1861: 47. TL: Sulawesi, Manado, Sulawesi Utara: Indonesia [Holotype: OUMNH]. [Images of CASENT 0235208 holotype queen examined].

**Distribution.** Wet Zone; *Primary literature records*: Ratnapura (Bolton 1977: 100); *Secondary literature records*: Sri Lanka (Dias 2002: 18) (Hita Garcia and Fisher 2011: 22).

***Tetramorium lanuginosum* Mayr, 1870**

*Tetramorium lanuginosum* Mayr, 1870: 976. TL: Java, Batavia: Indonesia [Holotype: NHMW]. [Images of FOCOL 2063 holotype queen examined].

**Distribution.** Wet and Dry Zones; *Primary literature records*: Kandy (Emery 1893a: 248), Kandy (Forel 1903a: 704), Ceylon (Forel 1908: 2), Polonnaruwa (Bolton 1976: 351), South Sri Lanka (Way and Bolton 1997: 442); *Secondary literature records*: Ceylon (Forel 1901b: 10), Ceylon (Viehmeyer 1912: 21), Ceylon (Menozzi 1934: 162), Ceylon (Chapman and Capco 1951: 179), Sri Lanka (Mathew and Tiwari 2000: 298), Sri Lanka (Tiwari et al. 2003: 491), Sri Lanka (Ghosh et al. 2006: 389), Sri Lanka (Li-Zhong 2006: 273), Sri Lanka (Wetterer 2010: 84).

***Tetramorium mayri* (Forel, 1912)**

*Rhoptromyrmex mayri* Forel, 1912c: 57. TL: [Poona] Pune: India [Syntypes: MHNGNHMUK]. [Images of CASENT 0901034, CASENT 0909199 syntype queens examined].

**Distribution.** Wet Zone; *Primary literature records*: Colombo (Forel 1912c: 58).

***Tetramorium obesum* André, 1887**

*Tetramorium obesum* André, 1887: 294. TL: Gingi: India [Syntypes: MCZ, MNHN].

**Distribution.** Dry Zone; *AntWeb records*: Lunugamvehera, near Wirawila: CASENT 0280874 (AntWeb 2020).

***Tetramorium pacificum* Mayr, 1870**

*Tetramorium pacificum* Mayr, 1870: 976. TL: Tonga [Syntype: MSNG]. [Images of CASENT 0904848 syntype worker examined].

**Distribution.** Wet, Dry and Intermediate Zones; *Primary literature records*: Kandy, Colombo (Emery 1893a: 246), Kandy, Colombo (Forel 1903a: 702), Ceylon (Forel 1909: 394), Peradeniya, Seenigoda (Forel 1911a: 223), Colombo, Laxapathiya, Yakkala, Dehiwala, Peradeniya, Gilimale, Ratnapura, Kandy, Koslanda, Bibile (Bolton 1977: 102), South Sri Lanka (Way and Bolton 1997: 442), Kandy, Colombo (Hita Garcia and Fisher 2011: 24), Indikada Mukalana Forest Reserve (Udayakantha and Dias 2018: 73); *Secondary literature records*: Ceylon (Cheesman and Crawley 1928: 522), Ceylon (Chapman and Capco 1951: 176), Kandy (Taylor 1987: 79), Sri Lanka (Dias 2002: 18), Sri Lanka (Tiwari et al. 2003: 490), Sri Lanka (Dias 2006a: 52), Sri Lanka (Dias et al. 2012: 16); *AntWeb records*: Kandy: CASENT 0904850 (AntWeb 2020).

***Tetramorium pilosum* Emery, 1893**

*Tetramorium pilosum* Emery, 1893a: 247 TL: Kandy: [Ceylon] Sri Lanka [Holotype: MSNG]. [Images of CASENT 0281188 holotype worker examined].

**Distribution.** Wet Zone; *Primary literature records*: Kandy (Emery 1893a: 247); Ceylon (Emery 1901: 120), Kandy (Forel 1903a: 702), Kandy (Bolton 1977: 82); Indikada Mukalana Forest Reserve (Udayakantha and Dias 2018: 73); *Secondary literature records*: Ceylon (Chapman and Capco 1951: 180), Sri Lanka (Dias 2002: 18), Sri Lanka (Dias 2006a: 52), Sri Lanka (Dias et al. 2012: 16); *AntWeb records*: Kandy: CASENT 0280881, CASENT 0281188 (AntWeb 2020).

***Tetramorium simillimum* (Smith F, 1851)**

*Myrmica simillima* Smith F, 1851: 118. TL: Great Britian: England [Syntypes: Lost].

**Distribution.** Wet Zone; *Primary literature records*: Peradeniya (Forel 1913a: 81), Yakkala, Peradeniya (Bolton 1977: 132), Peradeniya (Bolton 1980: 320), South Sri Lanka (Way and Bolton 1997: 442); *Secondary literature records*: Ceylon (Chapman and Capco 1951: 177), Sri Lanka (Mathew and Tiwari 2000: 310), Sri Lanka (Dias 2002: 18), Sri Lanka (Dias 2006a: 52), Sri Lanka (Dias et al. 2012: 16); *AntWeb records*: Peradenyia: FOCOL 2100 (AntWeb 2020).

***Tetramorium smithi* Mayr, 1879**

*Tetramorium smithi* Mayr, 1879: 673. TL: [Calcutta] Kolkata, West Bengal: India [Syntypes: NHMUK, NHMW]. [Images of CASENT 0901108, CASENT 0919654 syntype workers examined].

**Distribution.** Wet, Dry and Intermediate Zones; *Primary literature records*: Boragas, Nuwara Eliya (Bolton 1977: 90), Anuradhapura, Polonnaruwa (Dias and Kosgamage 2012: 63), Sri Lanka (Dias 2014: 194), Ihakuluweva, Pallama, Madurankuliya, Egodayagama (Dias and Peiris 2015a: 6), Anuradhapura, Colombo, Galle, Gampaha, Kurunegala, Polonnaruwa, Puttalam, Ratnapura (Dias and Rajapaksa 2016: 36), Indikada Mukalana Forest Reserve (Dias and Udayakantha 2016b: 5), Indikada Mukalana Forest Reserve (Udayakantha and Dias 2018: 73); *Secondary literature records*: Sri Lanka (Yamane et al. 1999), Sri Lanka (Li-Zhong 2006: 273), Sri Lanka (Le et al. 2010: 9), Sri Lanka (Dias et al. 2012: 16), Sri Lanka (Terayama et al. 2014).

***Tetramorium tonganum* Mayr, 1870**

*Tetramorium tonganum* Mayr, 1870: 976. TL: [Tongatabu] Tongatapu: Tonga [Syntype: NHMW]. [Images of CASENT 0916043 syntype worker examined].

**Distribution.** Wet Zone; *Primary literature records*: Peradeniya (Forel 1911a: 225); Peradeniya (Bolton 1977: 129); *Secondary literature records*: Ceylon (Chapman and Capco 1951: 176), Sri Lanka (Dias 2002: 18); *AntWeb records*: Peradeniya: CASENT 0909171 (AntWeb 2020).

***Tetramorium tortuosum* Roger, 1863**

*Tetramorium tortuosum* Roger, 1863: 181. TL: [Ceylon] Sri Lanka [Syntypes: ZMHB?].

**Distribution.** Wet, Dry and Intermediate Zones; *Primary literature records*: Ceylon (Roger 1863: 181), Kandy (Emery 1893a: 246), Kandy (Forel 1903a: 702), Ceylon (Forel 1911a: 225), Nuwara Eliya, Hakgala (Bolton 1977: 84), Anuradhapura, Polonnaruwa (Dias and Kosgamage 2012: 63), Sri Lanka (Dias 2014: 195), Ihakuluweva (Dias and Peiris 2015a: 6), Anuradhapura, Kurunegala, Polonnaruwa, Puttalam (Dias and Rajapaksa 2016: 36), Indikada Mukalana Forest Reserve (Udayakantha and Dias 2018: 73); *Secondary literature records*: Sri Lanka (Forel 1904b: 175), Ceylon (Chapman and Capco 1951: 180), Sri Lanka (Ali 1992: 6), Sri Lanka (Mathew and Tiwari 2000: 310), Sri Lanka (Dias 2002: 18), Sri Lanka (Tiwari et al. 2003: 490), Sri Lanka (Dias 2006a: 52), Sri Lanka (Dias et al. 2012: 16); *AntWeb records*: Nuwara Eliya: CASENT 0280879, CASENT 0909193 (AntWeb 2020).

***Tetramorium transversarium* Roger, 1863**

*Tetramorium transversarium* Roger, 1863: 181. TL: [Ceylon] Sri Lanka [Syntypes: ZMHB (lost)].

**Distribution.** Wet Zone; *Primary literature records*: Ceylon (Roger 1863: 182), Nuwara Eliya (Emery 1893a: 246), Hakgala (Bolton 1977: 115); *Secondary literature records*: Ceylon (Forel 1903a: 702), Ceylon (Chapman and Capco 1951: 178), Sri Lanka (Dias 2002: 18), Sri Lanka (Dias 2006a: 52), Sri Lanka (Dias et al. 2012: 16).

***Tetramorium walshi* (Forel, 1890)**

*Triglyphothrix walshi* Forel, 1890: cvii. TL: [Bengalen (Pooree)], Bay of Bengal, Puri: India [Syntypes: MHNG, ZMHB]. [Images of CASENT 0909098, FOCOL 2061, 2062 syntype workers examined].

**Distribution.** Wet, Dry and Intermediate Zones; *Primary literature records*: Nawalapitiya (Emery 1893a: 248), Nawalapitiya (Forel 1903a: 704), Colombo, Gilimale, Nuwara Eliya (Bolton 1976: 359), Anuradhapura, Polonnaruwa (Dias and Kosgamage 2012: 63), Sri Lanka (Dias 2014: 195), Marawila, Madurankuliya (Dias and Peiris 2015a: 6), Mawathagama, Kurunegala (Dias and Peiris 2015b: 26), Anuradhapura, Colombo, Galle, Gampaha, Kurunegala, Polonnaruwa, Puttalam, Ratnapura (Dias and Rajapaksa 2016: 36), Indikada Mukalana Forest Reserve (Dias and Udayakantha 2016b: 5), Indikada Mukalana Forest Reserve (Udayakantha and Dias 2018: 73); *Secondary literature records*: Ceylon (Chapman and Capco 1951: 179), Sri Lanka (Ali 1992: 6), Sri Lanka (Mathew and Tiwari 2000: 299), Sri Lanka (Li-Zhong 2006: 273), Sri Lanka (Tak et al. 2007: 129), Sri Lanka (Sheela 2008a: 44), Sri Lanka (Tak 2008: 31), Sri Lanka (Tak and Kazmi 2011: 45), Sri Lanka (Dias et al. 2012: 16); *AntWeb records*: Waikkal, Ranweli: CASENT 0280877 (AntWeb 2020).

***Tetramorium yerburyi* Forel, 1902**

Tetramorium pilosum r. yerburyi Forel, 1902b: 238. TL: [Ceylon] Sri Lanka [Syntype: MHNG]. [Images of CASENT 0909188 syntype worker examined].

**Distribution.** Wet Zone; *Primary literature records*: Ceylon (Forel 1902b: 239), Ceylon (Forel 1903a: 703), Kandy (Bolton 1977: 85); *Secondary literature records*: Sri Lanka (Dias 2006a: 52), Sri Lanka (Dias et al. 2012: 17); *AntWeb records*: Sri Lanka: CASENT 0280880, CASENT 0909188 (AntWeb 2020).

#### *Trichomyrmex*: 8 species/subspecies

***Trichomyrmex aberrans* (Forel, 1902)**

*Monomorium aberrans* Forel, 1902b: 209. TL: Pachmarhi, Madhya Pradesh: India [Syntype: MHNG]. [Images of CASENT 0908722 syntype worker examined].

**Distribution.***AntWeb records*: Sri Lanka: CASENT 0914156 (Antweb 2020).

***Trichomyrmex criniceps* (Mayr, 1879)**

*Holcomyrmex criniceps* Mayr, 1879: 672. TL: [Tranquebar] Tharangambadi; Madras, Tamil Nadu: India [Syntypes: NHMW]. [Images of CASENT 0916013, 0916014 syntype workers examined].

**Distribution.** Wet and Dry Zones; *Primary literature records*: Ceylon (Forel 1903a: 693), Peradeniya (Forel 1911a: 221), Maha Iluppalama (Forel 1913a: 55), Anuradhapura, Polonnaruwa (Dias and Kosgamage 2012: 62), Anuradhapura, Polonnaruwa (Dias and Rajapaksa 2016: 36); *Secondary literature records*: Ceylon (Emery 1922: 181), Ceylon (Chapman and Capco 1951: 163), Ceylon (Ettershank 1966: 92), Sri Lanka (Ali 1992: 3), Ceylon (Radchenko 1997: 218), Sri Lanka (Tiwari 1999: 56), Sri Lanka (Dias 2002: 18), Sri Lanka (Tak and Rathore 2004a: 179), Sri Lanka (Dias 2006a: 51), Sri Lanka (Rajan et al. 2006: 177), Sri Lanka (Tak 2008: 21), Sri Lanka (Dias et al. 2012: 15); *AntWeb records*: Sri Lanka: CASENT 0908757 (AntWeb 2020).

***Trichomyrmex destructor* (Jerdon, 1851)**

*Atta destructor* Jerdon, 1851: 105. TL: Malabar, Kerala: India [Type: UNK].

**Distribution.** Wet, Dry and Intermediate Zones; *Primary literature records*: Colombo (Emery 1893a: 243), Ceylon (Emery 1901: 117), Colombo (Forel 1907a: 19), Ceylon (Forel 1909: 394), Ceylon (Forel 1911a: 221), Ceylon (Forel 1913a: 53), Colombo, Peradenya, Maha-Oyo (Bolton 1987: 325), South Sri Lanka (Way and Bolton 1997: 442), Kelaniya (Dias 2006a: 45), Sri Lanka (Heterick 2006: 96), Dambulla (Dias and Kosgamage 2008: 115), Sinharaja Forest Reserve (Gunawardene et al. 2008: 81), Sinharaja Forest Reserve (Gunawardene et al. 2012: 84), Anuradhapura, Polonnaruwa (Dias and Kosgamage 2012: 62), Sri Lanka (Dias 2014: 176), Namalweva, Ihakuluweva, Marawila, Madurankuliya, Egodayagama (Dias and Peiris 2015a: 6), Sinhapura, Polonnaruwa (Dias and Peiris 2015b: 26), Anuradhapura, Colombo, Galle, Gampaha, Kurunegala, Polonnaruwa, Puttalam, Ratnapura (Dias and Rajapaksa 2016: 36); *Secondary literature records*: Ceylon (Chapman and Capco 1951: 166), Sri Lanka (Dias 2002: 18), Sri Lanka (Tak and Rathore 2004a: 178), Sri Lanka (Dias 2006b: 8), Sri Lanka (Dias et al. 2012: 15); *AntWeb records*: Sri Lanka: CASENT 0010944, 0010945 (AntWeb 2020).

***Trichomyrmex emeryi laevior* (Mayr, 1897)**

*Monomorium emeryi laevior* Mayr, 1897: 427. TL: Kalawewa: [Ceylon] Sri Lanka [Syntype: NHMW?].

**Distribution.** Dry Zone; *Primary literature records*: Kalawewa (Mayr 1897: 427), Ceylon (Forel 1903a: 687); *Secondary literature records*: Ceylon (Emery 1922: 176), Ceylon (Chapman and Capco 1951: 166), Ceylon (Ettershank 1966: 90).

***Trichomyrmex glaber* (André, 1883)**

*Holcomyrmex glaber* André, 1883: 345. TL: India [Syntype: MNHN]. [Images of CASENT 0915415 syntype worker examined].

**Distribution.** Dry Zone; *Primary literature records*: Ceylon (Forel 1903a: 692), Ceylon (Forel 1909: 394), Maha Iluppalama (Forel 1913a: 55); *Secondary literature records*: Ceylon (Donisthorpe 1942b: 455), Ceylon (Chapman and Capco 1951: 163), Sri Lanka (Radchenko 1997: 221), Sri Lanka (Tiwari 1999: 57), Sri Lanka (Dias 2002: 18), Sri Lanka (Tak and Rathore 2004a: 180), Sri Lanka (Tak and Rathore 2004b: 87), Sri Lanka (Rajan et al. 2006: 178), Sri Lanka (Tak 2008: 22).

***Trichomyrmex mayri* (Forel, 1902)**

Monomorium gracillimum var. mayri Forel, 1902b: 209. TL: [Kelas Lake, Bombay] Mumbai: India [Lectotype: MHNG]. [Images of CASENT 0249904 lectotype worker examined].

**Distribution.** Wet Zone; *Primary literature records*: Peradenya (Bolton 1987: 326); *Secondary literature records*: Sri Lanka (Tak and Rathore 2004b: 88), Sri Lanka (Dias 2006a: 51), Sri Lanka (Tak 2008: 24), Sri Lanka (Tak 2010: 139), Sri Lanka (Dias et al. 2012: 15).

***Trichomyrmex rogeri* Mayr, 1865**

*Trichomyrmex rogeri* Mayr, 1865: 19. TL: [Ceylon] Sri Lanka [Holotype: NHMW]. [Images of CASENT 0916015, 0916016 syntype queens examined].

**Distribution.***Primary literature records*: Ceylon (Mayr 1865: 19), Ceylon (Forel 1903a: 699); *Secondary literature records*: Ceylon (Emery 1922: 186), Ceylon (Chapman and Capco 1951: 169), Ceylon (Ettershank 1966: 91), Sri Lanka (Radchenko 1997: 222), Sri Lanka (Dias 2002: 18), Sri Lanka (Dias 2006a: 51), Sri Lanka (Dias et al. 2012: 15); *AntWeb records*: Sri Lanka: CASENT 0916015, 0916016 (AntWeb 2020).

***Trichomyrmex wroughtoni* Forel, 1911**

*Trichomyrmex wroughtoni* Forel, 1911b: 453. TL: [Kanara], Karnataka: India [Syntypes: MHNG, NHMUK]. [Images of CASENT 0902224, CASENT 0908721 syntype workers examined].

**Distribution.***Primary literature records*: Sri Lanka (Radchenko 1997: 222).

#### *Tyrannomyrmex*: 1 species

***Tyrannomyrmex legatus* Alpert, 2013**

*Tyrannomyrmex legatus* Alpert, 2013: 287. TL: Sinharaja Forest Reserve: Sri Lanka [Holotype: MCZ]. [Images of CASENT 0106177 holotype worker examined].

**Distribution.** Wet Zone; *Primary literature records*: Sinharaja Forest Reserve (Alpert 2013: 287), Sri Lanka (Dias 2014: 196), Colombo, Galle, Gampaha, Ratnapura (Dias and Rajapaksa 2016: 37); *AntWeb records*: Sinharaja Forest Reserve: CASENT 0106177 (AntWeb 2020).

#### *Vollenhovia*: 1 species

***Vollenhovia escherichi* Forel, 1911**

*Vollenhovia escherichi* Forel, 1911e: 198. TL: Peradenyia [Ceylon] Sri Lanka [Syntype: MHNG]. [Images of CASENT 0908656 syntype worker examined].

**Distribution.** Wet Zone; *Primary literature records*: Peradenyia (Forel 1911e: 198); *Secondary literature records*: Ceylon (Emery 1922: 164), Ceylon (Chapman and Capco 1951: 170), Ceylon (Ettershank 1966: 149), Sri Lanka (Dias 2002: 18), Sri Lanka (Dias 2006a: 52), Sri Lanka (Dias et al. 2012: 17); *AntWeb records*: Peradenyia: CASENT 0908656 (AntWeb 2020).

### PONERINAE

#### *Anochetus*: 8 species

***Anochetus consultans* (Walker, 1859)**

*Formica consultans* Walker, 1859: 373. TL: [Ceylon] Sri Lanka [Holotype: NHMUK]. [Images of CASENT 0902446 holotype queen examined].

**Distribution.***Primary literature records*: Ceylon (Walker 1859: 373), Sri Lanka (Brown 1978: 556); *Secondary literature records*: Emery 1925: 271 (Ceylon), Sri Lanka (Dias 2006a: 52), Sri Lanka (Dias et al. 2012: 17); *AntWeb records*: Sri Lanka: CASENT 0902446 (AntWeb 2020).

***Anochetus graeffei* Mayr, 1870**

*Anochetus graeffei* Mayr, 1870: 961. TL: Samoa: Upolu Island [Lectotype: NHMW]. [Images of CASENT 0915887 lectoype worker examined].

**Distribution.** Wet, Dry and Intermediate Zones; *Primary literature records*: Ceylon (Emery 1911: 109), Paradeniya, Lady Black Drive (Forel 1911a: 215), Mihintale, Sinhapura (Dias and Kosgamage 2012: 63), Ambagaswewa (Shattuck and Slipinska 2012: 2), Sri Lanka (Dias 2014: 219), Egodayagama (Dias and Peiris 2015a: 6), Sinhapura, Polonnaruwa (Dias and Peiris 2015b: 26), Anuradhapura, Colombo, Galle, Gampaha, Kurunegala, Polonnaruwa, Puttalam, Ratnapura (Dias and Rajapaksa 2016: 37); *Secondary literature records*: Ceylon (Chapman and Capco 1951: 41), Sri Lanka (Ali 1991: 2), Sri Lanka (Dias 2002: 19), Sri Lanka (Sheela 2008a: 44), Sri Lanka (Dias et al. 2012: 17); *AntWeb records*: North Central: Ambagaswewa: ANIC 32-015987 (AntWeb 2020).

***Anochetus longifossatus* Mayr, 1897**

*Anochetus longifossatus* Mayr, 1897: 425. TL: Kalawewa: [Ceylon] Sri Lanka [Syntypes: MHNG, NHMW]. [Images of CASENT 0907405, CASENT 0915889 syntype workers examined].

**Distribution.** Wet, Dry and Intermediate Zones; *Primary literature records*: Kalawewa (Mayr 1897: 427), Kalawewa (Forel 1900a: 61), Peradeniya (Forel 1913a: 18), Kandy (Brown 1978: 593), Mihintale Forest, Pulliyarahandiya (Dias and Kosgamage 2012: 63), Sri Lanka (Dias 2014: 220), Wilpita “Aranya Kele” (Dias and Ruchirani 2014: 88), Anuradhapura, Colombo, Galle, Gampaha, Kurunegala, Polonnaruwa, Ratnapura (Dias and Rajapaksa 2016: 37); *Secondary literature records*: Ceylon (Emery 1911: 109), Ceylon (Chapman and Capco 1951: 40), Sri Lanka (Dias 2002: 19), Sri Lanka (Dias 2006a: 52), Sri Lanka (Dias et al. 2012: 17); *AntWeb records*: Ambagaswewa, Peradeniya, Bandarawela, Kandy, Kalawewa: ANIC 32-016936, 32-016937, CASENT 0281880, CASENT 0902444, CASENT 0907405, CASENT 0915889, FOCOL 1044 (AntWeb 2020) .

***Anochetus madaraszi* Mayr, 1897**

*Anochetus madaraszi* Mayr, 1897: 424. TL: Kalawewa: [Ceylon] Sri Lanka [Syntypes: NHMW]. [Images of CASENT 0915892, 0915893 syntype workers examined].

**Distribution.** Dry Zone; *Primary literature records*: Kalawewa (Mayr 1897: 425), Kalawewa (Forel 1900a: 61), Sri Lanka (Brown 1978: 557); *Secondary literature records*: Ceylon (Emery 1911: 109), Ceylon (Chapman and Capco 1951: 40), Sri Lanka (Tiwari et al. 1999: 232), Sri Lanka (Dias 2002: 19), Sri Lanka (Dias 2006a: 52), Sri Lanka (Dias et al. 2012: 17).

***Anochetus nietneri* (Roger, 1861)**

*Odontomachus nietneri* Roger, 1861: 23. TL: [Ceylon] Sri Lanka [Holotype: ZMHB].

**Distribution.** Wet, Dry and Intermediate Zones; *Primary literature records*: Ceylon (Roger 1861: 24), Ceylon (Forel 1900a: 60), Sri Lanka (Brown 1978: 558), Anuradhapura, Colombo, Galle, Gampaha, Kurunegala, Polonnaruwa, Ratnapura (Dias and Rajapaksa 2016: 37); *Secondary literature records*: Ceylon (Emery 1911: 109), Ceylon (Chapman and Capco 1951: 40), Sri Lanka (Dias 2002: 19), Sri Lanka (Dias 2006a: 52), Sri Lanka (Dias et al. 2012: 17); *AntWeb records*: Peradeniya, Kandy: ANIC 32-016952–6954 (AntWeb 2020).

***Anochetus obscurior* Brown, 1978**

*Anochetus obscurior* Brown, 1978: 558. TL: Madras: India [Syntype: MHNG]. [Images of CASENT 0907407 syntype worker examined].

**Distribution.** Wet Zone; *AntWeb records*: Colombo: CASENT 0281883 (AntWeb 2020).

***Anochetus pangens* (Walker, 1859)**

*Formica pangens* Walker, 1859: 371. TL: [Ceylon] Sri Lanka [Holotype: NHMUK]. [Images of CASENT 0902445 holotype male examined].

**Distribution.***Primary literature records*: Ceylon (Walker 1859: 371), Sri Lanka (Brown 1978: 558); *Secondary literature records*: Emery 1925: 271 (Ceylon), Ceylon (Chapman and Capco 1951: 200), Sri Lanka (Dias 2002: 19); *AntWeb records*: Sri Lanka: CASENT 0902445 (AntWeb 2020).

***Anochetus yerburyi* Forel, 1900**

*Anochetus yerburyi* Forel, 1900a: 62. TL: [Ceylon] Sri Lanka [Syntype: MHNG]. [Images of CASENT 0907416 syntype worker examined].

**Distribution.***Primary literature records*: Ceylon (Forel 1900a: 63), Sri Lanka (Brown 1978: 559); *Secondary literature records*: Ceylon (Emery 1911: 110), Ceylon (Chapman and Capco 1951: 42), Sri Lanka (Dias 2002: 19), Sri Lanka (Dias 2006a: 52) , Sri Lanka (Tak 2008: 13), Sri Lanka (Tak 2010: 136), Sri Lanka (Dias et al. 2012: 17); *AntWeb records*: Sri Lanka: CASENT 0907416 (AntWeb 2020).

#### *Bothroponera*: 3 species

***Bothroponera rubiginosa* (Emery, 1889)**

*Ponera rubiginosa* Emery, 1889: 498. TL: Tenasserim Moulmein: Myanmar [Syntype: MSNG]. [Images of CASENT 0903896 syntype worker examined].

**Distribution.** Dry Zone; *Primary literature records*: Sri Lanka (Dias et al. 2012: 18), Sinhapura, Polonnaruwa (Dias and Kosgamage 2012: 64).

***Bothroponera sulcata* (Mayr, 1867)**

*Ponera sulcata* Mayr, 1867: 441. TL: India [Syntype: NHMW]. [Images of CASENT 0915674 syntype worker examined].

**Distribution.** Wet Zone; *Primary literature records*: Sinharaja Forest Reserve (Gunawardene et al. 2008: 83), Sinharaja Forest Reserve (Gunawardene et al. 2012: 86); *Secondary literature records*: Sri Lanka (Dias et al. 2012: 18).

***Bothroponera tesseronoda* (Emery, 1877)**

*Ponera tesseronoda* Emery, 1877a: 368. TL: [Calcutta] Kolkata, West Bengal: India [Syntype: NHMW]. [Images of CASENT 0915675 syntype worker examined].

**Distribution.** Wet, Dry and Intermediate Zones; *Primary literature records*: Kandy (Emery 1893a: 242), Ceylon (Forel 1900b: 325), Ceylon (Emery 1901: 113), Up country (Forel 1908: 1), Dividosgala, Peradeniya (Forel 1911a: 216), Haputale, Maha Iluppalama (Forel 1913a: 7), Anuradhapura (Maschwitz et al. 1974: 113), Anuradhapura, Polonnaruwa (Dias and Kosgamage 2012: 64), Kirikanda Forest (Dias et al. 2013: 64), Namalweva, Ihakuluweva, Marawila, Pallama, Madurankuliya (Dias and Peiris 2015a: 6), Meethirigala Forest Reserve (Dias and Udayakantha 2016a: 53); *Secondary literature records*: Ceylon (Emery 1911: 78), Ceylon (Bingham 1903: 98), Ceylon (Donisthorpe 1942b: 449), Ceylon (Chapman and Capco 1951: 51), Sri Lanka (Ali 1991: 5), Sri Lanka (Tiwari 1999: 28), Sri Lanka (Tiwari et al. 1999: 234), Sri Lanka (Dias 2002: 19), Sri Lanka (Rajan et al. 2006: 187), Sri Lanka (Dias et al. 2012: 18).

#### *Brachyponera*: 3 species

***Brachyponera jerdonii* (Forel, 1900)**

*Ponera jerdonii* Forel, 1900b: 327. TL: Assam; Kerala: Calicut; Maharashtra: Pune; West Bengal: Kolkata, Barrackpore: India [Syntypes: MHNG]. [Images of CASENT 0907282 syntype worker examined].

**Distribution.** Wet Zone; *Primary literature records*: Sinharaja Forest Reserve (Gunawardene et al. 2008: 83), Indikada Mukalana Forest Reserve (Udayakantha and Dias 2018: 73).

***Brachyponera luteipes* (Mayr, 1862)**

*Ponera luteipes* Mayr, 1862: 722. TL: Andaman and Nicobar Islands: Nicobar Islands [Syntypes: MHNG]. [Images of CASENT 0915672 syntype worker examined].

**Distribution.** Wet Zone; *Primary literature records*: Kandy (Emery 1893a: 242), Ceylon (Forel 1900b: 326), Galle (Forel 1908: 1), Peradeniya (Forel 1911a: 216), Dompe (Forel 1913a: 8), Peradeniya (Karavaiev 1925a: 125), Peradeniya (Karavaiev 1926: 415), Sri Lanka (Dias 2014: 233), Kuluna Kanda Proposed Forest Reserve (Dias and Ruchirani 2014: 88), Indikada Mukalana Forest Reserve (Udayakantha and Dias 2018: 73); *Secondary literature records*: Ceylon (Emery 1911: 84), Ceylon (Wheeler 1921: 530), Ceylon (Chapman and Capco 1951: 63), Sri Lanka (Ali 1991: 4), Sri Lanka (Tiwari 1999: 31), Sri Lanka (Tiwari et al. 1999: 237), Sri Lanka (Mathew and Tiwari 2000: 287), Sri Lanka (Mathew 2003: 198), Sri Lanka (Dias 2006a: 52), Sri Lanka (Dias 2006b: 8), Sri Lanka (Ghosh et al. 2006: 376), Sri Lanka (Li-Zhong 2006: 263), Sri Lanka (Dias et al. 2012: 18).

***Brachyponera obscurans* (Walker, 1859)**

*Formica obscurans* Walker, 1859: 372. TL: [Ceylon] Sri Lanka [Holotype: NHMUK]. [Images of CASENT 0902498 holotype queen examined].

**Distribution.***Primary literature records*: Ceylon (Walker 1859: 372); *Secondary literature records*: Emery 1925: 271 (Ceylon), Ceylon (Chapman and Capco 1951: 200), Sri Lanka (Dias 2002: 19), Sri Lanka (Li-Zhong 2006: 263); *AntWeb records*: Sri Lanka: CASENT 0902498 (AntWeb 2020).

#### *Centromyrmex*: 2 species/subspecies

***Centromyrmex feae* (Emery, 1889)**

*Spalacomyrmex feae* Emery, 1889: 491. TL: Bhamò, Palon, [Pegù] Bago: [Birmania] Myanmar [Syntypes: MSNG, NHMW]. [Images of CASENT 0903860, CASENT 0915651 syntype workers examined].

**Distribution.** Wet and Dry Zones; *Primary literature records*: Kandy (Emery 1893a: 240), Kandy (Karavaiev 1925b: 81), Kandy (Karavaiev 1926: 415), Gilimale Forest Reserve (Dias and Perera 2011: 72), Sri Lanka (Dias et al. 2012: 17), Sri Lanka (Dias 2014: 222), Ihakuluweva (Dias and Peiris 2015a: 6), Anuradhapura, Colombo, Galle, Gampaha, Polonnaruwa, Ratnapura (Dias and Rajapaksa 2016: 37); *Secondary literature records*: Ceylon (Chapman and Capco 1951: 52), Sri Lanka (Ali 1991: 2), Sri Lanka (Tiwari et al. 1999: 280), Sri Lanka (Dias 2002: 19), Sri Lanka (Dias 2006a: 52), Sri Lanka (Li-Zhong 2006: 265).

***Centromyrmex feae ceylonicus* Forel, 1900**

Centromyrmex feae var. ceylonicus Forel, 1900b: 303. TL: [Ceylon] Sri Lanka [Syntype: MHNG]. [Images of CASENT 0907210 syntype worker examined].

**Distribution.** Wet Zone; *Primary literature records*: Ceylon (Forel 1900b: 303), Galle, Peradeniya (Forel 1911a: 215), Negombo (Forel 1913b: 660); *Secondary literature records*: Ceylon (Emery 1911: 58), Ceylon (Forel 1913a: 125), Ceylon (Chapman and Capco 1951: 52); *AntWeb records*: Sri Lanka: CASENT 0752148, CASENT 0907210 (AntWeb 2020).

#### *Cryptopone*: 1 species

***Cryptopone testacea* Emery, 1893**

*Cryptopone testacea* Emery, 1893b: cclxxv. TL: [Ceylon] Sri Lanka [Syntype: MSNG?].

**Distribution.** Wet Zone; *Primary literature records*: Nawalapitya (Emery 1893a: 241), Ceylon (Emery 1893b: cclxxv), Nawalapitya (Forel 1900b: 328), Nawalapitya (Wilson 1958a: 360), Sinharaja Forest Reserve (Gunawardene et al. 2008: 83), Sinharaja Forest Reserve (Gunawardene et al. 2012: 85); *Secondary literature records*: Ceylon (Emery 1911: 88), Nawalapitya (Wheeler 1933: 6), Ceylon (Donisthorpe 1942b: 451), Ceylon (Chapman and Capco 1951: 53), Sri Lanka (Dias 2002: 19), Sri Lanka (Dias 2006a: 52), Sri Lanka (Dias et al. 2012: 17).

#### *Diacamma*: 6 species/subspecies

***Diacamma ceylonense* Emery, 1897**

*Diacamma ceylonense* Emery, 1897b: 159. TL: [Ceylon] Sri Lanka [Syntype: MSNG]. [Images of CASENT 0903871 syntype worker examined].

**Distribution.** Wet and Dry Zones; *Primary literature records*: Ceylon (Emery 1897b: 159), Kelaniya, Gampaha (Dias 2006a: 46), Sri Lanka (Dias 2014: 227), Marawila, Pallama (Dias and Peiris 2015a: 6), Colombo, Galle, Gampaha, Puttalam, Ratnapura (Dias and Rajapaksa 2016: 37); *Secondary literature records*: Ceylon (Bingham 1903: 79), Ceylon (Emery 1911: 66), Ceylon (Chapman and Capco 1951: 57), Sri Lanka (Dias 2002: 19), Sri Lanka (Dias 2006b: 8), Sri Lanka (Dias et al. 2012: 17); *AntWeb records*: Sri Lanka: CASENT 0903871 (AntWeb 2020).

***Diacamma cyaneiventre* André, 1887**

*Diacamma cyaneiventre* André, 1887: 293. TL: Coimbatore, Tamil Nadu: India [Syntype: MNHN]. [Images of CASENT 0913724 syntype worker examined].

**Distribution.***Primary literature records*: Ceylon (Bingham 1903: 78); *Secondary literature records*: Ceylon (Emery 1911: 65), Ceylon (Chapman and Capco 1951: 54), Sri Lanka (Ali 1991: 2), Sri Lanka (Tiwari 1999: 26), Sri Lanka (Dias 2002: 19), Sri Lanka (Rajan et al. 2006: 185).

***Diacamma indicum* Santschi, 1920**

Diacamma rugosum var. indica Santschi, 1920: 179. TL: Kyd Island, Süd Andaman: India [Syntype: MHNG]. [Images of CASENT 0907229 syntype worker examined].

**Distribution.** Wet Zone; *Primary literature records*: Galle (Forel 1908: 1), Galle (Forel 1911a: 216), Sri Lanka (Viginier et al. 2004: 2096), Kirikanda Forest (Dias et al. 2013: 64), Sri Lanka (Dias 2014: 225), Colombo, Galle, Gampaha, Ratnapura (Dias and Rajapaksa 2016: 37); *Secondary literature records*: Ceylon (Chapman and Capco 1951: 59), Sri Lanka (Terayama et al. 2014).

***Diacamma rugosum* (Le Guillou, 1842)**

*Ponera rugosa* Le Guillou, 1842: 318. TL: Borneo, Sarawak, Malaysia [Syntype: MNHN]. [Images of CASENT 0913723 syntype worker examined].

**Distribution.** Wet and Intermediate Zones; *Primary literature records*: Colombo (Emery 1893a: 242), Ceylon (Emery 1897b: 153), Ceylon (Forel 1900b: 319), Ceylon (Emery 1901: 113), Kelaniya, Gampaha (Dias 2006a: 46), Nawalapitiya (Amarasinghe 2010: 12), Sri Lanka (Dias 2014: 226), Colombo, Galle, Gampaha, Kurunegala, Ratnapura (Dias and Rajapaksa 2016: 37); *Secondary literature records*: Ceylon (Chapman and Capco 1951: 55), Sri Lanka (Azuma and Kinjo 1987), Sri Lanka (Ali 1991: 2), Sri Lanka (Tiwari 1999: 25), Sri Lanka (Dias 2002: 19), Sri Lanka (Mathew and Tiwari 2000: 274), Sri Lanka (Tiwari and Tiwari 2002: 152), Sri Lanka (Mathew 2003: 196), Sri Lanka (Tiwari et al. 2003: 476), Sri Lanka (Dias 2006b: 8), Sri Lanka (Dias et al. 2012: 17); *AntWeb records*: Sri Lanka: FOCOL 0938–0940 (AntWeb 2020).

***Diacamma rugosum jerdoni* Forel, 1903**

Diacamma rugosum var. jerdoni Forel, 1903c: 401. TL: [Ceylon] Sri Lanka [Syntype: MHNG]. [Images of CASENT 0907222 syntype worker examined].

**Distribution.** Wet Zone; *Primary literature records*: Ceylon (Forel 1909: 392), Ambalagoda (Forel 1912c: 52), Seenigoda (Forel 1913a: 7); *Secondary literature records*: Ceylon (Emery 1911: 66), Ceylon (Donisthorpe 1942b: 449), Ceylon (Chapman and Capco 1951: 55); *AntWeb records*: Ceylon: CASENT 0907222 (AntWeb 2020).

***Diacamma rugosum sculptum* (Jerdon, 1851)**

*Ponera sculpta* Jerdon, 1851: 117. TL: Malabar, Kerala: India [Type: UNK].

**Distribution.***Primary literature records*: Ceylon (Emery 1897b: 157), Ceylon (Forel 1900b: 321), Ceylon (Emery 1901: 113); *Secondary literature records*: Ceylon (Bingham 1903: 80), Ceylon (Wheeler 1909: 338), Ceylon (Chapman and Capco 1951: 56), Sri Lanka (Tiwari 1999: 26), Sri Lanka (Tiwari et al. 1999: 234), Sri Lanka (Tiwari et al. 2003: 477).

#### *Harpegnathos*: 3 species/subspecies

***Harpegnathos saltator* Jerdon, 1851**

*Harpegnathos saltator* Jerdon, 1851: 117. TL: Malabar, Kerala: India [Type: UNK].

**Distribution.** Wet and Dry Zones; *Primary literature records*: Ceylon (Bingham 1903: 51), Sinharaja Forest Reserve (Gunawardene et al. 2008: 83), Mihintale Forest (Dias and Kosgamage 2012: 63), Sinharaja Forest Reserve (Gunawardene et al. 2012: 85), Sri Lanka (Dias 2014: 227), Anuradhapura, Polonnaruwa (Dias and Rajapaksa 2016: 37); *Secondary literature records*: Ceylon (Emery 1911: 59), Ceylon (Chapman and Capco 1951: 66), Sri Lanka (Ali 1991: 3), Sri Lanka (Tiwari 1999: 21), Sri Lanka (Dias 2002: 19), Sri Lanka (Dias 2006a: 52), Sri Lanka (Rajan et al. 2006: 185), Sri Lanka (Sheela 2008a: 47), Sri Lanka (Dias et al. 2012: 17).

***Harpegnathos saltator cruentatus* (Smith F, 1858)**

*Drepanognathus cruentatus* Smith F, 1858: 82. TL: Hong Kong: China [Holotype: NHMUK]. [Images of CASENT 0900659 holotype worker examined].

**Distribution.***Primary literature records*: Ceylon (Forel 1900a: 65); *Secondary literature records*: Ceylon (Donisthorpe 1937: 198).

***Harpegnathos saltator taprobanae* Forel, 1909**

Harpegnathos cruentatus var. taprobanae Forel, 1909: 392. TL: Dambulla: [Ceylon] Sri Lanka [Syntype: MHNG]. [Images of CASENT 0907213 syntype worker examined].

**Distribution.** Dry Zone; *Primary literature records*: Dambulla (Forel 1909: 392); *Secondary literature records*: Ceylon (Emery 1911: 59), Dambulla (Donisthorpe 1937: 198), Ceylon (Chapman and Capco 1951: 67); *AntWeb records*: Dambulla: CASENT 0907213 (AntWeb 2020).

#### *Hypoponera*: 8 species

***Hypoponera aitkenii* (Forel, 1900)**

Ponera confinis var. aitkenii Forel, 1900b: 325. TL: [Kanara], Karnataka: India [Syntypes: MHNG]. [Images of CASENT 0907305 syntype worker examined].

**Distribution.** Wet Zone; *Primary literature records*: Puwakpitiya (Forel 1908: 1), Peradeniya (Forel 1913a: 11); *Secondary literature records*: Ceylon (Chapman and Capco 1951: 69).

***Hypoponera bugnioni* (Forel, 1912)**

*Ponera bugnioni* Forel, 1912c: 52. TL: Ambalagoda: [Ceylon] Sri Lanka [Holotype: MHNG]. [Images of CASENT 0907301 syntype queen examined].

**Distribution.** Wet Zone; *Primary literature records*: Ambalagoda (Forel 1912c: 53); *AntWeb records*: Sri Lanka: CASENT 0907301 (AntWeb 2020).

***Hypoponera ceylonensis* (Mayr, 1897)**

*Ponera ceylonensis* Mayr, 1897: 422. TL: Kalawewa: [Ceylon] Sri Lanka [Syntype: HNHM]. [Images of CASENT 0922435 syntype worker examined].

**Distribution.** Dry Zone; *Primary literature records*: Kalawewa (Mayr 1897: 422), Ceylon (Forel 1900b: 325); *Secondary literature records*: Ceylon (Emery 1911: 90), Ceylon (Chapman and Capco 1951: 68), Sri Lanka (Dias 2002: 19), Sri Lanka (Dias 2006a: 52), Sri Lanka (Dias et al. 2012: 17); *AntWeb records*: Kalawewa: CASENT 0922435 (AntWeb 2020).

***Hypoponera confinis* (Roger, 1860)**

*Ponera confinis* Roger, 1860: 284. TL: [Ceylon] Sri Lanka [Syntype: ZMHB?].

**Distribution.** Wet Zone; *Primary literature records*: Ceylon (Roger 1860: 284), Kandy (Forel 1900b: 327), Peradeniya (Forel 1911a: 215), Peradeniya (Forel 1913a: 11), Kalugala Proposed Forest Reserve, Kuluna Kanda Proposed Forest Reserve, Wilpita “Aranya Kele” (Dias and Ruchirani 2014: 88), Colombo, Galle, Gampaha, Ratnapura (Dias and Rajapaksa 2016: 37), Indikada Mukalana Forest Reserve (Udayakantha and Dias 2018: 73); *Secondary literature records*: Ceylon (Emery 1895b: 295), Ceylon (Emery 1911: 90), Ceylon (Viehmeyer 1912: 18), Ceylon (Wheeler 1935: 12), Ceylon (Chapman and Capco 1951: 69), Ceylon (Wilson 1958a: 328), Sri Lanka (Tiwari 1999: 29), Sri Lanka (Tiwari et al. 1999: 243), Sri Lanka (Dias 2002: 19), Sri Lanka (Dias 2006a: 52), Sri Lanka (Li-Zhong 2006: 267), Sri Lanka (Dias et al. 2012: 17).

***Hypoponera punctatissima* (Roger, 1859)**

*Ponera punctatissima* Roger, 1859: 246. TL: [Rauden, Berlin], Rudy: Poland [Paralectotypes: DEIC, MNHN, ZMHB]. [Images of CASENT 0915490, FOCOL 0360, FOCOL 0981, 0982 paralectotype workers and queen examined].

**Distribution.** Sri Lanka (Museum of Comparative Zoology, Harvard).

***Hypoponera ragusai* (Emery, 1894)**

*Ponera ragusai* Emery, 1894: 28. TL: Sicily: Italy [Syntype: MSNG].

**Distribution.** Wet and Intermediate Zones; *Primary literature records*: Matale (Emery 1893a: 242), Matale (Forel 1900b: 327), Peradeniya (Forel 1911a: 215); *Secondary literature records*: Ceylon (Emery 1911: 91), Ceylon (Chapman and Capco 1951: 69), Sri Lanka (Dias 2002: 19), Sri Lanka (Li-Zhong 2006: 267), Sri Lanka (Bolton and Fisher 2011: 94).

***Hypoponera taprobanae* (Forel, 1913)**

*Ponera taprobanae* Forel, 1913a: 11. TL: Peradeniya: [Ceylon] Sri Lanka [Holotype: ZMHB]. [Images of FOCOL 0992 syntype worker examined].

**Distribution.** Wet Zone; *Primary literature records*: Peradeniya (Forel 1913a: 11); *Secondary literature records*: Ceylon (Chapman and Capco 1951: 73), Sri Lanka (Dias 2002: 19), Sri Lanka (Dias 2006a: 52), Sri Lanka (Dias et al. 2012: 17); *AntWeb records*: Peradeniya: FOCOL 0992 (AntWeb 2020).

***Hypoponera wroughtonii* (Forel, 1900)**

Ponera confinis var. wroughtonii Forel, 1900b: 325. TL: [Kanara], Karnataka: India [Syntypes: MHNG]. [Images of CASENT 0907308 syntype worker examined].

**Distribution.** Wet Zone; *Primary literature records*: Peradeniya (Forel 1911a: 215), Ceylon (Forel 1913a: 11).

#### *Leptogenys*: 11 species/subspecies

***Leptogenys chinensis* (Mayr, 1870)**

*Lobopelta chinensis* Mayr, 1870: 965. TL: China [Syntype: NHMW]. [Images of CASENT 0915873 syntype worker examined].

**Distribution.** Wet and Dry Zones; *Primary literature records*: Kandy (Emery 1893a: 242), Ceylon (Forel 1900b: 313), Anuradhapura (Emery 1901: 113), Ceylon (Forel 1909: 393), Indikada Mukalana Forest Reserve (Udayakantha and Dias 2018: 74); *Secondary literature records*: Ceylon (Emery 1911: 103), Ceylon (Chapman and Capco 1951: 33), Sri Lanka (Ali 1991: 3), Sri Lanka (Tiwari et al. 1999: 239), Sri Lanka (Dias 2002: 19), Sri Lanka (Tiwari and Tiwari 2002: 153), Sri Lanka (Li-Zhong 2006: 268), Sri Lanka (Sheela 2008a: 48), Sri Lanka (Zhou et al. 2012: 887), Sri Lanka (Xu and He 2015: 159).

***Leptogenys diminuta* (Smith F, 1857)**

*Ponera diminuta* Smith F, 1857: 69. TL: Sarawak: Malaysia [Syntype: OUMNH]. [Images of CASENT 0901351 syntype worker examined].

**Distribution.** Wet Zone; *Primary literature records*: Ceylon (Forel 1900b: 307); Bandarawela (Forel 1913b: 661), Kandy (Wilson 1958b: 120); *Secondary literature records*: Ceylon (Emery 1911: 103), Ceylon (Forel 1912c: 52), Ceylon (Chapman and Capco 1951: 33), Sri Lanka (Taylor 1988: 34), Sri Lanka (Tiwari et al. 1999: 239), Sri Lanka (Dias 2002: 19), Sri Lanka (Dias 2006a: 52), Sri Lanka (Li-Zhong 2006: 268), Sri Lanka (Dias et al. 2012: 18), Sri Lanka (Zhou et al. 2012: 888), Sri Lanka (Xu and He 2015: 138); *AntWeb records*: Sri Lanka: CASENT 0907370 (AntWeb 2020).

***Leptogenys diminuta laeviceps* (Smith F, 1857)**

*Ponera laeviceps* Smith F, 1857: 69. TL: Sarawak: Malaysia [Syntype: NHMUK]. [Images of CASENT 0902613 syntype worker examined].

**Distribution.** Wet Zone; *Primary literature records*: Kandy (Emery 1893a: 243), Bandarawela (Forel 1908: 2), Galle (Forel 1911a: 216).

***Leptogenys exundans* (Walker, 1859)**

*Formica exundans* Walker, 1859: 371. TL: [Ceylon] Sri Lanka [Syntype: NHMUK]. [Images of CASENT 0902621 syntype male examined].

**Distribution.***Primary literature records*: Ceylon (Walker 1859: 371); *Secondary literature records*: Emery 1925: 271 (Ceylon), Ceylon (Chapman and Capco 1951: 199), Sri Lanka (Dias 2002: 18), Sri Lanka (Dias 2006a: 52), Sri Lanka (Dias et al. 2012: 17); *AntWeb records*: Sri Lanka: CASENT 0902621 (AntWeb 2020).

***Leptogenys falcigera* Roger, 1861**

*Leptogenys falcigera* Roger, 1861: 42. TL: [Ceylon] Sri Lanka [Syntype: ZMHB]. [Images of CASENT 0104059 syntype worker examined].

**Distribution.** Wet Zone; *Primary literature records*: Ceylon (Roger 1861: 42), Ceylon (Forel 1900b: 309), Peradeniya (Bolton 1975: 253); *Secondary literature records*: Ceylon (Emery 1911: 99), Ceylon (Wheeler 1922: 1010), Ceylon (Chapman and Capco 1951: 31), Sri Lanka (Dias 2002: 19), Sri Lanka (Dias 2006a: 52), Sri Lanka (Dias et al. 2012: 18), Sri Lanka (Rakotonirina and Fisher 2014: 109), Sri Lanka (Xu and He 2015: 138); *AntWeb records*: Sri Lanka: CASENT 0104059 (AntWeb 2020).

***Leptogenys hysterica* Forel, 1900**

*Leptogenys hysterica* Forel, 1900b: 311. TL: Belgaum: India [Syntype: NHMB]. [Images of CASENT 0915229 syntype worker examined].

**Distribution.***Primary literature records*: Ceylon (Forel 1900b: 311); *Secondary literature records*: Ceylon (Emery 1911: 104), Ceylon (Chapman and Capco 1951: 35), Sri Lanka (Dias 2002: 19), Sri Lanka (Jaitrong and Nabhitabhata 2005: 25), Sri Lanka (Dias 2006a: 52), Sri Lanka (Dias et al. 2012: 17), Sri Lanka (Xu and He 2015: 156).

***Leptogenys meritans* (Walker, 1859)**

*Formica meritans* Walker, 1859: 371. TL: [Ceylon] Sri Lanka [Holotype: NHMUK]. [Images of CASENT 0902608 holotype male examined].

**Distribution.***Primary literature records*: Ceylon (Walker 1859: 371); *Secondary literature records*: Emery 1925: 271 (Ceylon), Ceylon (Chapman and Capco 1951: 200), Sri Lanka (Dias 2002: 18), Sri Lanka (Dias 2006a: 52), Sri Lanka (Dias et al. 2012: 18); *AntWeb records*: Sri Lanka: CASENT 0902608 (AntWeb 2020).

***Leptogenys peuqueti* (André, 1887)**

*Lobopelta peuqueti* André, 1887: 292. TL: Annam, Hué: Vietnam [Syntypes: MHNG, MNHN]. [Images of CASENT 0907380, CASENT 0915465 syntype workers examined].

**Distribution.** Wet and Dry Zones; *Primary literature records*: Kandy (Emery 1893a: 243), Ceylon (Forel 1900b: 314), Ceylon (Emery 1901: 113), Colombo (Forel 1907a: 7), Ceylon (Forel 1909: 393), Thulana (Dias and Kosgamage 2012: 64), Kirikanda Forest (Dias et al. 2013: 64), Sri Lanka (Dias 2014: 230), Anuradhapura, Colombo, Galle, Gampaha, Polonnaruwa, Ratnapura (Dias and Rajapaksa 2016: 37); *Secondary literature records*: Ceylon (Emery 1911: 104), Ceylon (Wheeler and Chapman 1925: 69), Ceylon (Menozzi 1932: 4), Ceylon (Chapman and Capco 1951: 38), Sri Lanka (Mathew and Tiwari 2000: 280), Sri Lanka (Dias 2002: 19), Sri Lanka (Dias 2006a: 52), Sri Lanka (Li-Zhong 2006: 268), Sri Lanka (Dias et al. 2012: 18), Sri Lanka (Zhou et al. 2012: 887), Sri Lanka (Xu and He 2015: 160).

***Leptogenys processionalis* (Jerdon, 1851)**

*Ponera processionalis* Jerdon, 1851: 118. TL: Malabar, Kerala: India [Type: UNK].

**Distribution.** Wet, Dry and Intermediate Zones; *Primary literature records*: Ceylon (Roger 1861: 14), Kandy (Emery 1893a: 243), Ceylon (Forel 1900b: 309), Ceylon (Emery 1901: 113), Puwakpitiya, Pattipola (Forel 1908: 1), Peradeniya (Forel 1911a: 216), Maha Iluppalama (Forel 1913a: 16), Sinharaja Forest Reserve (Gunawardene et al. 2012: 86), Kirikanda Forest (Dias et al. 2013: 64), Anuradhapura, Polonnaruwa (Dias and Kosgamage 2012: 63), Sri Lanka (Dias 2014: 231), Kalugala Proposed Forest Reserve (Dias and Ruchirani 2014: 88), Namalweva (Dias and Peiris 2015a: 6), Anuradhapura, Colombo, Galle, Gampaha, Kurunegala, Polonnaruwa, Puttalam, Ratnapura (Dias and Rajapaksa 2016: 37); *Secondary literature records*: Ceylon (Emery 1911: 104), Ceylon (Donisthorpe 1942b: 452), Ceylon (Chapman and Capco 1951: 38), Sri Lanka (Ali 1991: 4), Sri Lanka (Tiwari 1999: 22), Sri Lanka (Tiwari et al. 1999: 241), Sri Lanka (Dias 2002: 19), Sri Lanka (Tiwari and Tiwari 2002: 153), Sri Lanka (Tak and Rathore 2004a: 164), Sri Lanka (Dias 2006a: 52), Sri Lanka (Dias 2006b: 8), Sri Lanka (Sheela 2008a: 49), Sri Lanka (Tak 2008: 13), Sri Lanka (Dias et al. 2012: 17), Sri Lanka (Xu and He 2015: 142).

***Leptogenys pruinosa* Forel, 1900**

*Leptogenys pruinosa* Forel, 1900b: 304. TL: [Ceylon] Sri Lanka [Syntype: MHNG]. [Images of CASENT 0907337 syntype worker examined].

**Distribution.** Wet, Dry and Intermediate Zones; *Primary literature records*: Ceylon (Forel 1900b: 304), Anuradhapura, Polonnaruwa (Dias and Kosgamage 2012: 64), Sri Lanka (Dias 2014: 231), Anuradhapura, Colombo, Galle, Gampaha, Kurunegala, Polonnaruwa, Ratnapura (Dias and Rajapaksa 2016: 37); *Secondary literature records*: Ceylon (Emery 1911: 99), Ceylon (Wheeler and Chapman 1925: 69), Ceylon (Chapman and Capco 1951: 31), Sri Lanka (Dias 2002: 19), Sri Lanka (Dias 2006a: 52), Sri Lanka (Dias et al. 2012: 17), Sri Lanka (Xu and He 2015: 142); *AntWeb records*: Karataivu Island: CASENT 0281920, CASENT 0907337 (AntWeb 2020).

***Leptogenys yerburyi* Forel, 1900**

*Leptogenys yerburyi* Forel, 1900b: 311. TL: [Ceylon] Sri Lanka [Syntype: MHNG]. [Images of CASENT 0907384 syntype worker examined].

**Distribution.** Wet Zone; *Primary literature records*: Ceylon (Forel 1900b: 311), Dividsogala, Peradeniya (Forel 1911a: 216); *Secondary literature records*: Ceylon (Emery 1911: 105), Sri Lanka (Dias 2002: 19), Sri Lanka (Dias 2006a: 52), Sri Lanka (Dias et al. 2012: 17), Ceylon (Xu and He 2015: 142); *AntWeb records*: Sri Lanka: CASENT 0907384 (AntWeb 2020).

#### *Mesoponera*: 1 species

***Mesoponera melanaria* (Emery, 1893)**

*Ponera melanaria* Emery, 1893d: 260. TL: Colombo: [Ceylon] Sri Lanka; Nadungayam, Malabar: India [Syntype: MSNG, NHMUK]. [Images of CASENT 0903930, CASENT 0902489 syntype worker and male examined].

**Distribution.** Wet, Dry and Intermediate Zones; *Primary literature records*: Colombo (Emery 1893a: 242), Ceylon (Emery 1893d: 260), Ceylon (Forel 1900b: 326), Peradeniya (Forel 1911a: 216), Peradeniya (Forel 1913a: 8), Sinharaja Forest Reserve (Gunawardene et al. 2008: 83), Sinharaja Forest Reserve (Gunawardene et al. 2012: 86), Colombo, Galle, Gampaha, Kurunegala, Puttalam, Ratnapura (Dias and Rajapaksa 2016: 37), Indikada Mukalana Forest Reserve (Dias and Udayakantha 2016b: 5), Indikada Mukalana Forest Reserve (Udayakantha and Dias 2018: 74); *Secondary literature records*: Ceylon (Emery 1911: 81), Ceylon (Wheeler 1922: 1008), Ceylon (Chapman and Capco 1951: 64), Sri Lanka (Ali 1991: 4), Sri Lanka (Tiwari 1999: 31), Sri Lanka (Dias 2002: 19); *AntWeb records*: Colombo: CASENT 0903930 (AntWeb 2020).

#### *Myopias*: 1 species

***Myopias amblyops* Roger, 1861**

*Myopias amblyops* Roger, 1861: 39. TL: [Ceylon] Sri Lanka [Syntype: ZMHB]. [Images of CASENT 0104584 syntype worker examined].

**Distribution.** Wet Zone; *Primary literature records*: Ceylon (Roger 1861: 40) (Forel 1900b: 328), Sinharaja Forest Reserve (Gunawardene et al. 2008: 83); *Secondary literature records*: Ceylon Ceylon (Emery 1911: 94), Ceylon (Chapman and Capco 1951: 67), Sri Lanka (Dias 2002: 19), Sri Lanka (Dias 2006a: 52), Sri Lanka (Dias et al. 2012: 18), Sri Lanka (Xu et al. 2014: 165); *AntWeb records*: Sri Lanka: CASENT 0104584 (AntWeb 2020).

#### *Odontomachus*: 1 species

***Odontomachus simillimus* Smith F, 1858**

*Odontomachus simillimus* Smith F, 1858: 80. TL: Oceania, Navai: Fiji; [Ceylon] Sri Lanka [Syntypes: NHMUK]. [Images of CASENT 0900649, CASENT 0900650 syntype queens examined].

**Distribution.** Wet, Dry and Intermediate Zones; *Primary literature records*: Ceylon (Smith, F. 1858: 80), Peradeniya (Forel 1913a: 19), Kandy (Karavaiev 1925c: 295), Kandy (Karavaiev 1926: 417), Kelaniya, Gampaha (Dias 2006a: 46), Sinharaja Forest Reserve (Gunawardene et al. 2008: 83), Gilimale Forest Reserve (Dias and Perera 2011: 72), Sri Lanka (Sorger and Zettel 2011: 160), Kirikanda Forest (Dias et al. 2013: 64), Sri Lanka (Dias 2014: 234), Kalugala Proposed Forest Reserve, Kuluna Kanda Proposed Forest Reserve, Wilpita “Aranya Kele” (Dias and Ruchirani 2014: 88), Campus of University Peradeniya, Kandy, Ratnapura, Pilikuttuwa (Satria et al. 2015: 6), Meethirigala Forest Reserve (Udayakantha and Dias 2015: 31), Colombo, Galle, Gampaha, Kurunegala, Puttalam, Ratnapura (Dias and Rajapaksa 2016: 37), Meethirigala Forest Reserve (Dias and Udayakantha 2016a: 53), Indikada Mukalana Forest Reserve (Dias and Udayakantha 2016b: 5), Indikada Mukalana Forest Reserve (Udayakantha and Dias 2018: 74); *Secondary literature records*: Ceylon (Chapman and Capco 1951: 46), Sri Lanka (Ali 1991: 4), Sri Lanka (Dias 2006b: 8), Sri Lanka (Dias et al. 2012: 18); *AntWeb records*: Peradenyia, Colombo: CASENT 0900650, ANIC 32-031797, 32-031798, FOCOL 1049–1051 (AntWeb 2020).

#### *Parvaponera*: 1 species

***Parvaponera darwinii* (Forel, 1893)**

*Belonopelta darwinii* Forel, 1893c: 460. TL: Port Darwin, Northern Territory: Australia [Syntype: MHNG]. [Images of CASENT 0907290 syntype queen examined].

**Distribution.** Wet Zone; *Primary literature records*: Ceylon (Emery 1901: 113), Ambalangoda (Forel 1909: 393); *Secondary literature records*: Ceylon (Bingham 1903: 93), Ceylon (Wheeler 1922: 1009), Ceylon (Chapman and Capco 1951: 65), Sri Lanka (Tiwari 1999: 30), Sri Lanka (Dias 2002: 19); *AntWeb records*: Dambuwa: CASENT 0810341, CASENT 0810343, CASENT 0810345, 0810346, CASENT 0810350, 0810351 (AntWeb 2020).

#### *Platythyrea*: 2 species

***Platythyrea clypeata* Forel, 1911**

*Platythyrea clypeata* Forel, 1911d: 378. TL: Pays de Moïs (the Cochin Chine Francaise): Vietnam [Syntype: MHNG]. [Images of CASENT 0907112 syntype queen examined].

**Distribution.***Primary literature records*: Ceylon (Donisthorpe 1931: 496), Ceylon (Brown 1975: 10); *Secondary literature records*: Ceylon (Chapman and Capco 1951: 49), Sri Lanka (Xu and Zeng 2000: 214), Sri Lanka (Dias 2002: 19), Sri Lanka (Dias 2006a: 52), Sri Lanka (Dias et al. 2012: 18); *AntWeb records*: Sri Lanka: CASENT 0900568 (AntWeb 2020).

***Platythyrea parallela* (Smith F, 1859)**

*Ponera parallela* Smith F, 1859: 143. TL: Aru I.: Indonesia [Holotype: OUMNH]. [Images of CASENT 0102936 holotype worker examined].

**Distribution.** Wet and Dry Zones; *Primary literature records*: Ceylon (Forel 1909: 393), Ceylon (Donisthorpe 1941: 203), Yakkala (Brown 1975: 49), Pohoranwewa, Dambulla (Dias and Kosgamage 2012: 64), Sri Lanka (Dias 2014: 235), Anuradhapura, Colombo, Galle, Gampaha, Polonnaruwa, Ratnapura (Dias and Rajapaksa 2016: 37); *Secondary literature records*: Ceylon (Chapman and Capco 1951: 47), Sri Lanka (Dias 2002: 19), Sri Lanka (Dias 2006a: 52), Sri Lanka (Dias 2006b: 8), Sri Lanka (Dias et al. 2012: 18); *AntWeb records*: Yakkala: CASENT 0900570, CASENT 0915904, ANIC 32-065987 (AntWeb 2020).

#### *Pseudoneoponera*: 3 species/subspecies

***Pseudoneoponera insularis* (Emery, 1889)**

*Bothroponera insularis* Emery, 1889: 495. TL: Java, Ardjoeno: Indonesia [Syntype: MSNG]. [Images of CASENT 0903889 syntype worker examined].

**Distribution.***Primary literature records*: Ceylon (Forel 1903a: 713); *Secondary literature records*: Ceylon (Chapman and Capco 1951: 50).

***Pseudoneoponera rufipes* (Jerdon, 1851)**

*Ponera rufipes* Jerdon, 1851: 119. TL: Malabar, Kerala: India [Type: UNK].

**Distribution.** Wet Zone; *Primary literature records*: Ceylon (Forel 1900b: 326), Kandy (Bingham 1903: 97), Puwakpitiya (Forel 1908: 1), Peradeniya (Forel 1911a: 216), Sinharaja Forest Reserve (Gunawardene et al. 2008: 83), Sinharaja Forest Reserve (Gunawardene et al. 2012: 86), Kuluna Kanda Proposed Forest Reserve (Dias and Ruchirani 2014: 88), Colombo, Galle, Gampaha, Ratnapura (Dias and Rajapaksa 2016: 37), Indikada Mukalana Forest Reserve (Dias and Udayakantha 2016b: 5), Indikada Mukalana Forest Reserve (Udayakantha and Dias 2018: 74); *Secondary literature records*: Ceylon (Emery 1911: 76), Ceylon (Wheeler 1921: 529), Kandy (Mukherji and Ribeiro 1925: 206), Sri Lanka (Ali 1991: 5), Sri Lanka (Tiwari 1999: 28), Sri Lanka (Tiwari et al. 1999: 233), Sri Lanka (Dias 2002: 19), Sri Lanka (Mathew and Tiwari 2000: 288), Sri Lanka (Tiwari and Tiwari 2002: 153), Sri Lanka (Mathew 2003: 199), Sri Lanka (Tiwari et al. 2003: 478), Sri Lanka (Ghosh et al. 2006: 377), Sri Lanka (Li-Zhong 2006: 269), Sri Lanka (Sheela 2008a: 51).

***Pseudoneoponera rufipes ceylonensis* (Forel, 1911)**

Pachycondyla rufipes subsp. ceylonensis Forel, 1911a: 216. TL: Peradenyia: [Ceylon] Sri Lanka [Syntype: MHNG]. [Images of CASENT 0907254 syntype worker examined].

**Distribution.** Wet Zone; *Primary literature records*: Ceylon (Emery 1911: 77), Peradeniya (Forel 1911a: 216); *Secondary literature records*: Ceylon (Chapman and Capco 1951: 50); *AntWeb records*: Peradenyia: CASENT 0907254 (AntWeb 2020).

### PROCERATIINAE

#### *Discothyrea*: 1 species

***Discothyrea* sp.**

**Distribution.** Wet Zone; *Primary literature records*: Sinharaja Forest Reserve (Gunawardene et al. 2008: 75), Sinharaja Forest Reserve (Gunawardene et al. 2012: 85), Sinharaja Forest Reserve (Gunawardene et al. 2010: 558).

### PSEUDOMYRMECINAE

#### *Tetraponera*: 4 species

***Tetraponera allaborans* (Walker, 1859)**

*Pseudomyrma allaborans* Walker, 1859: 375. TL: [Ceylon] Sri Lanka [Syntypes: NHMUK]. [Images of CASENT 0902822, 0902823 syntype worker and queen examined].

**Distribution.** Wet, Dry and Intermediate Zones; *Primary literature records*: Ceylon (Walker 1859: 375), Kandy, Colombo (Emery 1893a: 243), Ceylon (Emery 1901: 113), Ceylon (Forel 1903a: 710), Galle (Forel 1908: 3), Ambalangoda, Colombo (Forel 1909: 395), Peradeniya (Forel 1911a: 226), Montagnes de Nuwara Eliya, Kandy, Udawattakele, Peradeniya, Lunuwila, Ambalantota, Galle, Uva, Bibile, Uva Egodapitiya Nilgala, Labugama, Colombo, Laxapathiya, Yakkala (Ward 2001: 602) , Ratnapura (Dias 2006a: 46), Dambulla (Dias and Kosgamage 2008: 115), Gilimale Forest Reserve (Dias and Perera 2011: 73), Anuradhapura, Polonnaruwa (Dias and Kosgamage 2012: 64), Sri Lanka (Dias 2014: 249), Anuradhapura, Colombo, Galle, Gampaha, Kurunegala, Polonnaruwa, Puttalam, Ratnapura (Dias and Rajapaksa 2016: 37), Indikada Mukalana Forest Reserve (Udayakantha and Dias 2018: 74); *Secondary literature records*: Ceylon (Viehmeyer 1912: 19), Ceylon (Chapman and Capco 1951: 78), Sri Lanka (Ali 1992: 1), Ceylon (Radchenko 1993: 76), Sri Lanka (Tiwari 1999: 34), Sri Lanka (Tiwari et al. 1999: 247), Sri Lanka (Mathew and Tiwari 2000: 294), Sri Lanka (Dias 2002: 17), Sri Lanka (Tiwari and Tiwari 2002: 154), Sri Lanka (Mathew 2003: 199), Sri Lanka (Jaitrong and Nabhitabhata 2005: 44), Sri Lanka (Li-Zhong 2006: 273), Sri Lanka (Dias 2006b: 8), Sri Lanka (Rajan et al. 2006: 188), Sri Lanka (Dias et al. 2012: 18); *AntWeb records*: Egodapitiya, Nilgala, Colombo, Kandy, Laxapathiya, Yakkala, Ambalangoda: CASENT 0902822, 0902823, CASENT 0103238, CASENT 0752591, CASENT 0761602, 0761603, 0761604, CASENT 0795865, 0795866, 0795867, CASENT 0795958, 0795959, CASENT 0907456, SAM-HYM-C 006004 (AntWeb 2020).

***Tetraponera nigra* (Jerdon, 1851)**

*Eciton nigrum* Jerdon, 1851: 112. TL: Malabar, Kerala: India [Type: UNK].

**Distribution.** Wet and Dry Zones; *Primary literature records*: Kandy (Emery 1893a: 243), Ceylon (Emery 1901: 113), Ceylon (Forel 1903a: 709), Ceylon (Forel 1908: 3), Ceylon (Forel 1909: 394), Peradeniya (Forel 1911a: 226), Ceylon (Forel 1913a: 27), Dambulla, Kandy, Peradeniya, Anuradhapura, Polonnaruwa, Mahaoya, Ruhunu Natl Pk, Yala, Uva, Bibile, Egodapitiya Nilgala, Padukka Group Estate, Yakkala (Ward 2001: 635), Mihintale Forest (Dias and Kosgamage 2012: 64), Sri Lanka (Dias 2014: 252), Colombo, Galle, Gampaha, Ratnapura (Dias and Rajapaksa 2016: 37); *Secondary literature records*: Ceylon (Donisthorpe 1942b: 454), Ceylon (Chapman and Capco 1951: 80), Sri Lanka (Ali 1992: 1), Sri Lanka (Tiwari 1999: 35), Sri Lanka (Tiwari et al. 1999: 348), Sri Lanka (Dias 2002: 17), Sri Lanka (Tiwari et al. 2003: 481), Sri Lanka (Dias 2006a: 52), Sri Lanka (Li-Zhong 2006: 273), Sri Lanka (Sheela 2008a: 52), Sri Lanka (Dias et al. 2012: 18); *AntWeb records*: Egodapitiya, Padukka, Peradeniya, Yala, Yakkala, Kandy, Hatton, Puttalam: CASENT 0752658, 0752659, CASENT 0761626, CASENT 0796287–0796291, CASENT 0796626, CASENT 0902830, CASENT 0904036, FOCOL 0320–0323 (AntWeb 2020).

***Tetraponera nitida* (Smith F, 1860)**

*Pseudomyrma nitida* Smith F, 1860: 106a. TL: [Bac.] Bacan, Maluku Utara: Indonesia [Holotype: OUMNH]. [Images of CASENT 0901931 holotype worker examined].

**Distribution.** Wet Zone; *Primary literature records*: Kandy, Dehiwala, Yakkala (Ward 2001: 637); *AntWeb records*: Yakkala: CASENT 0796301 (AntWeb 2020).

***Tetraponera rufonigra* (Jerdon, 1851)**

*Eciton rufonigrum* Jerdon, 1851: 111. TL: Malabar, Kerala: India [Type: UNK].

**Distribution.** Wet, Dry and Intermediate Zones; *Primary literature records*: Ceylon (Emery 1901: 113), Ceylon (Forel 1909: 394), Ceylon (Forel 1912d: 83), eastern Sri Lanka, Pushparanghnam Estate, Sri Lanka Cashew Corporation-West, western Sri Lanka (Rickson and Rickson 1998: 843), Kandalama, Nalanda, Anuradhapura, Medirigiriya, nr Polonnaruwa, Mihintale, Polonnaruwa, Jaffna, Kantale, Moneragala, Mahaoya Dist., Paraiyanalankulam, Medawachchiya, Kadaimparu, Lunuwila, Sabaragamuwa, Uggalkaltota, Lunuwila, Walawe Ganga, Ratnapura, Hambantota, Hikkaduwa Yala, Ruhuna Natl Pk, Palatupana, Uva, Dunhinda Falls, Uda Walawe, Beruwala, Bentota (Ward 2001: 649), Kelaniya, Colombo, Ratnapura, Galle (Dias 2006a: 46), Dambulla (Dias and Kosgamage 2008: 115), Anuradhapura, Polonnaruwa (Dias and Kosgamage 2012: 64), Sri Lanka (Dias 2014: 254), Anuradhapura, Colombo, Galle, Gampaha, Kurunegala, Polonnaruwa, Puttalam, Ratnapura (Dias and Rajapaksa 2016: 37); *Secondary literature records*: Ceylon (Forel 1903a: 709), Ceylon (Donisthorpe 1942b: 454), Ceylon (Chapman and Capco 1951: 81), Sri Lanka (Ali 1992: 1); Sri Lanka (Tiwari 1999: 35), Sri Lanka (Tiwari et al. 1999: 248), Sri Lanka (Mathew 2000: 349), Sri Lanka (Mathew and Tiwari 2000: 295), Sri Lanka (Dias 2002: 17), Sri Lanka (Tiwari and Tiwari 2002: 154), Sri Lanka (Mathew 2003: 199), Sri Lanka (Tak and Rathore 2004a: 174), Sri Lanka (Tiwari et al. 2004: 609), Sri Lanka (Dias 2006b: 8), Sri Lanka (Ghosh et al. 2006: 384), Sri Lanka (Rajan et al. 2006: 188), Sri Lanka (Sheela 2008a: 54), Sri Lanka (Tak 2008: 14), Sri Lanka (Tak 2010: 136), Sri Lanka (Tak and Kazmi 2011: 41), Sri Lanka (Dias et al. 2012: 18); *AntWeb records*: Kandy, Anuradhapura, Polonnaruwa, Paraiyanalankulam, Kandalama, Beruwala, Hikkaduwa Yala: CASENT 0761638, CASENT 0796751–0796757, CASENT 0862081, 0862082, CASENT 0907449 (AntWeb 2020).

## Supplementary materials

Supplementary material 1 Ant diversity across three zones of Sri Lanka, endemics, invasive species, and dubious taxa Data type data set https://binary.pensoft.net/file/452587This dataset is made available under the Open Database License (http://opendatacommons.org/licenses/odbl/1.0/). The Open Database License (ODbL) is a license agreement intended to allow users to freely share, modify, and use this Dataset while maintaining this same freedom for others, provided that the original source and author(s) are credited.Ratnayake Kaluarachchige Sriyani Dias, Benoit Guénard, Shahid Ali Akbar, Evan P. Economo, Warnakulasuriyage Sudesh Udayakantha, Aijaz Ahmad Wachkoo

Supplementary material 2 Localities in Sri Lanka sampled for ants, across three zones Data type occurrences https://binary.pensoft.net/file/452588This dataset is made available under the Open Database License (http://opendatacommons.org/licenses/odbl/1.0/). The Open Database License (ODbL) is a license agreement intended to allow users to freely share, modify, and use this Dataset while maintaining this same freedom for others, provided that the original source and author(s) are credited.Ratnayake Kaluarachchige Sriyani Dias, Benoit Guénard, Shahid Ali Akbar, Evan P. Economo, Warnakulasuriyage Sudesh Udayakantha, Aijaz Ahmad Wachkoo
