# Supplementary material for: The Ants (Hymenoptera, Formicidae) of Sri Lanka: a taxonomic research summary and updated checklist
Source: Zookeys. 2020 Sep 14;967:1–142. doi: 10.3897/zookeys.967.54432 (PMC7508952; doi:10.3897/zookeys.967.54432)
Supplement: Supplementary material 2 — Localities in Sri Lanka sampled for ants, across three zones [file zookeys-967-001-s002.docx]

**Appendix I. Localities in Sri Lanka sampled for ants, across three zones. Misspellings/other spellings are provided within parenthesis ().**

| **Wet Zone** | **Dry Zone** | **Intermediate Zone** |
| --- | --- | --- |
| **Bandarawela District**  Bandarawela (Bandarawella, Bandaravella)  **Colombo District**  Colombo (Columbo)  Dehiwala  Labukelle (Labukele)  Laxapathiya (Laxapathiye)  Kottawa (Cattawa, Cottawa, Kattawa)  Labugama  Moratuwa  Padukka Group/Rubber Estate  Panadura Estate (Pandura, Penadura)  Puwakpitiya (Puwakpitya)  Ratmalana  Sri Lanka Cashew Corporation, Jayawardenepura  Waga Forest  **Gampaha District**  Dadagamuwa  Henarathgoda (Henaratgoda)  Indikada Mukalana Forest Reserve  Katunayake (Katunayaka)  Kadaimparu  Kelaniya (Kanneliya Jungle, Kellany Valley)  Maimbula (Mainbula) Forest Reserve  Meethirigala Forest Reserve  Negombo  Pilikuttuwa Forest  Seeduwa  Watinapaha  Yakkala  **Galle District**  Ambalangoda  Bentota (Benthota)  Danawala  Deniyaya  Induruwa Jungle  Pointe de Galle  Seenigoda  Wackwella (Wackwell)  **Kalutara District**  Beruwala (Benuwala)  Gonapola  Hikkaduwa  Kalugala Proposed Forest Reserve  Kalutara  Kirikanda Forest  Kuluna Kanda Proposed Forest Reserve  **Kandy District**  Dambuwa Estate  Dividosgala (DividosGalla)  Gannoruwa Forest  Hantana  Hasalaka  Kandy  Kataluoya Estate  Nawalapitiya  Peradeniya (Peraden)  Randenigala  Rantembe Sanctuary  Udawattakele Sanctuary (Udawaddatekele Sanctuary)  Victoria  **Kegalle District**  Lenagala Forest Reserve  **Matara District**  Wilpita “Aranya Kele”  Weligama  **Nuwara Eliya District**  Boragas (Coragas)  Hakgala (Hackgalla, Hargala, Hakgalam)  Hatton  Horton Plains  Montagnes de Nuwara-Eliya  Nuwara Eliya (nrEliy, Nura Ellia)  Pattipola  Pidurutalagala (Pidacatalagata)  Pundaluoya (Pundulaoya, Pundula-ya)  Pushparanghnam Estate  Ramboda (Rambodda, Rambode)  **Ratnapura District**  Belihuloya (Belihulaya)  Dompe (Dampe, Lampe)  Gilimale Forest Reserve  Gilimale  Kalthota (Ngyalkaltota)  Opanayaka (Opanyeka)  Pompekelle  Ratnapura  Ratgama (Ratgama-Kellei)  Sinharaja Forest Reserve  Sugarcane Research Institute, Uda Walawe  Walawe Ganga  Uggalkaltota  Uggalkaltota Irrigation Bungalow  Udawalawe  Udugala (Udugalla)  Udugama | **Anuradhapura District**  Anuradhapura (Anuradbapura)  Dambulla (Bambulla)  Ihakuluwewa  Kahalla-Pallekele Forest (Kahall-Pallekel Forest)  Kalawewa (Kalawera)  Kandalama  Kawarakkulama  Mahaoya  Mahawa (Maho)  Maha Iluppallama (MahaIluppalama Farm, Maha Illupullansa Res. Farm)  Medawachchiya  Mihintale  Nachchaduwa (Nachchaduwa Wewa)  Namalweva  Nikawewa  Thulana  **Hambanthota District**  Ambalantota  Hambantota  Lunugamvehera (Lunugamehera), near Wirawila  Palatupana  Ruhunu National Park  Sitrakala  Weerawila  Yala  Yala National Park  **Jaffna District**  Karataivu Island  Jaffna  **Mannar District**  Paraiyanalankulam  **Monaragala District**  Bibile  Inginiyagala  Moneragala (Nunaragala)  Nilgala  Wellawaya  **Polonnaruwa District**  Aluthoya (Alut Oya, Alur Oya)  Ambagaswewa  Giritale Forest  Hathamuna  Jayanthipura  Minneriya  Medirigiriya (Madirigiriya)  Nagalakanda Forest  Pohoranwewa  Polonnaruwa (Pollonnaruwa, Pollonaruwa)  Pulathisigama  Pulliyarahandiya  Sinhapura  Somawathiya Sanctuary  **Puttalam District**  Andigama  Egodapitiya  Coconut Research Institute, Lunuwila (Lunwila)  Vannativillu (Near Puttalam Area)  Madurankuliya  Marawila  Pallama  Panirendawa Forest  Puttalam (Puttalama)  Ranweli  Waikkal (Waikkala)  **Trincomalee District**  Kantale (Kantalai)  Trincomalee (Trincomali) | **Badulla District**  Diyatalawa (DyYatalawa)  Dunhinda Falls  Gavarammana (Gavaramana)  Haputale  Koslanda  Namunukula (Namunakuli, Namunaculi)  Pattiyagedara (Palliyagedera)  **Kurunegala District**  Egodayagama  Kumbukweva Forest  Kurunegala  Mawathagama  **Matale District**  Matale  Moraella  Nalanda (Naianda)  Rambukoluwa |
